# Genetic diversity and evolution of human metapneumovirus fusion protein over twenty years

**DOI:** 10.1186/1743-422X-6-138

**Published:** 2009-09-09

**Authors:** Chin-Fen Yang, Chiaoyin K Wang, Sharon J Tollefson, Rohith Piyaratna, Linda D Lintao, Marla Chu, Alexis Liem, Mary Mark, Richard R Spaete, James E Crowe, John V Williams

**Affiliations:** 1 Department of Pediatrics, Vanderbilt University School of Medicine, Nashville, TN, USA; 2 Monroe Carell Jr Children's Hospital at Vanderbilt, Nashville, TN, USA; 3 Department of Microbiology and Immunology, Vanderbilt University School of Medicine, Nashville, TN, USA; 4 MedImmune Vaccines, Inc, Mountain View, CA, USA

## Abstract

**Background:**

Human metapneumovirus (HMPV) is an important cause of acute respiratory illness in children. We examined the diversity and molecular evolution of HMPV using 85 full-length F (fusion) gene sequences collected over a 20-year period.

**Results:**

The F gene sequences fell into two major groups, each with two subgroups, which exhibited a mean of 96% identity by predicted amino acid sequences. Amino acid identity within and between subgroups was higher than nucleotide identity, suggesting structural or functional constraints on F protein diversity. There was minimal progressive drift over time, and the genetic lineages were stable over the 20-year period. Several canonical amino acid differences discriminated between major subgroups, and polymorphic variations tended to cluster in discrete regions. The estimated rate of mutation was 7.12 × 10^-4 ^substitutions/site/year and the estimated time to most recent common HMPV ancestor was 97 years (95% likelihood range 66-194 years). Analysis suggested that HMPV diverged from avian metapneumovirus type C (AMPV-C) 269 years ago (95% likelihood range 106-382 years).

**Conclusion:**

HMPV F protein remains conserved over decades. HMPV appears to have diverged from AMPV-C fairly recently.

## Background

Human metapneumovirus (HMPV) is a recently described respiratory virus in the order *Mononegavirales*, family *Paramyxoviridae*, subfamily *Pneumovirinae*, genus *Metapneumovirus *[1]. HMPV is a leading cause of lower respiratory infection (LRI) in infants and children worldwide [2-13]. HMPV is also associated with severe disease in immunocompromised hosts or persons with underlying conditions [14-20]. Most reports of HMPV molecular epidemiology have included only a few seasons, and the genetic variability of HMPV over decades has not been determined. Candidate vaccines for HMPV are under development [21-25], and the fusion (F) protein is the major antigenic determinant of protection [22,24,26-28] Therefore, it is critical to understand the potential for immune escape through virus evolution over time, and the likelihood that immunity against a particular F protein included in a vaccine candidate will be broadly protective.

The virus most closely related genetically to HMPV is avian metapneumovirus type C (AMPV-C) [1]. AMPV is an emerging pathogen of poultry that was identified in 1979. Subtypes AMPV-A and AMPV-B circulate in Europe and Africa, while AMPV-C was discovered in Minnesota and has been detected in the US and Korea [29,30]. Productive experimental infection of poultry with HMPV has not been successful, and serological studies have failed to detect evidence of human infection by AMPV [1]. Recent data suggest that F protein is responsible for this species restriction [31]. Thus, HMPV infection of humans may arise from a relatively recent trans-species transmission from AMPV-C.

We analyzed full-length F gene sequences from 68 isolates of HMPV collected over a 20-year period from otherwise healthy children with respiratory disease and 17 published full-length F gene sequences from other regions of the world. Our data show that HMPV F is highly conserved geographically over several decades. Distinct amino acid changes were present between different genetic lineages, but these amino acids were conserved within lineages. Variations that were present clustered in discrete regions, suggesting antigenic sites possibly driven by selective immune pressure. However, HMPV F gene sequences did not display progressive drift over time, unlike influenza viruses. The mutation rate of HMPV was similar to that of other RNA viruses, and the time to most recent common ancestor suggested recent divergence from AMPV-C.

## Results

### Comparison of sequence identity between subgroups

Full-length F gene sequences were obtained for 68 Tennessee strains of HMPV and assigned to one of the four proposed lineages (A1, A2, B1, or B2) based on phylogenetic analysis, discussed further below [32]. Of the 68 strains sequenced, 34 (50%) were of the B2 lineage, 18 (26%) A2, 7 (10%) B1 and 9 (13%) A1 lineage. Sequences obtained in this study were compared to 17 published full-length HMPV F gene sequences. The overall mean nucleotide identity between all 85 isolates was 89%, with a minimum identity of 83.7% (Table 1). The identity within major groups was higher, mean 96% (minimum 93.9%) between A1 and A2, and mean 97% (minimum 93.5%) between B1 and B2. The B2 lineage diverged more from the A lineages than the B1 lineage. B2 mean identity with A1 and A2 was 86.7% and 89.7%, respectively, while B1 identity with A1 and A2 was 91.3% and 94.7%, respectively. Mean nucleotide identity was >97% within all minor lineages, although the minimum identity for the B2 isolates was the lowest at 93.5%, showing more diversity within this lineage.

**Table 1:** Comparison of nucleotide and amino acid identity of full-length human metapneumovirus F genes within or between subgroups.

**Group**	**Number of sequences**	**Minimum % nt identity**	**Mean %** **nt identity**	**Minimum % aa identity**	**Mean %** **aa identity**
A1	13	97.5	98.2	99.3	99.6
A2	23	97.2	98.7	98.9	99.6
All A1+A2	36	93.9	96	98	98.7
B1	11	97.6	98.5	98.7	99.3
B2	38	93.5	97.5	99.4	99.9
All B1+B2	49	93.5	97	98.7	99.3
All A1+B1	24	84	91.3	93.7	97
All A1+B2	51	83.7	86.7	94.2	95.7
All A2+B1	34	84	94.7	93.9	98.1
All A2+B2	61	84.1	89.7	94.6	96.7
All	85	83.7	89	93.7	96.3

nt = nucleotide; aa = amino acid.

Amino acid identity was more conserved than nucleotide identity between and within all groups, with overall minimum identity of 93.7% and mean identity 96.3%. Amino acid identity within major groups was 98.7% for A1 and A2, and 99.3% for B1 and B2. The minimum amino acid identity between all lineages was approximately 94%; the greater divergence of the B2 lineage at the nucleotide level was not represented in the amino acid sequence.

### Distinct and conserved amino acid changes between lineages

**Figure 1:**
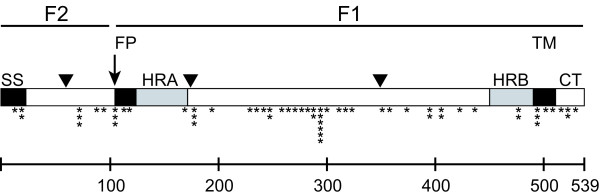
**Schematic representation of putative structure and mutation map of human metapneumovirus F protein**. SS = signal sequence; FP = fusion peptide; HRA = heptad repeat A; HRB = heptad repeat B; TM = transmembrane domain; and CT = cytoplasmic tail. Arrow indicates cleavage site; arrowheads indicate putative N-glycosylation sites. Amino acid variations are indicated by asterisks, with the number of asterisks representing the number of distinct strains in which the variation was found.

There were a number of amino acid residues distinct to each group or subgroup (Table 2). The greatest number of divergent and subgroup-specific residues was identified in the F1 domain, between the two heptad repeat (HR) regions. At several positions all subgroups had either arginine or lysine but maintained a basic residue: 82, 348, 450, 479 and 518; only position 82 has been shown to be cleaved during infection [33,34]. Many subgroup-specific residues were similar biochemically between groups. Some variations, however, were unexpected, such as the presence of a proline at position 404 only in B subgroup viruses. Fourteen cysteine residues were conserved among all isolates except one Japanese sequence (JPS03.178) with a reported C292W variation [35]. Three potential N-glycosylation sites were conserved in all sequences: N58, N172 and N350 (Figure 1).

**Table 2:** Comparison of distinct amino acid variations in the indicated functional domains of F protein between groups or subgroups of unique human metapneumovirus strains.

**Functional domain**	**AA residues in domain**	**No. of AA**	**AA Position**	**A** **(n = 36)**	**B** **(n = 49)**	**A1** **(n = 13)**	**A2** **(n = 23)**	**B1** **(n = 11)**	**B2** **(n = 38)**
Signal peptide	1-22	2	6	V	M/V	V	V	M/V	M/V
			9	F	I				
F2 subunit	23-102	2	61	A/S	T/(S*)	A	A/S	T	T/(S*)
			82			R	K	K	K
Fusion peptide	103-125	1	122	V	I				
Heptad repeat A	131-172	4	135	T	N				
			139	N	G				
			143			K	K/T	Q/(K*)	K/T
			167	D	E				
F1 subunit	173-453	10	175	R	S				
			185	A/D	A	A	D	A	A
			233	N	Y				
			286	V	I				
			296	K/R	N/D	K	K/R	N/(D*)	D
			312	Q	K				
			348	K	R				
			404	N/S	P	N	N/S	P	P
			449	V/I	I	V	V/I	I	I
			450	K	R/K				
Heptad repeat B	454-486	3	466	S/N	S	S	N/S	S	S
			479	R	K/(R*)	R	R	K/(R*)	K
			482	S/(N*)	N/(S*)	S	S/(N*)	N/(S*)	N
Transmembrane	490-514	6	498	I	I/V	I	I	V	I
			503	S	L				
			504	T/S	T/A	T	S	T	T/A
			507	L	S				
			510	V/I	I	V	V/I	I	I
			511	F	I				
Cytoplasmic tail	515-539	4	518	K/(R*)	R/(K*)	K	K/(R*)	R/(K*)	R
			528	S	N				
			533	N	G				
			539			N/(S*)	S/N	S	S

Amino acids (AA) in bold type did not vary within the group or subgroup.

*Amino acids found in only one isolate within the group or subgroup.

There were a number of single amino acid variations present in only one or a few sequences; these amino acids are listed in Table 3 and shown graphically in Figure 1. Many of these variant amino acids were biochemically quite dissimilar, though the biological significance of this finding is not clear. Interestingly, the highest variability was in the region between amino acids 260 to 300, analogous to the major antibody antigenic site A of the related RSV F protein [36] (Figure 1). Some of the variations in this region, such as E294G, were present in viruses of both the A1 and A2 subgroups. Viruses of the A2 lineage had the greatest number of such variations in the region between amino acids 230 to 300, but not elsewhere in the protein.

**Table 3:** Distinct amino acid variations detected in the indicated domains of F protein in human metapneumovirus strains.

**Domain**	**AA positions of domain**	**Subgroup**			
		
		A1	A2	B1	B2
		
Signal peptide	1-22	K20Q		S21N	S21N
F2 subunit	23-102		D72E (3)		
		E93V	E96G		
Cleavage site	102-103	S101P (2)*		S101P	
Fusion peptide	103-125		T114A		A115T
Heptad repeat A	131-172		K172R		
F1 subunit	173-453		K179Q	R179K (2)	
			S232P	F196Y	
		I248F	G239E		
		L249P (2)	G261E		
			M270T		
			V271I	D280G	
			C292G	I285T	
			C292W		
		E294G (4)	E294G (2)		
			K296R (3)		
			N298S		
			Y310N		
			A314T		E323K
			I352V		N358K
			H368N	R396W (2)	
			N404S (2)		
			T419I	K438R	
Heptad repeat B	454-486			D475E (2)	
Transmembrane	490-514	I492T	I492V	I492V	
		I514T	L507P		
Cytoplasmic tail	515-539	K519R	P520Q	P525L	P520T

* Numbers in parentheses indicate the number of distinct strains with the variation.

### Phylogenetic diversity and evolution over time

**Figure 2:**
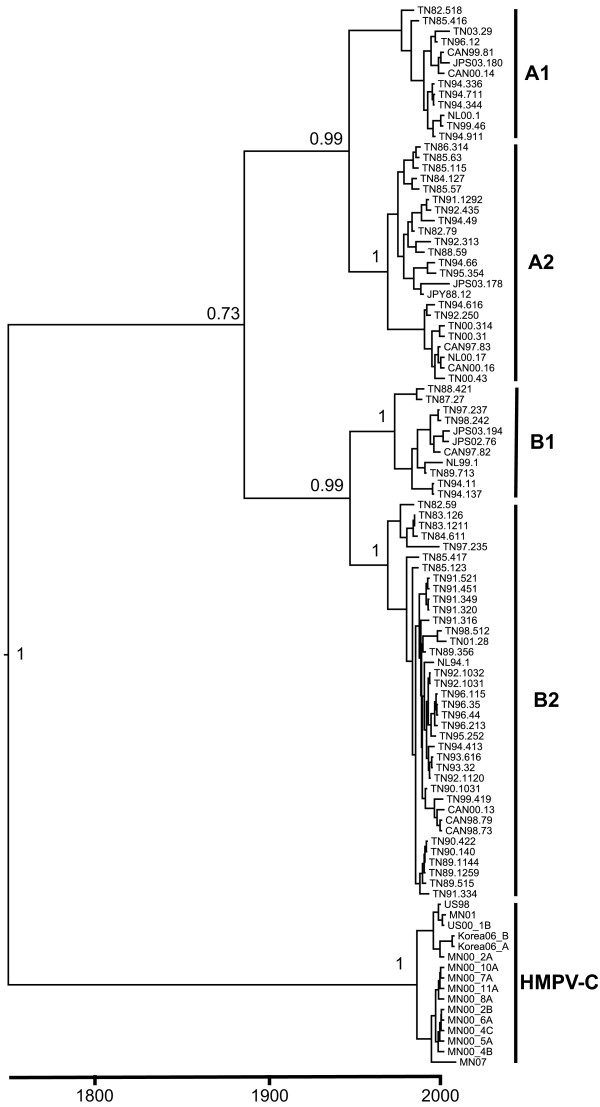
**Maximum clade credibility tree of HMPV and AMPV F nucleotide diversity by tMRCA**. Phylogenetic analysis of 85 full-length HMPV F nucleotide sequences from Canada (CAN), Japan (JPS or JPY), Tennessee (TN), or the Netherlands (NL) and 16 AMPV F sequences. The first two digits of the HMPV sequence names indicate the year of the isolate. The names of the AMPV sequences indicate geographic origin (US = United States; UK = United Kingdom; MN = Minnesota) and year. The posterior probability of divergence is indicated at each node. Mean TMRCA nodes on the MCC tree differ slightly from those reported in text, although all are contained with the same 95% HPD values. Scale bar represents time in years. Tree was constructed as described in Methods.

We performed phylogenetic and evolutionary analysis of the aligned full-length F sequences with six different models using the BEAST program suite [37]. The phylogenetic tree representing the sequence relationships by nucleotide substitutions identified four genetic subgroups (Figure 2), consistent with previous analyses [32]. The four distinct subgroups remained stable over time, and viruses within these lineages were closely related genetically, despite being isolated at time points separated by as many as twenty years. Thus, the clustering did not correlate closely with chronological origin of the sequences. For example, one subcluster within the B2 lineage contained nearly identical sequences from Tennessee in 1989, 1990, 1991, 1992, 1993, 1994, 1995, 1996, 1998, 1999 and 2001, as well as Netherlands in 1994 and Canada in 1998 and 2000 (Figure 2). Similar clustering of chronologically and geographically disparate sequences was present within each subgroup. In the A1 subgroup, Tennessee sequences from 1994, 1996, and 2003 were closely related to Canadian sequences from 1999 and 2000 and a Japanese sequence from 2003. To examine further the evolution of HMPV F gene sequences over time, we aligned sequences within each subgroup in chronological order (see Additional files 1, 2, 3 and 4). A few nucleotide changes persisted in later chronological viruses and thus represented progressive evolution at those sites. However, the majority of the nucleotide changes from year to year were not preserved and often reverted in subsequent isolates, showing a lack of major drift over time.

Analysis of multiple sequences collected over time allowed a molecular clock calculation of viral nucleotide changes. The mutation rate of HMPV F was 7.12 × 10^-4 ^substitutions/site/year (95% HPD 4.23 × 10^-4^, 1.01 × 10^-3^). The estimated time to most recent common ancestor (tMRCA) of all HMPV strains was 97 years (95% HPD 66-194) (Figure 2). The estimated time of divergence of the A subgroup into A1 and A2 was 51 years (95% HPD 38-92) and between B1 and B2 subgroups 40 years (95% HPD 38-97). Similar analysis using the limited number of available AMPV full-length F sequences (n = 24, including 16 AMPV-C F sequences collected between 1998-2007) suggested a tMRCA between AMPV-C and HMPV of 269 years (95% HPD 106-382) (Figure 2). However, very few full-length AMPV type C F sequences were available, and most were obtained within the last few years. The effect of these limitations is reflected in the wide 95% HPD intervals and thus the estimates for divergence of HMPV from AMPV-C must be considered with some caution.

### Discussion

We analyzed 85 full-length HMPV F gene sequences obtained over a twenty-year period from Tennessee, Canada, Japan, and the Netherlands. Our data confirm that there are four distinct genetic lineages of HMPV, provisionally designated as A1, A2, B1 and B2 [32]. These data further show that these genetic subgroups are stable over time in circulating viruses in a population of children with respiratory illnesses. Thus, HMPV does not appear to exhibit progressive genetic evolution, unlike influenza virus that exhibits rapid genetic drift associated with antigenic variation resulting in immune escape. In this respect, HMPV appears to be similar to other paramyxoviruses. RNA viruses mutate frequently due to the infidelity and lack of proofreading ability of RNA-dependent RNA polymerases [38]. Our data confirm that the HMPV polymerase also allows frequent errors resulting in the circulation of field strains with nucleotide variations at a similarly high rate. The rate of mutations we identified in HMPV F (7.12 × 10^-4 ^substitutions/site/year) was intermediate between the lower rate of measles virus H gene mutation (9 × 10^-5 ^substitutions/site/year) and the higher rate of influenza A virus HA (1.8 × 10^-3 ^substitutions/site/year)[39]. Nonetheless, while paramyxoviruses including RSV and measles exhibit mutations and genotype variation over time [40,41], these nucleotide mutations do not result in progressive antigenic "drift" over time with loss of neutralizing epitopes [36,42,43]. This finding is in contrast to the data from studies of the influenza virus hemagglutinin protein, which progressively evolves both genetically and antigenically, necessitating annual vaccine updates [44-47]. The reason for the lack of directional antigenic drift in paramyxoviruses is not clear. There could be functional constraints on paramyxovirus fusion proteins to prevent such drastic amino acid changes. The fact that nucleotide diversity is greater than amino acid diversity among HMPV F sequences supports this hypothesis. In contrast to paramyxoviruses, the analogous influenza virus hemagglutinin and human immunodeficiency virus gp120 fusion proteins are capable of substantial mutation to escape neutralizing antibodies without loss of function. Alternatively, the nature of immune pressure on fusion protein sequences by human antibodies could differ between paramyxoviruses and orthomyxoviruses. Experimental live wild-type virus challenge of previously infected adults with a single lot of virus can achieve productive infection in a repetitive fashion within months of previous infection with the same virus [48]. The mechanism of the functional constraints on paramyxovirus fusion protein diversity warrants further investigation.

The finding that HMPV F gene sequences do not evolve rapidly in a progressive fashion is important for the development of monoclonal antibodies (mAbs) and vaccines. The HMPV F protein is the major determinant of protection in animal models [21,22,24,26,28]. Studies with a limited number of virus strains in these models suggest a degree of cross-protective efficacy mediated by prior infection with viruses of differing subgroups [26,49]. The high degree of conservation of F protein over time suggests that interventions such as mAbs or vaccines likely will not need to be continuously updated.

We identified a number of group and subgroup-specific amino acid residues, some in putative functional domains. The biological importance of these variations is not clear, since definitive evidence of pathogenic differences between HMPV strains has not been described. A previous analysis of 84 partial HMPV F sequences did not identify subgroup-specific amino acid differences between A1 and A2 viruses; however, a 441-nt gene segment was analyzed and most of the viruses were of recent derivation [32]. The subgroup-specific amino acid changes in F genes also were conserved over time, raising the question of whether these residues possess critical biological features for virus infection or transmission. Some of these variant amino acids were found in regions presumed to be essential, such as the heptad repeat (HR) regions. Synthetic HR peptides mediate potent *in vitro *inhibition of HMPV infection [50,51] and the HR are predicted to form a six-membered helical bundle [50], suggesting that HMPV F is a Class I viral fusion protein. We have cultivated multiple strains of all four subgroups that exhibit similar growth kinetics and syncytial formation *in vitro*, and similar levels of replication *in vivo *in rodents (data not shown), suggesting that the fusion function of all these strains is intact despite amino acid variations.

The HMPV F protein, like other Class I viral fusion proteins, requires cleavage for activation and most strains require exogenous trypsin for in vitro growth. Schickli et al described a cleavage site mutation S101P that arose in two strains of HMPV during cell passage and was associated with trypsin-independent viral growth *in vitro *[34]. The variant viruses did not differ from wild type in replication in Syrian hamsters [34]. We identified an S101P variation in three distinct viruses in this study from 1989, 1994, and 1999. The F gene sequences in the current study were amplified directly from specimens collected from children with URI, and thus these viruses are natural variants. One of these had an associated E93V variation that also was observed by Schickli et al. None of these three viruses in our study was associated with more severe clinical disease (data not shown).

Human and rodent F-specific mAbs have been described with neutralizing activity *in vitro *and protective effects *in vivo*, and several overlapping antigenic sites have been identified using these mAbs [52,53]. However, the precise location of these epitopes on the protein has not been defined. We find it intriguing that the greatest concentration of amino acid variations among these 85 field isolates lies in a region found between residues 260 to 300, which is roughly analogous to the major antibody antigenic site A in the human RSV F protein [36,42]. The precise definition of neutralizing epitopes, especially conserved epitopes for broadly neutralizing antibodies, is critical for the development of prophylactic mAbs.

Phylogenetic and evolutionary analysis of multiple full-length HMPV and AMPV F sequences obtained over twenty years showed that HMPV may have diverged from AMPV-C nearly 300 years ago, and the divergence of the four HMPV genotypes likely occurred within the last hundred years. de Graaf et al recently reported estimated tMRCA values of ~120 years for the four HMPV genotypes and 200 years for HMPV divergence from AMPV-C [54]. These estimates were based on analysis of 76 HMPV G sequences, 107 partial HMPV F sequences, 12 partial AMPV-C F sequences, 21 HMPV N sequences, and 15 AMPV-C N sequences from isolates collected over approximately 12 years. Thus, the number of genes included was greater, but the spread in years was less and most sequences were from recent isolates. Despite these differences, we estimated remarkably similar rates of divergence for both major and minor subgroups. Padhi et al analyzed published HMPV G sequences and estimated a tMRCA of only 25-50 years; however, the majority of viruses in that study were isolated between 2001 and 2003 [55]. Analysis of complete genome sequences from HMPV strains obtained over many years would provide the most robust estimates of genetic diversity and evolution.

Our phylogenetic and evolutionary analysis suggest that HMPV may have diverged fairly recently from AMPV, although the power of this analysis was limited by the small number of available AMPV F gene sequences. Successful productive infection of chickens and turkeys with HMPV has not been reported [1], although inflammation, HMPV RNA and antigen could be detected in turkey poults inoculated with a large inoculum of HMPV [56]. HMPV and AMPV contain analogous open reading frames in the same order that are distinct from those of the *Pneumovirus *genus, and metapneumoviruses lack the NS1 and NS2 genes of pneumoviruses [57]. This finding suggests that HMPV diverged from AMPV-C. Other viruses including influenza and HIV are thought to have originated in animal reservoirs but are now established primary human pathogens; HMPV may have arisen as a human pathogen by similar zoonotic transfer.

## Methods

### HMPV isolates

Virus sequences were derived from specimens collected over a twenty-year period from 1982-2002 in the Vanderbilt Vaccine Clinic, as previously described [2,3]. Nasal wash specimens were collected from children <5 years of age with acute respiratory tract illness. We extracted RNA from these samples and used quantitative real-time RT-PCR to test for HMPV by detection of nucleoprotein gene sequences [2]. Specimens that tested positive for HMPV were subjected to nested RT-PCR for the F gene as described below. Viral nomenclature used in this study uses a letter code representing the geographic site of isolation (*e.g*., "TN" represents Tennessee) followed by the year of isolation, month in which the virus was isolated and isolate number.

### RNA extraction, RT-PCR and sequencing of F genes

RNA was extracted from 220 μl of nasal wash sample on a Qiagen BioRobot 9604 Workstation using the QIAamp Viral RNA kit (Qiagen), as described [2]. Amplification of the entire F open reading frame (ORF) was carried out by RT-PCR followed by nested PCR. The primers used to amplify the F regions were FF1 (5'-ATGTCTGTACTTCCCAAA-3') and FR (5'-CCCGYACTTCATATTTGCA-3') for RT-PCR, and FF2 (5'-AATATGCAAGACTTGGAGCC-3' and 5'-AGGATCTGCAAGAGCTGGAG-3') and FR (5'-CCCGYACTTCATATTTGCA-3') for nested PCR. The Thermoscript/Platinum Taq Polymerase Kit (Invitrogen) was used in a 50 μL RT-PCR reaction with 10 μL of diluted RNA as template. The RT-PCR was carried out at 50°C for 50 min and 95°C for 3 min, followed by 5 cycles of 94°C for 30 sec, 50°C for 1 min, and 68°C for 3 min, and additional 30 cycles of 94°C for 30 sec, 55°C for 1 min, and 68°C for 3 min. For nested PCR, 2 μL of RT-PCR product was added to a 50 μL reaction using Platinum PCR Supermix (Invitrogen). The reaction was incubated at 95°C for 3 min followed by 5 cycles of 94°C for 30 sec, 50°C for 30 sec, and 68°C for 2 min, and additional 30 cycles of 94°C for 30 sec, 55°C for 30 sec, and 68°C for 2 min. For all reactions a final extension at 68°C for 7 min was included. The resulting products were about 1.9 kb for the F ORF and flanking sequences. The majority of PCR products generated after RT-PCR and nested PCR were specific and migrated as a single band of the expected size (data not shown). Agarose gel purification of the desired PCR products was performed when multiple products were generated.

Sequencing reactions were carried out using ABI PRISM BigDye Terminator Cycle Sequencing Ready Reaction Kit (Applied Biosystems). Eight sequencing primers were used for each fragment to ensure a two-fold coverage of the open reading frame. Sequencing primers are available upon request. The products were processed by capillary electrophoresis using ABI 3730 DNA Analyzer (Applied Biosystems), and analyzed using DNA Sequencing Analysis (Applied Biosystems) and Sequencher (Gene Codes Corp.).

### Sequence alignment and phylogenetic analysis

Final sequences were edited and aligned using the ClustalW algorithm in MacVector version 10.0 (Accelrys) and MEGA version 3.1 [58]. Published AMPV and HMPV F sequences were obtained from GenBank (Accession numbers AY145287-AY145301, AY304360-AY304362, AYAY622381, EF051124, EF081369, EF199771-EF199772, EF589610, AF176593, AF187153-AF187154, AF298642-AF298650, AF368170, AF085228, AJ400728, AJ400730, DQ175630-DQ175634, DQ207607, D00850, EU658938, Y14290-Y14294). Sequences identified in this study have been submitted to GenBank under accession numbers EU857542-EU857610. Pairwise sequence alignment, multiple sequence alignment, and percent nucleotide identity calculations were performed using MacVector version 9.0. Inference of phylogeny and overall rates of evolutionary change (nucleotide substitutions per site per year) and the time to most recent common ancestor (tMRCA) were estimated using the Bayesian Markov chain Monte Carlo (MCMC) approach available in the BEAST package [37]. Because the sequences analyzed were very closely related and exhibited few multiple substitutions at single nucleotide sites, we used the simple HKY85 model of nucleotide substitution in each case, as more complex models sometimes failed to converge (data not shown). Data sets were analyzed under demographic models of constant population size, exponential population growth, and expansion population growth, using strict or relaxed (uncorrelated logarithmic) molecular clocks. Comparison of the output of each model showed that the relaxed clock, exponential population growth model gave the best estimation based on 95% highest posterior density (HPD)(not shown). All runs were visually examined to ensure convergence and Estimated Sample Size of >200. MCMC chains were run for 30 million steps with a burn-in rate of 10%, and two separate runs were combined using the Log Combiner program [37], with uncertainty in parameter estimates reported as the 95% HPD. Output sets of trees were combined using LogCombiner and analyzed with the TreeAnnotator program to produce a Maximum Clade Credibility tree with a posterior probability limit of >50%. Final tree was produced with FigTree [37].

## Competing interests

Chin-Fen Yang, Chiaoyin K. Wang, Linda Lintao, Marla Chu, Alekis Liem, Mary Mark and Richard R. Spaete were employees of MedImmune at the time of this study. James E. Crowe, Jr. has served as a consultant for Anaptys, Immunobiosciences, Mapp, MedImmune, and Novartis and has had research support from MedImmune, Mapp, Alnylam, and sanofi Pasteur. John V. Williams has served as a consultant for MedImmune and Novartis.

## Authors' contributions

CFY, CKW, LDL, MC, AL, MM, RRS, and RP performed RT-PCR, cloning and sequencing of HMPV isolates. SJT cultivated HMPV isolates and performed RT-PCR, cloning and sequencing. JVW and JEC conceived the study, participated in its design and coordination, and helped to draft the manuscript. JVW performed sequence alignment and phylogenetic analysis. All authors read and approved the final manuscript.

## Supplementary Material

Additional file 1 **Supplemental Figure 1**. Nucleotide sequence alignment of full-length F genes from subgroup A1 HMPV isolates, listed in chronological order. Click here for file

Additional file 2 **Supplemental Figure 2**. Nucleotide sequence alignment of full-length F genes from subgroup A2 HMPV isolates, listed in chronological order. Click here for file

Additional file 3 **Supplemental Figure 3**. Nucleotide sequence alignment of full-length F genes from subgroup B1 HMPV isolates, listed in chronological order. Click here for file

Additional file 4 **Supplemental Figure 4**. Nucleotide sequence alignment of full-length F genes from subgroup B2 HMPV isolates, listed in chronological order. Click here for file
