# Supplementary material for: Genetic diversity and evolution of human metapneumovirus fusion protein over twenty years
Source: Virol J. 2009 Sep 9;6:138. doi: 10.1186/1743-422X-6-138 (PMC2753315; doi:10.1186/1743-422X-6-138)
Supplement: Additional file 1 — Supplemental Figure 1. Nucleotide sequence alignment of full-length F genes from subgroup A1 HMPV isolates, listed in chronological order. [file 1743-422X-6-138-S1.pdf]

Supplemental Figure 1

```
TN/82/5-18 ATG TCT TGG AAA GTG GTG ATC ATT TTT TCA TTG TTA ATA ACA CCT CAA CAC GGC CTC CAG GAG AGC TAC TTA GAA GAG TCA TGT AGC ACT ATA ACT GAG  [  99]
TN/85/4-16 ... ... ... ... ... ... ... ... ... ... ... ... ... ... ... ... ... ... ..T A.. ... ... ... C.. ... ... ... ... ... ... ... ... ...  [  99]
TN/94/3-36 ... ... ... ... ... ... ... ... ... ... ... ... ... ... ... ... ... ..T ..T A.A ... ... ... ... ... ... ... ... ... ... ... ... ..A  [  99]
TN/94/3-44 ... ... ... ... ... ... ... ... ... ... ... ... ... ... ... ... ... ..T ..T A.A ... ... ... ... ... ... ... ... ... ... ... ... ..A  [  99]
TN/94/7-11 ... ... ... ... ... ... ... ... ... ... ... ... ... ... ... ... ... ..T ..T A.A ... ... ... ... ... ... ... ... ... ... ... ... ..A  [  99]
TN/94/9-11 ... ... ... ... ... ... ... ... ... ... ... ... ... ... ... ... ... ..T ..T A.A ... ... ... ... ... ... ... ... ... ... ... ... ..A  [  99]
TN/96-12   ... ... ... ... ... ... ... ... ... ... ... ... ... ... ... ... ... ..T ..T A.A ... ... ... ... ... ... ... ... ... ... ... ... ..A  [  99]
TN/99/4-6  ... ... ... ... ... ... ... ... ... ... ... ... ... ... ... ... ... ..T ..T A.A ... ... ... ... ... ... ... ... ... ... ... ... ..A  [  99]
CAN99.81   ... ... ... ... ... ... ... ... ... ... ... ... ... ... ... ... ... ..T ..T A.A ... ... ..T ... ... ... ... ... ... ... ... ... ..A  [  99]
NL/00-1    ... ... ... ... ... ... ... ... ... ... ... ... ... ... ... ... ... ..T ..T A.A ... ... ... ... ... ... ... ... ... ... ... ... ..A  [  99]
CAN00.14   ... ... ... ... ... ... ... ... ... ... ... ... ... ... ... ... ... ..T ..T A.A ... ... ..T ... ... ... ... ... ... ... ... ... ..A  [  99]
TN/03-29   ... ... ... ... ... ... ... ... ... ... ... ... ... ... ... ... ... ..T ..T A.A ... ... ... ... ... ... ... ... ... ... ... ... ..A  [  99]
JPS03.180  ... ... ... ... ... ... ... ... ... ... ... ... ... ... ... ... ... ..T ..T A.A ... ... ..T ... ... ... ... ... ... ... ... ... ..A  [  99]

TN/82/5-18 GGA TAT CTC AGT GTT CTG AGG ACA GGT TGG TAT ACC AAC GTT TTT ACA CTG GAG GTA GGT GAT GTA GAG AAC CTT ACA TGT GCT GAT GGA CCT AGC TTA  [ 198]
TN/85/4-16 ... ... ... ... ... ... ... ... ... ... ... ... ... ... ... ... ... ... ... ... ... ... ... ... ... ... ... ... ... ... ... ... ...  [ 198]
TN/94/3-36 ... ... ... ... ... ... ... ... ... ... ... ... ... ... ... ... ... ... ... ... ... ... ... ... ... ... ... ... ... ... ... ... ...  [ 198]
TN/94/3-44 ... ... ... ... ... ... ... ... ... ... ... ... ... ... ... ... ... ... ... ... ... ... ... ... ... ... ... ... ... ... ... ... ...  [ 198]
TN/94/7-11 ... ... ... ... ... ... ... ... ... ... ... ... ... ... ... ... ... ... ... ... ... ... ... ... ... ... ... ... ... ... ... ... ...  [ 198]
TN/94/9-11 ... ... ... ... ... ... ... ... ... ... ..C ... ..T ... ... ... ... ... ... ..C ... ... ... ... ... ... ... ..C ... ... ..C ... ...  [ 198]
TN/96-12   ... ... ... ... ... ... ... ... ... ... ... ... ... ... ... ... ... ... ... ... ... ... ... ... ... ... ... ... ... ... ... ... ...  [ 198]
TN/99/4-6  ... ... ... ... ... ... ... ... ... ... ..C ... ..T ... ... ... ... ... ... ..C ... ... ... ... ... ... ... ..C ... ... ..C ... ...  [ 198]
CAN99.81   ... ... ... ... ... ... ... ... ... ... ... ... ... ... ... ... ... ... ... ... ... ... ... ... ... ... ... ... ... ... ... ... ...  [ 198]
NL/00-1    ... ... ... ... ... ... ... ... ... ... ..C ... ..T ... ... ... ... ... ... ..C ... ... ... ... ... ... ... ..C ... ... ..C ... ...  [ 198]
CAN00.14   ... ... ... ... ... ... ... ... ... ... ... ... ... ... ... ... ... ... ... ... ... ... ... ... ... ... ... ... ... ... ... ... ...  [ 198]
TN/03-29   ... ... ... ... ... ... ... ... ... ... ... ... ... ... ... ... ... ... ... ... ... ... ... ... ... ... ... ... ... ... ..C ... ...  [ 198]
JPS03.180  ... ... ... ... ... ... ... ... ... ... ... ... ... ... ... ... ... ... ... ... ... ... ... ... ... ... ... ... ... ... ... ... ...  [ 198]

TN/82/5-18 ATA AAA ACA GAA TTA GAC CTG ACC AAA AGT GCA CTA AGA GAG CTC AGA ACA GTT TCT GCT GAT CAA CTG GCA AGA GAG GAA CAA ATT GAG AAT CCC AGA  [ 297]
TN/85/4-16 ... ... ... ... ... ... ... ... ... ... ... ... ... ... ... ... ... ... ... ... ... ... ... ... ... ... ... ... ... ... ... ... ...  [ 297]
TN/94/3-36 ... ... ... ... ... ... ... ... ... ... ... ... ... ... ... ... ... ... ... ... ... ... ... ... ... ... ..G ... ... ... ... ... ...  [ 297]
TN/94/3-44 ... ... ... ... ... ... ... ... ... ... ... ... ... ... ... ... ... ... ... ... ... ... ... ... ... ... ..G ... ... ... ... ... ...  [ 297]
TN/94/7-11 ... ... ... ... ... ... ... ... ... ... ... ... ... ... ... ... ... ... ... ... ... ... ... ... ... ... ..G ... ... ... ... ... ...  [ 297]
TN/94/9-11 ... ... ... ... ... ... ... ... ... ... ... ... ... ... ... ... ... ... ... ... ... ... ... ... ... ... .TG ... ... ..A ... ... ...  [ 297]
TN/96-12   ... ... ... R.. ... ... ... ... ... ... ... ... ... ... ... ... ... ... ... ... ... ... ... ... ... ... ..G ... ... ... ... ... ...  [ 297]
TN/99/4-6  ... ... ... ... ... ... ... ... ... ... ... ... ... ... ... ... ... ... ... ... ... ... ... ... ... ... ..G ... ... ..A ... ... ...  [ 297]
CAN99.81   ... ... ... ... ... ... ... ... ... ... ... ... ... ... ... ... ... ... ... ... ... ... ... ... ... ... ..G ... ... ... ... ... ...  [ 297]
NL/00-1    ... ... ... ... ... ... ... ... ... ... ... ... ... ... ... ... ... ... ... ... ... ... ... ... ... ... ..G ... ... ..A ... ... ...  [ 297]
CAN00.14   ... ... ... ... ... ... ... ... ... ... ... ... ... ... ... ... ... ... ... ... ... ... ... ... ... ... ..G ... ... ... ... ... ...  [ 297]
TN/03-29   ... ... ... ... ... ... ... ... ... ... ... ... ... ... ... ... ... ... ... ... ... ... ... ... ... ... ..G ... ... ... ... ... ...  [ 297]
JPS03.180  ... ... ... ... ... ... ... ... ... ... ... ... ... ... ... ... ... ... ... ... ... ... ... ... ... ... ..G ... ... ... ... ... ...  [ 297]

TN/82/5-18 CAA TCT AGA TTC GTT CTA GGA GCA ATA GCA CTC GGT GTT GCA ACA GCA GCT GCA GTT ACA GCA GGT GTT GCA ATT GCC AAA ACC ATC CGG CTT GAA AGT  [ 396]
TN/85/4-16 ... ... ... ... ... ... ... ... ... ... ... ... ... ... ... ... ... ... ... ... ... ... ... ... ... ... ... ... ... ... ... ... ...  [ 396]
TN/94/3-36 ... ... ... ... ... ... ... ... ... ... ... ... ... ... ... ... ... ... ... ... ... ... ... ... ... ... ... ... ... ... ... ... ...  [ 396]
TN/94/3-44 ... ... ... ... ... ... ... ... ... ... ... ... ... ... ... ... ... ... ... ... ... ... ... ... ... ... ... ... ... ... ... ... ...  [ 396]
TN/94/7-11 ... ... ... ... ... ... ... ... ... ... ... ... ... ... ... ... ... ... ... ... ... ... ... ... ... ... ... ... ... ... ... ... ...  [ 396]
TN/94/9-11 ... C.. ... ... ... ... ... ... ... ... ... ... ... ... ..T ... ... ... ... ... ... ... ... ... ... ... ... ... ... ... ... ... ...  [ 396]
TN/96-12   ... ... ... ... ... ... ... ... ... ... ... ... ... ... ... ... ... ... ... ... ... ... ... ... ... ... ... ... ... ... ... ... ...  [ 396]
TN/99/4-6  ... C.. ... ... ... ... ... ... ... ... ... ... ... ... ..T ... ... ... ... ... ... ... ... ... ... ... ... ... ... ... ... ... ...  [ 396]
CAN99.81   ... ... ... ..T ... ... ... ... ... ... ... ... ... ... ... ... ... ... ... ... ... ... ... ... ... ... ... ... ... ... ... ... ...  [ 396]
NL/00-1    ... ... ... ... ... ... ... ... ... ... ... ... ... ... ..T ... ... ... ... ... ... ... ... ... ... ... ... ... ... ... ... ... ...  [ 396]
CAN00.14   ... ... ... ..T ... ... ... ... ... ... ... ... ... ... ... ... ... ... ... ... ... ... ... ... ... ... ... ... ... ... ... ... ...  [ 396]
```

```
TN/03-29    ... ... ... ... ... ... ... ... ... ... ... ... ... ... ... ... ... ... ... ... ... ... ... ... ... ... ... ... ... ... ... ... ...  [ 396]
JPS03.180   ... ... ... ..T ... ... ... ... ... ... ... ... ... ... ... ... ... ... ... ... ... ... ... ... ... ... ... ... ... ... ... ... ...  [ 396]

TN/82/5-18 GAA GTA ACA GCA ATT AAG AAT GCC CTC AAA AAG ACC AAT GAA GCA GTA TCT ACA TTG GGG AAT GGA GTT CGA GTG TTG GCA ACT GCA GTG AGA GAG CTG  [ 495]
TN/85/4-16 ... ... ... ... ... ... ... ... ... ... ... ... ... ... ... ... ... ... ... ... ... ... ... ... ... ... ... ... ... ... ... ... ...  [ 495]
TN/94/3-36 ... ... ... ... ... ... ... ... ... ... ... ... ... ... ... ... ... ... ... ... ... ... ... ... ... ... ... ... ... ... ... ... ...  [ 495]
TN/94/3-44 ... ... ... ... ... ... ... ... ... ... ... ... ... ... ... ... ... ... ... ... ... ... ... ... ... ... ... ... ... ... ... ... ...  [ 495]
TN/94/7-11 ... ... ... ... ... ... ... ... ... ... ... ... ... ... ... ... ... ... ... ... ... ... ... ... ... ... ... ... ... ... ... ... ...  [ 495]
TN/94/9-11 ... ... ... ... ... ... ... ... ... ... ... ... ... ... ... ... ... ... ... ... ... ... ... ..T ... ... ... ... ... ... ... ... ...  [ 495]
TN/96-12    ... ... ... ... ... ... ... ... ... ... ... ... ... ... ... ... ... ... ... ... ... ... ... ... ... ... ... ... ... ... ... ... ...  [ 495]
TN/99/4-6   ... ... ... ... ... ... ... ... ... ... ... ... ... ... ... ... ... ... ... ... ... ... ... ..T ... ... ... ... ... ... ... ... ...  [ 495]
CAN99.81    ... ... ... ... ... ... ... ... ... ... ... ... ... ... ... ... ... ... ... ... ... ... ... ... ... ... ... ... ... ... ..G ... ...  [ 495]
NL/00-1     ... ... ... ... ... ... ... ... ... ... ... ... ... ... ... ... ... ... ... ... ... ... ... ..T ... ... ... ... ... ... ... ... ...  [ 495]
CAN00.14    ... ... ... ... ... ... ... ... ... ... ... ... ... ... ... ... ... ... ... ... ... ... ... ... ... ... ... ... ... ... ..G ..A ...  [ 495]
TN/03-29    ... ... ... ... ... ... ... ... ... ... ... ... ... ... ... ... ... ... ... ... ... ... ... ... ... ... ..G ... ... ... ... ... ...  [ 495]
JPS03.180   ... ... ... ... ... ... ... ... ... ... ... ... ... ... ... ... ... ... ... ... ... ... ... ... ... ... ... ... ... ... ..G ... ...  [ 495]

TN/82/5-18 AAA GAT TTT GTG AGC AAG AAT TTA ACT CGT GCA ATC AAC AAA AAC AAG TGT GAC ATT GCT GAC CTG AAA ATG GCC GTT AGC TTC AGT CAA TTC AAC AGA  [ 594]
TN/85/4-16 ... ... ... ... ... ... ... ... ... ... ... ... ... ... ... ... ..C ... ... ... ... ... ... ... ... ... ... ... ... ... ... ... ...  [ 594]
TN/94/3-36 ... ... ... ... ... ... ... C.. ..A ... ... ... ... ... ... ... ..C ... ... ... ... ... ... ... ... ... ... ... ... ... ... ... ...  [ 594]
TN/94/3-44 ... ... ... ... ... ... ... C.. ..A ... ... ... ... ... ... ... ..C ... ... ... ... ... ... ... ... ... ... ... ... ... ... ... ...  [ 594]
TN/94/7-11 ... ... ... ... ... ... ... C.. ..A ... ... ... ... ... ... ... ..C ... ... ... ... ... ... ... ... ... ... ... ... ... ... ... ...  [ 594]
TN/94/9-11 ... ... ... ... ... ... ... C.. ..A ... ... ... ... ... ... ... ..C ... ... ... ... ... ... ... ... ... ... ... ... ... ... ... ...  [ 594]
TN/96-12    ... ... ... ... ... ... ... C.. ..A ... ... ... ... ... ... ... ..C ... ... ... ... ... ... ... ... ... ... ... ... ... ... ... ...  [ 594]
TN/99/4-6   ... ... ... ... ... ... ... C.. ..A ... ... ... ... ... ... ... ..C ... ... ... ... ... ... ... ... ... ... ... ... ... ... ... ...  [ 594]
CAN99.81    ... ... ... ... ... ... ... C.. ..A ... ... ... ... ... ... ... ..C ... ... ... ... ... ... ... ... ... ... ... ... ... ... ... ...  [ 594]
NL/00-1     ... ... ... ... ... ... ... C.. ..A ... ... ... ... ... ... ... ..C ... ... ... ... ... ... ... ... ... ... ... ... ... ... ... ...  [ 594]
CAN00.14    ... ... ... ... ... ... ... C.. ..A ... ... ... ... ... ... ... ..C ... ... ... ... ... ... ... ... ... ... ... ... ... ... ... ...  [ 594]
TN/03-29    ... ... ... ... ... ... ... C.. ..A ... ... ... ... ... ... ... ..C ... ... ... ... ... ... ... ... ... ... ... ... ... ... ... ...  [ 594]
JPS03.180   ... ... ... ... ... ..A ... C.. ..A ... ... ... ... ... ... ... ..C ... ... ... ... ... ... ... ... ... ... ... ... ... ... ... ...  [ 594]

TN/82/5-18 AGG TTT CTA AAT GTT GTG CGG CAA TTT TCA GAC AAT GCT GGA ATA ACA CCA GCA ATA TCC TTG GAC TTA ATG ACA GAT GCT GAA CTA GCC AGA GCT GTT  [ 693]
TN/85/4-16 ... ... ... ... ... ... ... ... ... ... ... ... ... ... ... ... ... ... ... ... ... ... ... ... ... ... ... ... ... ... ... ... ...  [ 693]
TN/94/3-36 ... ... ... ... ... ... ... ... ... ... ... ..C ... ... ... ... ... ... ... ... ... ... ... ... ... ... ... ... ... ... ... ... ...  [ 693]
TN/94/3-44 ... ... ... ... ... ... ... ... ... ... ... ..C ... ..G ... ... ... ... ... ... ... ... ... ... ... ... ... ... ... ... ... ... ...  [ 693]
TN/94/7-11 ... ... ... ... ... ... ... ... ... ... ... ..C ... ... ... ... ... ... ... ... ... ... ... ... ... ... ... ... ... ... ... ... ...  [ 693]
TN/94/9-11 ... ..C ... ... ... ... ... ... ... ... ... ..C ... ... ... ... ... ... ... ..T ... ... ... ... ... ... ... ... ... ... ... ... ...  [ 693]
TN/96-12    ... ... ... ... ... ... ... ... ... ... ... ... ... ... ... ... ... ... ... ... ... ... ... ... ... ... ... ... ... ... ... ... ...  [ 693]
TN/99/4-6   ... ..C ... ... ... ... ... ... ... ... ... ..C ... ... ... ... ... ... ... ..T ... ... ... ... ... ... ... ... ... ... ... ... ...  [ 693]
CAN99.81    ... ... ... ... ... ... ... ... ... ... ... ... ... ... ... ... ... ... ... ... ... ... ... ... ... ... ... ... ... ... ... ... ...  [ 693]
NL/00-1     ... ..C ... ... ... ... ... ... ... ... ... ..C ... ... ... ... ... ... ... ..T ... ... ... ... ... ... ... ... ... ... ... ... ...  [ 693]
CAN00.14    ... ... ... ... ... ... ... ... ... ... ... ... ... ... ... ... ... ... ... ... ... ... ... ... ... ... ... ... ... ... ... ... ...  [ 693]
TN/03-29    ... ... ... ... ... ... ... ... ... ... ... ... ... ... ... ... ... ... ... ... ... ... ... ... ... ... ... ... ... ... ... ... ...  [ 693]
JPS03.180   ... ... ... ... ... ... ... ... ... ... ... ... ... ... ... ... ... ... ... ... ... ... ... ... ... ... ... ... ... ... ... ... ...  [ 693]

TN/82/5-18 TCC AAC ATG CCA ACA TCT GCA GGA CAA ATA AAA CTG ATG TTG GAG AAC CGT GCA ATG GTA AGA AGA AAG GGG TTC GGA ATC CTG ATA GGA GTC TAT GGA  [ 792]
TN/85/4-16 ... ... ... ... ... ... ... ... ... ... ... ... ... ... ... ... ... ... ... ... ... ... ... ... ... ... ... ... ... ... ... ..C ...  [ 792]
TN/94/3-36 ... ... ... ... ... ... ... ... ... ... ... ... ... ... ... ... ... ... ... ... ... ... ... ... ... ... ... ... ... ... ..T ..C ...  [ 792]
TN/94/3-44 ... ... ... ... ... ... ... ... ... ... ... ... ... ... ... ... ... ... ... ... ... ... ... ... ... ... ... ... ... ... ..T ..C ...  [ 792]
TN/94/7-11 ... ... ... ... ... ... ... ... ... ... ... ... ... ... ... ... ... ... ... ... ... ... ... ... ... ... ... ... ... ... ..T ..C ...  [ 792]
TN/94/9-11 ... ... ... ... ... ... ... ... ... ... ... ... ... ... ... ... ... ... ... ... ... ... ..A ... ... ... ... ... ... ... ..T ..C ...  [ 792]
TN/96-12    ... ... ... ... ... ... ... ... ... ... ... ... ... ... ... ... ... ... ... ... ... ... ... ... ..T ... ... .C. ... ... ..T ..C ...  [ 792]
TN/99/4-6   ... ... ... ... ... ... ... ... ... ... ... ... ... ... ... ... ... ... ... ... ... ... ..A ... ... ... ... ... ... ... ..T ..C ...  [ 792]
```

```
CAN99.81   ... ... ... ... ... ... ... ... ... ... ... ... ... ... ... ... ... ... ... ... ... ... ... ... ... ... ... ... ... ... ..T ... ...  [ 792]
NL/00-1    ... ... ... ... ... ... ... ... ... ... ... ... ... ... ... ... ... ... ... ... ... ... ..A ... ... ... T.. ... ... ... ..T ..C ...  [ 792]
CAN00.14   ... ... ... ... ... ... ... ... ... ... ... ... ... ... ... ... ... ... ... ... ... ... ... ... ... ... ... ... ... ... ..T ..C ...  [ 792]
TN/03-29   ... ... ... ... ... ... ... ... ... ... ... ... ... ... ... ... ... ... ... ... ... ... ... ... ... ... ... .C. ... ... ..T ..C ...  [ 792]
JPS03.180  ... ... ... ... ... ... ... ... ... ... ... ... ... ... ... ... ... ... ... ... ... ... ... ... ... ... ... ... ... ... ..T ..C ...  [ 792]

TN/82/5-18 AGC TCC GTA ATT TAC ATG GTG CAG CTG CCA ATC TTT GGG GTT ATA GAC ACG CCT TGC TGG ATA GTA AAA GCA GCC CCT TCT TGC TCA GAA AAA AAG GGA  [ 891]
TN/85/4-16 ... ... ..T ... ... ... ... ..A ... ... ..T ... ... ... ... ... ... ... ... ... ... ... ... ... ... ... ... ... ... ... ... ... ...  [ 891]
TN/94/3-36 ... ... ... ... ... ... ... ..A ... ... ... ... ... ... ... ... ... ... ... ... ... ... ... ... ... ... ... ... ... ... ... ... ...  [ 891]
TN/94/3-44 ... ... ... ... ... ... ... ..A ... ... ... ... ... ... ... ... ... ... ... ... ... ... ... ... ... ... ... ... ... ... ... ... ...  [ 891]
TN/94/7-11 ... ... ... ... ... ... ... ..A ... ... ... ... ... ... ... ... ... ... ... ... ... ... ... ... ... ... ... ... ... ... ... ... ...  [ 891]
TN/94/9-11 ... ... ... ... ... ... ... ..A ... ... ... ... ... ... ... ... ... ... ... ... ... ... ... ... ... ... ... ..T ... .GG ... ... ...  [ 891]
TN/96-12   ... ... ... ... ... ... ... ..A ... ... ... ... ... ... ... ... ... ... ... ... ... ... ... ... ... ... ... ... ... ... ... ... ...  [ 891]
TN/99/4-6  ... ... ... ... ... ... ... ..A ... ... ... ... ... ... ... ... ... ... ... ... ... ... ... ... ... ... ... ..T ... .G. ... ... ...  [ 891]
CAN99.81   ... ... ... ... ... ... ... ..A ... ... ... ... ... ... ... ... ... ... ... ... ... ... ... ... ... ... ... ... ... .G. ... ... ...  [ 891]
NL/00-1    ... ... ... ... ... ... ... ..A ... ... ... ... ... ... ... ... ... ... ... ... ... ... ... ... ... ... ... ..T ... .G. ... ... ...  [ 891]
CAN00.14   ... ... ... ... ... ... ... ..A ... ... ... ... ... ... ... ... ... ... ... ... ... ... ... ... ... ... ... ... ... ... ... ... ...  [ 891]
TN/03-29   ... ... ... ... ... ... ... ..A ... ... ... ... ... ... ... ... ... ... ... ... ... ... ... ... ... ... ... ... ... ... ... ... ...  [ 891]
JPS03.180  ... ... ... ... ... ... ... ..A ... ... ... ... ..A ... ... ... ... ... ... ... ... ... ... ..G ... ... ... ... ... ... ... ... ...  [ 891]

TN/82/5-18 AAC TAT GCT TGC CTC TTA AGA GAA GAC CAA GGA TGG TAT TGT CAG AAT GCA GGG TCA ACT GTT TAC TAC CCA AAT GAA AAA GAC TGT GAA ACA AGA GGA  [ 990]
TN/85/4-16 ... ... ... ... ... ... ... ... ... ... ... ... ... ... ... ... ... ..A ... ... ... ... ... ... ... ... ... ... ... ... ... ... ...  [ 990]
TN/94/3-36 ... ... ... ... ... ... ... ... ..T ... ... ... ... ... ... ... ... ... ... ... ... ... ... ... ... ... ... ... ... ... ... ... ...  [ 990]
TN/94/3-44 ... ... ... ... ... ... ... ... ..T ... ... ... ... ... ... ... ... ... ... ... ... ... ... ... ... ... ... ... ... ... ... ... ...  [ 990]
TN/94/7-11 ... ... ... ... ... ... ... ... ..T ... ... ... ... ... ... ... ... ... ... ... ... ... ... ... ... ... ... ... ... ... ... ... ...  [ 990]
TN/94/9-11 ... ... ... ... ... ... ... ... ... ... ... ... ... ... ..A ... ... ... ... ... ... ... ... ... ... ... ... ... ... ... ... ... ...  [ 990]
TN/96-12   ... ... ... ... ... ... ... ... ..T ... ... ... ... ... ... ... ... ... ... ... ... ... ... ... ... ... ... ... ... ... ... ... ...  [ 990]
TN/99/4-6  ... ... ... ... ... ... ... ... ... ... ... ... ... ... ..A ... ... ... ... ... ... ... ... ... ... ... ... ... ... ... ... ... ...  [ 990]
CAN99.81   ... ... ... ... ... ... ... ... ..T ... ... ... ... ... ... ... ... ... ... ... ... ... ... ... ... ... ... ... ..C ... ... ... ...  [ 990]
NL/00-1    ... ... ... ... ... ... ... ... ... ... ... ... ... ... ..A ... ... ... ... ... ... ... ... ... ... ... ... ... ... ... ... ... ...  [ 990]
CAN00.14   ... ... ... ... ... ... ... ... ..T ... ... ... ... ..C ... ... ... ... ... ... ... ... ... ... ... ... ... ... ..C ... ... ... ...  [ 990]
TN/03-29   ... ... ... ... ... ... ... ... ..T ... ... ... ... ... ... ... ... ... ... ... ... ... ... ... ... ... ... ... ... ... ... ... ...  [ 990]
JPS03.180  ... ... ... ... ... ... ... ... ..T ... ... ... ... ... ... ... ... ..A ... ... ... ... ... ... ... ... ... ... ..C ... ... ... ...  [ 990]

TN/82/5-18 GAC CAT GTC TTT TGC GAC ACA GCA GCA GGA ATC AAT GTT GCT GAG CAG TCA AAG GAG TGC AAC ATC AAC ATA TCT ACT ACT AAT TAC CCA TGC AAA GTT  [1089]
TN/85/4-16 ... ... ... ... ... ... ... ... ... ... ... ... ... ... ... ... ... ... ... ... ... ... ... ... ..C ... ... ... ... ... ... ... ...  [1089]
TN/94/3-36 ... ... ... ... ... ... ... ... ... ... ... ... ... ... ... ... ... ... ... ... ... ... ... ... ..C ... ... ... ... ... ... ... ...  [1089]
TN/94/3-44 ... ... ... ... ... ... ... ... ... ... ... ... ... ... ... ... ... ... ... ... ... ... ... ... ..C ... ... ... ... ... ... ... ...  [1089]
TN/94/7-11 ... ... ... ... ... ... ... ... ... ... ... ... ... ... ... ... ... ... ... ... ... ... ... ... ..C ... ... ... ... ... ... ... ...  [1089]
TN/94/9-11 ... ... ... ... ... ... ... ... ... ... ... ... ... ... ... ... ... ... ... ... ... ..A ... ... ... ... ... ... ... ... ... ... ...  [1089]
TN/96-12   ... ... ... ... ... ... ... ... ... ... ... ... ..C ... ... ... ... ... ... ... ... ... ... ... ..C ... ... ... ... ... ... ... ...  [1089]
TN/99/4-6  ... ... ... ... ... ... ... ... ... ... ... ... ... ... ... ... ... ... ... ... ... ..A ... ... ... ... ... ... ... ... ... ... ...  [1089]
CAN99.81   ... ... ... ... ... ... ... ... ... ... ... ... ... ... ... ... ... ... ... ... ... ... ... ... ..C ... ... ... ... ... ... ... ...  [1089]
NL/00-1    ... ... ... ... ... ... ... ... ... ... ... ... ... ... ... ... ... ... ... ... ... ..A ... ... ... ... ... ... ... ... ... ... ...  [1089]
CAN00.14   ... ... ... ... ... ... ... ... ... ... ... ... ... ... ... ... ... ... ... ... ... ... ... ... ..C ... ... ... ... ... ... ... ...  [1089]
TN/03-29   ... ... ... ... ... ... ... ... ... ... ... ... ..C ... ... ... ... ... ... ... ... ... ... ... ..C ... ... ... ... ... ... ... ...  [1089]
JPS03.180  ... ... ... ... ... ... ... ... ... ... ... ... ... ... ... ... ... ... ... ... ... ... ... ... ..C ... ... ... ... ... ... ... ...  [1089]

TN/82/5-18 AGC ACA GGA AGA CAT CCT ATC AGT ATG GTT GCA CTG TCT CCT CTT GGG GCT TTG GTT GCT TGC TAC AAG GGA GTA AGC TGT TCC ATT GGC AGC AAC AGA  [1188]
TN/85/4-16 ... ... ... ... ... ... ... ... ... ... ... ... ... ... ... ... ... ... ... ... ... ... ... ... ... ... ..C ... ... ... ... ... ...  [1188]
TN/94/3-36 ... ... ... ... ... ... ... ... ... ... ... ... ... ... ... ... ... ... ... ... ... ... ... ... ..G ... ... ... ... ... ... ... ...  [1188]
TN/94/3-44 ... ... ... ... ... ... ... ... ... ... ... ... ... ... ... ... ... ... ... ... ... ... ... ... ..G ... ... ... ... ... ... ... ...  [1188]
TN/94/7-11 ... ... ... ... ... ... ... ... ... ... ... ... ... ... ... ... ... ... ... ... ... ... ... ... ..G ... ... ... ... ... ... ... ...  [1188]
```

```
TN/94/9-11 ... ... ... ... ... ... ... ... ... ... ... ..A ... ... ... ... ... ... ... ... ... ... ... ... ..G ... ... ... ... ... ... ... ...  [1188]
TN/96-12   ... ... ... ... ... ... ... ... ... ... ... ... ... ... ... ... ... ... ... ... ... ... ... ... ..G ... ... ... ... ... ... ... ...  [1188]
TN/99/4-6  ... ... ... ... ... ... ... ... ... ... ... ..A ... ... ... ... ... ... ... ... ... ... ... ... ..G ... ... ... ... ... ... ... ...  [1188]
CAN99.81   ... ... ... ... ... ... ... ... ... ... ... ... ... ... ... ... ... ... ... ... ... ... ... ... ..G ... ... ... ... ... ... ... ...  [1188]
NL/00-1    ... ... ... ... ... ... ... ... ... ... ... ..A ... ... ... ... ... ... ... ... ... ... ... ... ..G ... ... ... ... ... ... ... ...  [1188]
CAN00.14   ... ... ... ... ... ... ... ... ... ... ... ... ... ... ... ... ... ... ... ... ... ..T ... ... ..G ... ... ... ... ... ... ... ...  [1188]
TN/03-29   ... ... ... ... ... ... ... ... ... ... ... ... ... ... ... ... ... ... ... ... ... ... ... ... ..G ... ... ... ... ... ... ... ...  [1188]
JPS03.180  ... ... ... ... ... ... ... ... ... ... ... ... ... ... ... ... ... ... ... ... ... ... ... ... ..G ... ... ... ... ... ... ... ...  [1188]

TN/82/5-18 GTA GGG ATC ATC AAG CAA CTG AAC AAA GGC TGC TCT TAT ATA ACC AAC CAA GAT GCA GAC ACA GTG ACA ATA GAC AAC ACT GTA TAC CAG CTA AGC AAA  [1287]
TN/85/4-16 ... ... ... ... ... ... ... ... ... ... ... ... ... ... ... ... ... ... ... ... ... ... ... ... ... ... ... ... ... ... ... ... ...  [1287]
TN/94/3-36 ... ... ... ... ... ... ... ... ... ... ... ... ... ... ... ... ... ..C ... ... ... ... ... ... ... ... ... ... ... ... ... ... ...  [1287]
TN/94/3-44 ... ... ... ... ... ... ... ... ... ... ... ... ... ... ... ... ... ..C ... ... ... ... ... ... ... ... ... ... ... ... ... ... ...  [1287]
TN/94/7-11 ... ... ... ... ... ... ... ... ... ... ... ... ... ... ... ... ... ..C ... ... ... ... ... ... ... ... ... ... ... ... ... ... ...  [1287]
TN/94/9-11 ... ... ... ... ... ... ... ... ... ... ... ... ... ... ... ... ... ..C ... ... ... ... ... ... ... ... ... ... ... ... ... ... ...  [1287]
TN/96-12   ... ... ... ... ... ... ... ... ... ... ... ... ... ... ... ... ... ..C ... ... ... ... ... ... ... ... ... ... ... ... ... ... ...  [1287]
TN/99/4-6  ... ... ... ... ... ... ... ... ... ... ... ... ... ... ... ... ... ..C ... ... ... ... ... ... ... ... ... ... ... ... ... ... ...  [1287]
CAN99.81   ... ... ... ... ... ... ... ... ... ... ... ... ... ... ... ... ... ... ... ... ... ... ... ... ... ... ... ... ... ... ... ... ...  [1287]
NL/00-1    ... ... ... ... ... ... ... ... ... ... ... ... ... ... ... ... ... ..C ... ... ... ... ... ... ... ... ... ... ... ... ... ... ...  [1287]
CAN00.14   ... ... ..T ... ... ... ... ... ... ... ... ... ... ... ... ... ... ..C ... ... ... ... ... ... ... ... ... ... ... ... ... ... ...  [1287]
TN/03-29   ... ... ... ... ... ... ... ... ... ... ... ... ... ... ... ... ... ..C ... ... ... ... ... ... ... ... ... ... ... ... ... ... ...  [1287]
JPS03.180  ... ... ... ... ... ... ... ... ... ... ... ... ... ... ... ... ... ..C ... ... ... ... ... ... ... ... ... ... ... ... ... ... ...  [1287]

TN/82/5-18 GTT GAG GGC GAA CAG CAT GTT ATA AAA GGA AGG CCA GTG TCA AGC AGC TTT GAT CCA GTC AAG TTT CCT GAA GAT CAA TTC AAT GTT GCA CTT GAC CAA  [1386]
TN/85/4-16 ... ... ... ... ... ... ... ... ... ... ... ... ... ... ... ... ... ... ..T ... ... ... ... ... ... ... ... ... ... ... ... ... ...  [1386]
TN/94/3-36 ... ... ... ... ... ... ... ... ... ... ... ... ... ... ... ... ... ... ... ... ... ... ... ... ... ... ... ... ... ... ... ... ...  [1386]
TN/94/3-44 ... ... ... ... ... ... ... ... ... ... ... ... ... ... ... ... ... ... ... ... ... ... ... ... ... ... ... ... ... ... ... ... ...  [1386]
TN/94/7-11 ... ... ... ... ... ... ... ... ... ... ... ... ... ... ... ... ... ... ... ... ... ... ... ... ... ... ... ... ... ... ... ... ...  [1386]
TN/94/9-11 ... ..A ... ... ... ... ... ... ... ... ... ... ... ... ... ... ... ..C ... ... ... ... ... ... ... ... ... ... ... ... ... ... ...  [1386]
TN/96-12   ... ... ... ... ... ... ... ... ... ... ... ... ... ... ... ... ... ... ... ... ... ... ... ... ... ... ... ... ... ... ... ... ...  [1386]
TN/99/4-6  ... ..A ... ... ... ... ... ... ... ... ... ... ... ... ... ... ... ..C ... ... ... ... ... ... ... ... ... ... ... ... ... ... ...  [1386]
CAN99.81   ... ... ... ... ... ... ... ... ... ... ... ... ... ... ... ... ... ... ... ... ... ... ... ... ... ... ... ... ... ... ... ... ...  [1386]
NL/00-1    ... ..A ... ... ... ... ... ... ... ... ... ... ... ... ... ... ... ..C ... ... ... ... ... ... ... ... ... ... ... ... ... ... ...  [1386]
CAN00.14   ... ... ... ... ... ... ... ... ... ... ... ... ... ... ... ... ... ... ... ... ... ... ... ... ... ... ... ... ... ... ... ... ...  [1386]
TN/03-29   ... ... ... ... ... ... ... ... ... ... ... ... ... ... ... ... ... ... ... ... ... ... ... ... ... ... ... ... ... ... ... ... ...  [1386]
JPS03.180  ... ... ... ... ... ... ... ... ... ... ... ... ... ... ... ... ... ... ... ... ..A ... ... ... ... ... ... ... ... ... ... ... ...  [1386]

TN/82/5-18 GTT TTT GAG AGC ATT GAG AAC AGT CAG GCC TTG GTG GAT CAA TCA AAC AGA ATC CTA AGC AGT GCA GAG AAA GGG AAC ACT GGC TTC ATC ATT GTA ATA  [1485]
TN/85/4-16 ... ... ... ... ... ... ... ... ... ... ... ... ... ... ... ... ... ... ... ... ... ... ... ... ... ... ... ... ... ... ... ... ...  [1485]
TN/94/3-36 ... ..C ... ... ... ... ... ... ... ... ... ... ... ... ..T ... ... ... ... ... ... ... ... ... ..A ... ... ... ... ... ... ... ...  [1485]
TN/94/3-44 ... ..C ... ... ... ... ... ... ... ... ... ... ... ... ..T ... ... ... ... ... ... ... ... ... ..A ... ... ... ... ... ... ... ...  [1485]
TN/94/7-11 ... ..C ... ... ... ... ... ... ... ... ... ... ... ... ..T ... ... ... ... ... ... ... ... ... ..A ... ... ... ... ... ... ... ...  [1485]
TN/94/9-11 ... ..C ... ... ... ... ... ... ... ... ... ... ... ... ... ... ... ... ... ... ... ... ... ... ..A ... ... ... ... ... ... ... ...  [1485]
TN/96-12   ... ..C ... ... ... ... ... ... ... ... ... ... ... ... ... ... ... ... ... ... ... ... ... ... ..A ... ... ... ... ... ... ... ...  [1485]
TN/99/4-6  ... ..C ... ... ... ... ... ... ... ... ... ... ... ... ... ... ... ... ... ... ... ... ... ... ..A ... ... ... ... ... ... ... ...  [1485]
CAN99.81   ... ..C ... ... ... ... ... ... ... ... ... ... ... ... ... ... ... ... ... ... ... ... ... ... ..A ... ... ... ... ... ... ... ...  [1485]
NL/00-1    ... ..C ... ... ... ... ... ... ... ... ... ... ... ... ... ... ... ... ... ... ... ... ... ... ..A ... ... ... ... ... ... ... ...  [1485]
CAN00.14   ... ... ... ... ... ... ... ... ... ... ... ... ... ... ... ... ... ... ... ... ... ... ... ... ..A ... ... ... ... ... ... ... ...  [1485]
TN/03-29   ... ..C ... ... ... ... ... ... ... ... ... ... ... ... ... ... ... ... ... ... ... ... ... ... ..A ... ... ... ... ... ... ... .C.  [1485]
JPS03.180  ... ..C ..A ... ... ... ... ... ... ... ... ... ... ... ... ... ... ... ... ... ... ... ... ... ..A ... ... ... ... ... ... ... ...  [1485]

TN/82/5-18 ATT CTA ATT GCT GTC CTT GGC TCT ACC ATG ATC CTA GTG AGC GTT TTT ATC ATA ATA AAG AAA ACA AAG AAA CCT ACA GGA GCA CCT CCA GAG CTG AGT  [1584]
TN/85/4-16 ... ... ... ... ... ... ... ... ... ... ... ... ... ... ... ... ... ... ... ... ... ... ... ... ... ... ... ... ... ... ... ... ...  [1584]
```

```
TN/94/3-36 ... ... ... ... ... ... ... ... ... ... ... ... ... ..T ... ... ... ... ... ... ... ... ... ... ..C ... ... ... ... ... ... ... ...  [1584]
TN/94/3-44 ... ... ... ... ... ... ... ... ... ... ... ... ... ..T ... ... ... ... ... ... ... ... ... ... ..C ... ... ... ... ... ... ... ...  [1584]
TN/94/7-11 ... ... ... ... ... ... ... ... ... ... ... ... ... ..T ... ... ... ... ... ... ... ... ... ... ..C ... ... ... ... ... ... ... ...  [1584]
TN/94/9-11 ... ... ... ... ... ... ... ... ... ... ... ... ... ..T ... ... ... ... ... ... ... ... ... ... ..C ... ... ... ... ... ... ... ...  [1584]
TN/96-12   ... ... .Y. ... ... ..A ... ... ... ... ... ... ... ..T .Y. ... ... ... ... ... ... ... ... ... ..C ... ... ... ... ... ... ... ...  [1584]
TN/99/4-6  ... ... ... ... ... ... ... ... ... ... ... ... ... ..T ... ... ... ... ... ... ... ... ... ... ..C ... ... ... ... ... ... ... ...  [1584]
CAN99.81   ... ... ... ... ... ... ... ... ... ... ... ... ... ..T ... ... ... ... ... ... ... ... ... ... ..C ... ... ... ... ... ... ... ...  [1584]
NL/00-1    ... ... ... ... ... ... ... ... ... ... ... ... ... ..T ... ... ... ... ... ... ... ... ... ... ..C ... ... ... ... ... ... ... ...  [1584]
CAN00.14   ... ... ... ... ... ... ... ... ... ... ... ... ... ..T ... ... ... ... ... ... ... ... ... ... ..C ... ... ... ... ... ... ... ...  [1584]
TN/03-29   ... ... ... ... ... ..A ... ..C ... ... ... ... ... ..T ... ... ... ... .C. ... ... ... ... ... ..C ... ... ... ... ... ... ... ...  [1584]
JPS03.180  ... ... ... ... ... ... ... ... ... ... ... ... ... ..T ... ... ... ... ... ... ... ... ... ... ..C ... ... ... ... ... ... ... ...  [1584]

TN/82/5-18 GGT GTC ACA AAC AAT GGC TTC ATA CCA CAC AAT TAG - [1621]
TN/85/4-16 ... ... ... ... ... ... ... ... ... ..T ... ... - [1621]
TN/94/3-36 ... ... ... ... ... ... ... ... ... ..T ... ... - [1621]
TN/94/3-44 ... ... ... ... ... ... ... ... ... ..T ... ... - [1621]
TN/94/7-11 ... ... ... ... ... ... ... ... ... ..T ... ... - [1621]
TN/94/9-11 ... ... ... ... ... ... ... ... ... ..T ... ... - [1621]
TN/96-12   ... ... ... ... N.. ... ... ... ... ..T ... ... T [1621]
TN/99/4-6  ... ... ... ... ... ... ... ... ... ..T ... ... - [1621]
CAN99.81   ... ... ... ... ... ... ... ... ... ..T ... ... - [1621]
NL/00-1    ... ... ... ... ... ... ... ... ... ..T ... ... - [1621]
CAN00.14   ... ... ... ... ... ... ... ... ... ..T ... ... - [1621]
TN/03-29   ... ... ... ... ... ... ... ... ... ... .G. ... - [1621]
JPS03.180  ... ... ... ... ... ... ... ... ... ..T ... ... - [1621]
```

Supplemental figure 1. Nucleotide sequence alignment of full-length F genes from subgroup A1 HMPV isolates. Sequences are arranged in chronological order from top to bottom. The letters indicate origin: "TN" = Tennessee; "CAN" = Canada; "NL" = Netherlands; and "JPS" = Japan. The first two digits of each isolate indicate the year; the next digit(s) for TN sequences indicate month of original virus isolation. Sequences from Canada, the Netherlands and Japan obtained from GenBank as described in the text. TN sequences have been deposited in GenBank.
