# Supplementary material for: Genetic diversity and evolution of human metapneumovirus fusion protein over twenty years
Source: Virol J. 2009 Sep 9;6:138. doi: 10.1186/1743-422X-6-138 (PMC2753315; doi:10.1186/1743-422X-6-138)
Supplement: Additional file 2 — Supplemental Figure 2. Nucleotide sequence alignment of full-length F genes from subgroup A2 HMPV isolates, listed in chronological order. [file 1743-422X-6-138-S2.pdf]

Supplemental Figure 2

```
TN82.79    ATG TCT TGG AAA GTG GTG ATC ATT TTT TCA TTG CTA ATA ACA CCT CAA CAC GGT CTT AAA GAG AGC TAC TTA GAA GAA TCA TGT AGC ACT ATA ACT GAG  [  99]
TN84.127   ... ... ... ... ... ... ... ... ... ... ... ... ... ... ... ... ... ... ... ... ... ... ... ... ... ... ... ... ... ... ... ... ...  [  99]
TN85.115   ... ... ... ... ... ... ... ... ... ... ... ... ... ... ... ... ... ... ... ... ..A ... ... ... ... ... ... ... ... ... ... ... ...  [  99]
TN85.57    ... ... ... ... ... ... ... ... ... ... ... ... ... ... ... ... ... ... ... ... ... ... ... ... ... ... ... ... ... ... ... ... ...  [  99]
TN85.63    ... ... ... ... ... ... ... ... ... ... ... ... ... ... ... ... ... ... ... ... ..A ... ... ... ... ... ... ... ... ... ... ... ...  [  99]
TN86.314   ... ... ... ... ... ..A ... ... ... ... ... ... ... ... ... ... ... ... ... ... ..A ... ... ... ... ... ... ... ... ... ... ... ...  [  99]
TN88.59    ... ... ... ... ... ... ... ... ... ... ... ... ... ... ... ... ... ... ... ... ..A ... ... ... ... ... ... ... ... ... ... ... ...  [  99]
JPY88.12   ... ... ... ... ... ... ... ... ... ... ... ... ... ... ... ... ... ... ... ... ... ... ... ... ... ... ... ... ... ... ... ... ...  [  99]
TN91.1292  ... ... ... ... ... ... ... ... ... ... ... ... ... ... ... ... ... ... ... ... ... ... ... ... ... ... ... ... ... ... ... ... ...  [  99]
TN92.313   ... ... ... ... ... ... ... ... ... ... ... ... ... ... ... ... ... ... ... ... ... ... ... ... ... ... ... ... ... ... ... ... ...  [  99]
TN92.250   ... ... ... ... ... ... ... ... ... ... ... ... ... ... ... ... ... ..C ... ... ... ... ... ... ... ... ... ... ... ... ... ... ...  [  99]
TN92.435   ... ... ... ... ... ... ... ... ... ... ... ... ... ... ... ... ... ... ... ... ... ... ... ... ... ... ... ... ... ... ... ... ...  [  99]
TN94.49    ... ... ... ... ... ... ... ... ... ... ... ... ... ... ... ... ... ... ... ... ... ... ... ..G ... ... ... ... ... ... ... ... ...  [  99]
TN94.616   ... ... ... ... ... ... ... ... ... ... ... ... ... ... ... ... ... ... ... ... ... ... ... ... ... ... ... ... ... ... ... ... ...  [  99]
TN94.66    ... ... ... ... ... ... ... ... ... ... ... ... ... ... ... ... ... ... ... ... ... ... ... ... ... ... ... ... ... ... ... ... ...  [  99]
TN95.354   ... ... ... ... ... ... ... ... ... ... ... ... ... ... ... ... ... ... ... ... ... ... ... ... ... ... ... ... ... ... ... ... ...  [  99]
CAN97.83   ... ... ... ... ... ... ... ... ... ... ... ... ... ... ... ... ... ... ... ... ... ... ... C.. ... ... ... ... ... ... ... ... ...  [  99]
TN00.31    ... ... ... ... ... ... ... ... ... ... ... ..G ... ... ... ... ... ... ... ... ... ... ... C.. ... ... ... ... ... ... ... ... ...  [  99]
TN00.314   ... ... ... ... ... ... ... ... ... ... ... ..G ... ... ... ... ... ... ... ... ... ... ... C.. ... ... ... ... ... ... ... ... ...  [  99]
TN00.43    ... ... ... ... ... ... ... ... ... ... ... ... ... ... ... ... ... ... ... ... ... ... ... C.. ... ... ... ... ... ... ... ... ...  [  99]
CAN00.16   ... ... ... ... ... ... ... ... ... ... ... ... ... ... ... ... ... ... ... ... ... ... ... C.. ... ... ... ... ... ... ... ... ...  [  99]
NL00.17    ... ... ... ... ... ... ... ... ... ... ... ... ... ... ... ... ... ... ... ... ... ... ... C.. ... ... ... ... ... ... ... ... ...  [  99]
JPS03.178  ... ... ... ... ... ... ... ... ... ... ... ... ... ... ... ... ... ... ... ... ... ... ... ... ... ... ... ... ... ... ... ... ...  [  99]

TN82.79    GGA TAT CTC AGT GTT CTG AGG ACA GGT TGG TAT ACC AAC GTT TTT ACA TTA GAG GTG GGT GAT GTA GAA AAC CTC ACA TGT GCT GAT GGA CCT AGC CTA  [ 198]
TN84.127   ... ... ... ... ... ... ... ... ... ... ... ... ... ... ... ... ... ... ... ... ... ... ... ... ... ... ... ... ... ... ... ... ...  [ 198]
TN85.115   ... ... ... ... ... ... ... ... ... ... ... ... ... ... ... ..G ... ... ... ... ... ... ... ... ... ... ... ... ... ... ..C ... ...  [ 198]
TN85.57    ... ... ... ... ... ... ... ... ... ... ... ... ... ... ... ... ... ... ... ... ... ... ... ... ... ... ... ... ... ... ... ... ...  [ 198]
TN85.63    ... ... ... ... ... ... ... ... ... ... ... ... ... ... ... ..G ... ... ... ... ... ... ... ... ... ... ... ... ... ... ... ... ...  [ 198]
TN86.314   ... ... ... ... ... ... ... ... ... ... ... ... ... ... ... ..G ... ... ... ... ... ... ... ... ... ... ... ... ... ... ... ... ...  [ 198]
TN88.59    ... ... ... ... ... ... ... ... ... ... ... ... ... ... ... ... ... ... ... ... ... ... ... ... ... ... ... ... ... ... ... ... ...  [ 198]
JPY88.12   ... ... ... ... ... ... ... ... ... ... ... ... ... ... ... ... ... ... ... ... ... ... ... ... ... ... ... ... ... ... ... ... ...  [ 198]
TN91.1292  ..G ... ... ... ... ... ... ... ... ... ... ... ... ... ... ... ... ... ... ... ... ... ... ... ... ... ... ... ... ... ... ... ...  [ 198]
TN92.313   ... ... ... ... ... ..A ... ... ... ... ... ... ... ... ... ... ... ... ... ... ... ... ... ... ..T ... ... ... ... ... ... ... ...  [ 198]
TN92.250   ... ... ..T ... ... ... ... ... ... ... ... ... ... ... ... ... ... ... ... ... ... ... ... ... ... ... ..C T.. ... ... ... ... ...  [ 198]
TN92.435   ..G ... ... ... ... ... ... ... ... ... ... ... ... ... ... ... ... ... ... ... ... ... ... ... ... ... ... ... ... ... ... ... ...  [ 198]
TN94.49    ..G ... ... ... ... ... ... ... ... ... ... ... ... ... ... ... ... ... ... ... ... ... ... ... ... ... ... ... ... ... ... ... ...  [ 198]
TN94.616   ... ... ..T ... ... ... ... ... ... ... ... ... ... ... ... ... ... ... ..A ... ... ... ... ... ... ... ... T.. ... ... ... ... ...  [ 198]
TN94.66    ... ... ... ... ... ... ... ... ... ... ... ... ... ... ... ... ... ... ... ... ... ... ... ... ... ... ... ... ... ... ... ... ...  [ 198]
TN95.354   ... ... ... ... ... ... ... ... ... ... ... ... ... ... ... ... ... ... ... ... ... ... ... ... ... ... ... ... ... ... ... ... ...  [ 198]
CAN97.83   ... ... ..T ... ... ... ... ... ... ... ... ... ... ... ... ... ... ... ... ... ... ... ... ... ..T ... ... T.. ... ... ... ... ...  [ 198]
TN00.31    ... ... ..T ... ... ... ... ... ... ... ... ... ... ... ... ... ... ... ... ... ... ... ... ... ... ... ... T.. ... ... ... ... ...  [ 198]
TN00.314   ... ... ..T ... ... ... ... ... ... ... ... ... ... ... ... ... ... ... ... ... ... ... ... ... ... ... ... T.. ... ... ... ... ...  [ 198]
TN00.43    ... ... ..T ... ... ... ... ... ... ... ... ... ..T ... ... ... ... ... ... ... ... ... ... ... ... ... ... T.. ... ... ... ... ...  [ 198]
CAN00.16   ... ... ..T ... ... ... ... ... ... ... ... ... ... ... ... ... ... ... ... ... ... ... ... ... ..T ... ... T.. ... ... ... ... ...  [ 198]
NL00.17    ... ... ..T ... ... ... ... ... ... ... ... ... ... ... ... ... ... ... ... ... ... ... ... ... ..T ... ... T.. ... ... ... ... ...  [ 198]
JPS03.178  ... ... ... ... ... ... ... ... ... ... ... ... ... ... ... ... ... ... ... ... ... ... ... ... ... ... ... ... ... ... ... ... ...  [ 198]

TN82.79    ATA AAA ACA GAA TTA GAT CTG ACC AAA AGT GCA CTA AGA GAG CTC AAA ACA GTC TCT GCT GAC CAA TTG GCA AGA GAG GAA CAA ATT GAG AAT CCC AGA  [ 297]
TN84.127   ... ... ... ... ... ... ... ... ... ... ... ... ... ... ... ... ... ... ... ... ... ... ... ... ... ... ... ... ... ... ... ... ...  [ 297]
TN85.115   ... ... ... ... ... ... ... ... ... ... ... ... ... ... ... ... ... ... ... ... ... ... ... ... ... ... ... ... ... ... ... ... ...  [ 297]
TN85.57    ... ... ... ... ... ... ... ... ... ... ... ... ... ... ... ... ... ... ... ... ... ... ... ... ... ... ... ... ..C ... ... ... ...  [ 297]
TN85.63    ... ... ... ... ... ... ... ... ... ... ... ... ... ... ... ... ... ... ... ... ... ... ... ... ... ... ... ... ... ... ... ... ...  [ 297]
TN86.314   ... ... ... ... ... ... ... ... ... ... ... ... ... ... ... ... ... ... ... ... ... ... C.. ... ... ... ... ... ... ... ... ... ...  [ 297]
TN88.59    ... ... ... ... ... ... ... ... ... ... ... ... ... ... ... ... ... ... ... ... ... ... ... ... ... ... ... ... ... ... ... ... ...  [ 297]
JPY88.12   ... ... ... ... ... ... ... ... ... ... ... ... ... ... ... ... ... ..T ... ... ... ... ... ... ... ... ... ... ... ..A ... ... ...  [ 297]
```

```
TN91.1292 ... ... ..C ... ... ..A ... ... ... ... ... ... ... ... ... ... ... ... ... ... ... ... ... ..G ... ... ... ... ... ... ... ... ...  [ 297]
TN92.313  ... ... ... ... ... ... ... ... ... ... ... ... ... ... ... ... ... ... ... ... ... ... ... ... ... ... ... ... ... ... ... ... ...  [ 297]
TN92.250  ... ... ... ... ... ... ... ... ... ... ... ... ... ... ... ... ... ... ... ... ... ... ... ... ... ... ... ... ... ... ... ... ...  [ 297]
TN92.435  ... ... ... ... ... ..A ... ... ... ... ... ... ... ... ... ... ... ..T ... ... ... ... ... ..G ... ... ... ... ... ... ... ... ...  [ 297]
TN94.49   ... ... ... ... ... ..A ... ... ... ... ... ... ... ... ... ... ... ... ... ... ... ... ... ..G ... ... ... ... ... ... ... ... ...  [ 297]
TN94.616  ... ... ... ... ... ... ... ... ... ... ... ... ... ... ... ... ... ... ... ... ... ... ... ... ... ... ... ... ... ... ... ... ...  [ 297]
TN94.66   ... ... ... ... ... ... ... ... ... ... ... ... ... ... ... ... ... ..T ... ... ... ... ... ... ... ... ... ... ... ... ... ... ...  [ 297]
TN95.354  ... ... ... ... ... ... ... ... ... ... ... ... ... ... ... ... ... ..T ... ... ... ... ... ... ... ... ... ... ... ... ... ... ...  [ 297]
CAN97.83  ... ... ... ... ... ... ... ... ... ... ... ... ... ... ... ... ... ... ... ... ... ... ... ... ... ... ... ... ... ... ... ... ...  [ 297]
TN00.31   ... ... ... ... ... ... ... ... ... ... ... ... ... ... ... ... ... ... ... ... ... ... ... ... ... ... ... ... ... ... ... ... ...  [ 297]
TN00.314  ... ... ... ... ... ... ... ... ... ... ... ... ... ..A ... ... ... ... ... ... ... ... ... ... ... ... ... ... ... ... ... ... ...  [ 297]
TN00.43   ... ... ... ... ... ... ... ... ... ... ... ... ... ... ... ... ... ... ... ... ... ... ... ... ... ... ... ... ... ... ... ... ...  [ 297]
CAN00.16  ... ... ... ... ... ... ... ... ... ... ... ... ... ... ... ... ... ... ... ... ... ... ... ... ... ... ... ... ... ... ... ... ...  [ 297]
NL00.17   ... ... ... ... ... ... ... ... ... ... ... ... ... ... ... ... ... ... ... ... ... ... ... ... ... ... ... ... ... ... ... ... ...  [ 297]
JPS03.178 ... ... ... ... ... ... ... ... ... ... ... ... ... ... ... ... ... ..T ... ... ... ... ... ... ... ... ... ... ... .GA ... ... ...  [ 297]

TN82.79   CAA TCT AGA TTT GTT CTA GGA GCA ATA GCA CTC GGT GTT GCA ACA GCA GCT GCA GTT ACA GCA GGT GTT GCA ATT GCC AAA ACC ATC CGG CTT GAG AGT  [ 396]
TN84.127  ... ... ... ... ... ... ... ... ... ... ... ... ... ... ... ... ... ... ... ... ... ... ... ... ... ... ... ... ... ... ... ... ...  [ 396]
TN85.115  ... ... ... ... ... ... ... ... ... ... ... ... ... ... ... ... ... ... ... ... ... ... ... ... ... ... ... ... ... ... ... ... ...  [ 396]
TN85.57   ... ... ... ... ... ... ... ... ... ... ... ... ... ... ... ... ... ... ... ... ... ... ... ... ... ... ... ... ... ... ... ... ...  [ 396]
TN85.63   ... ... ... ... ... ... ... ... ... ... ... ... ... ... ... ... ... ... ... ... ... ... ... ... ... ... ... ... ... ... ... ... ...  [ 396]
TN86.314  ... ... ... ... ... ... ... ... ... ... ... ..C ... ... ... ... ... ... ... ... ... ... ... ... ... ... ... ... ... ... ... ... ..C  [ 396]
TN88.59   ... ... ... ... ... ... ... ... ... ... ... ... ... ... ... ... ... ... ... ... ... ... ... ... ... ... ... ... ... ... ... ... ...  [ 396]
JPY88.12  ... ... ... ... ... ... ... ... ... ... ... ... ... ..G ... ... ... ... ... ... ... ... ... ... ... ... ... ... ... ... ... ... ...  [ 396]
TN91.1292 ... ..C ... ... ... ... ... ... ... ... ... ... ... ... ... ... ... ... ... ... ... ... ... ... ... ... ... ... ... ... ... ... ...  [ 396]
TN92.313  ..G ... ... ... ... ... ... ... ... ... ... ... ... ... ... ... ... ... ... ... ... ... ... ... ... ... ... ... ... ... ... ... ...  [ 396]
TN92.250  ... ... ..G ... ... ... ... ... ... ... ..T ... ... ... ... ... ..C ... ... ... ... ... ... ... ... ... ... ... ... ... ... ... ...  [ 396]
TN92.435  ... ..C ... ... ... ... ... ... ... ... ... ... ... ... ... ... ... ... ... ... ... ... ... ... ... ... ... ... ... ... ... ... ...  [ 396]
TN94.49   ... ..C ... ... ... ... ... ... ... ... ... ... ... ... ... ... ... ... ... ... ... ... ... ... ... ... ... ... ... ... ... ... ...  [ 396]
TN94.616  ... ... ..G ... ... ... ... ... ... ... ... ... ... ... ... ... ... ... ... ... ... ... ... ... ... ... ... ... ... ... ... ... ...  [ 396]
TN94.66   ... ... ... ... ... ... ... ... ... ... ... ... ... ... ... ... ... ... ... ... ... ... ... ... ... ... ... ... ... ... ... ... ...  [ 396]
TN95.354  ... ... ... ... ... ... ... ... ... ... ... ... ... ... ... ... ... ... ... ... ... ... ... ... ... ... ... ... ... ... ... ... ...  [ 396]
CAN97.83  ... ... ..G ... ... ... ... ... ... ... ... ... ... ... ... ... ... ... ..C ... ... ... ... ... ... ... ... ... ... ... ... ... ...  [ 396]
TN00.31   ... ... ..G ... ... ... ... ... ... ... ... ... ... ... ... ... ... ... ... ... ... ... ... ... ... ... ... ... ... ... ... ... ...  [ 396]
TN00.314  ... ... ..G ... ... ... ... ... ... ... ... ... ... ... ... ... ... ... ... ... ... ... ... ... ... ... ... ... ... ... ... ... ...  [ 396]
TN00.43   ... ... ... ... ... ... ... ... ... ... ... ... ... ... ... ... ... ... ..C ... ... ... ... ... ... ... ... ... ... ... ..A ... ...  [ 396]
CAN00.16  ... ... ..G ... ... ... ... ... ... ... ... ... ... ... ... ... ... ... ..C ... ... ... ... ... ... ... ... ... ... ... ... ... ...  [ 396]
NL00.17   ... ... ..G ... ... ... ... ... ... ... ... ... ... ... ... ... ... ... ..C ... ... ... ... ... ... ... ... ... ... ... ... ... ...  [ 396]
JPS03.178 ... ... ... ... ... ... ... ... ... ... ... ... ... ..G ... ... ... ... ... ... ... ... ... ... ... ... ... ... ... ... ... ... ...  [ 396]

TN82.79   GAA GTC ACA GCA ATT AAG AAT GCC CTT AAA AAG ACC AAT GAA GCA GTA TCT ACA TTG GGG AAT GGA GTT CGA GTG TTA GCA ACT GCA GTG AGA GAG CTG  [ 495]
TN84.127  ... ... ... ... ... ... ... ... ... ... ... ... ... ... ... ... ... ... ... ... ... ... ... ... ... ..G ... ... ... ... ... ... ...  [ 495]
TN85.115  ... ... ... ... ... ..A ... ... ... ... ... ... ... ... ... ... ... ... ... ... ... ... ... ... ... ..G ... ... ... ... ... ... ...  [ 495]
TN85.57   ... ... ... ... ... ... ... ... ... ... ... ... ... ... ... ... ... ... ... ... ... ... ... ... ... ..G ... ... ... ... ... ... ...  [ 495]
TN85.63   ... ... ... ... ... ..A ... ... ... ... ... ... ... ... ... ... ... ... ... ... ... ... ... ... ... ..G ... ... ... ... ... ... ...  [ 495]
TN86.314  ... ... ... ... ..C ..A ... ... ... ... ... ... ... ... ... ... ..C ... C.. ... ... ... ... ... ... ..G ... ... ... ... ... ... ...  [ 495]
TN88.59   ... ... ... ... ... ... ... ... ... ... ... ... ... ... ... ... ... ... ... ... ... ... ... ... ... ..G ... ... ... ... ... ... ...  [ 495]
JPY88.12  ... ..T ... ... ... ... ... ... ... ... ... ..T ... ... ... ... ... ... ... ... ... ... ... ... ... ... ... ... ... ... ..G ..A ...  [ 495]
TN91.1292 ... ... ... ... ... ... ... ... ... ... ... ... ... ... ... ... ... ... ... ... ... ... ... ... ... ..G ... ... ... ... ... ... ...  [ 495]
TN92.313  ... ... ... ... ..C ... ... ... ... ... ... ... ... ... ... ... ... ... ... ... ... ... ... ... ... ..G ... ... ... ... ... ... ...  [ 495]
TN92.250  ... ... ... ... ... ... ... ... ..C ... ... ... ... ... ... ... ... ... ... ... ... ... ... ... ... ..G ... ... ... ... ... ... ...  [ 495]
TN92.435  ... ... ... ... ... ... ... ... ... ... ... ... ... ... ... ... ... ... ... ... ... ... ... ... ... ..G ... ... ... ... ... ... ...  [ 495]
TN94.49   ... ... ... ... ... ... ... ... ... ... ... ... ... ... ... ... ... ... ... ... ... ... ... ... ... ..G ... ... ... ..A ... ... ...  [ 495]
TN94.616  ... ... ... ... ... ... ... ... ..C ... ... ... ... ... ... ... ... ... ... ... ... ... ... ... ... ..G ... ... ... ... ... ... T..  [ 495]
TN94.66   ... ..T ... ... ... ... ... ... ... ... ... ... ... ... ... ..G ... ... ... ... ..C ... ... ... ... ... ... ... ... ... ... ..A ...  [ 495]
TN95.354  ... ..T ... ... ... ... ... ... ... ... ... ... ... ... ... ..G ... ... ... ... ... ... ... ... ... ... ... ... ... ... ... ..A ...  [ 495]
```

```
CAN97.83  ... ... ... ... ... ... ... ... ..C ... .C. ... ... ... ... ... ... ... ... ... ... ... ... ... ... ..G ... ... ... ... ... ... ...  [ 495]
TN00.31   ... ... ... ... ... ... ... ... ..C ... .C. ... ... ... ... ... ... ... ... ... ... ... ... ... ... ..G ... ... ... ... ... ... ...  [ 495]
TN00.314  ... ... ... ... ... ... ... ... ..C ... .C. ... ... ... ... ... ... ... ... ... ... ... ... ... ... ..G ... ... ... ... ... ... ...  [ 495]
TN00.43   ... ... ... ... ... ... ... ... ..C ... .C. ... ... ... ... ... ... ... ... ... ... ... ... ... ... ..G ... ... ... ... ... ... ...  [ 495]
CAN00.16  ... ... ... ... ... ... ... ... ..C ... .C. ... ... ... ... ... ... ... ... ... ... ... ... ... ... ..G ... ... ... ... ... ... ...  [ 495]
NL00.17   ... ... ... ... ... ... ... ... ..C ... .C. ... ... ... ... ... ... ... ... ... ... ... ... ... ... ..G ... ... ... ... ... ... ..A  [ 495]
JPS03.178 ... ..T ... ... ... ... ... ... ... ... ... ..T ... ... ... ... ... ... ... ... ... ... ... ... ... ... ... ... ... ... ..G ..A ...  [ 495]

TN82.79   AAA GAT TTT GTG AGC AAG AAT TTA ACT CGT GCA ATC AAC AAA AAC AAG TGC GAC ATT GAT GAC CTA AAA ATG GCC GTT AGC TTC AGT CAA TTC AAC AGA  [ 594]
TN84.127  ... ... ... ... ... ... ... ... ... ... ... ... ... ... ... ... ... ... ... ... ... ... ... ... ... ..C ... ... ... ... ... ... ..G  [ 594]
TN85.115  ... ... ... ... ... ... ... ... ... ... ... ... ... ... ... ... ... ... ... ... ... ... ... ... ... ... ... ... ... ... ... ... ...  [ 594]
TN85.57   ... ... ... ... ... ... ... ... ... ... ... ... ... ... ... ... ... ... ... ... ... ... ... ... ... ..C ... ... ... ... ... ... ...  [ 594]
TN85.63   ... ... ... ... ... ... ... ... ... ... ... ... ... ... ... ... ... ... ... ... ... ... ... ... ... ... ... ... ... ... ... ... ...  [ 594]
TN86.314  ... ... ... ... ... ... ... ... ... ... ... ... ... ... ... ... ... ... ... ... ... ... ... ... ... ... ... ... ... ... ... ... ...  [ 594]
TN88.59   ... ... ... ... ... ... ... ... ... ... ... ... ... ... ... ... ... ... ... ... ... ... ... ... ... ... ... ... ... ... ... ... ...  [ 594]
JPY88.12  ... ... ... ... ... ... ... ... ... ... ... ... ... ... ... ... ... ... ... ... ... ... ... ... ... ... ... ... ... ... ... ... ...  [ 594]
TN91.1292 ... ... ... ... ... ... ... ... ... ... ... ... ... ... ... ... ... ... ... ... ... ... ... ... ... ... ... ... ... ... ... ... ...  [ 594]
TN92.313  ... ... ... ... ... ... ... ... ... ... ... ... ... C.. ... ... ... ... ... ... ..T ... ... ... ... ... ... ... ... ... ... ... ...  [ 594]
TN92.250  ... ... ... ... ... ... ... ... ... ... ... ... ... ... ... ... ... ... ... ... ... ... ... ... ... ... ... ... ... ... ... ... ...  [ 594]
TN92.435  ... ... ... ... ... ... ... ... ... ... ... ... ... ... ... ... ... ... ... ... ... ... ... ... ... ... ... ... ... ... ... ... ...  [ 594]
TN94.49   ... ... ... ... ... ... ... ... ... ... ... ... ... ... ... ... ... ... ... ... ... ... ... ... ... ... ... ... ... ... ... ... ...  [ 594]
TN94.616  ... ... ... ... ... ..A ... ... ... ... ... ... ... ... ... ... ... ... ... ... ... ... ... ... ..T ... ... ... ... ... ... ... ...  [ 594]
TN94.66   ... ... ... ... ... ... ... ... ... ... ... ... ... ... ... ... ... ... ... ... ... ... ... ... ... ... ... ... ... ... ... ... ...  [ 594]
TN95.354  ... ... ... ... ... ... ... ... ... ... ... ... ... ... ... ... ... ... ... ... ... ... ... ... ... ... ... ... ... ... ... ... ...  [ 594]
CAN97.83  ... ..C ... ... ... ... ... ... ... ... ... ... ... ... ... ... ... ... ... ... ... ... ... ... ... ... ... ... ... ... ... ... ...  [ 594]
TN00.31   ... ... ... ... ... ... ... ... ... ... ... ... ... ... ... ... ... ... ... ... ... ... ... ... ... ... ... ... ... ... ... ... ...  [ 594]
TN00.314  ... ... ... ... ... ... ... ... ... ... ... ... ... ... ... ... ... ... ... ... ... ... ... ... ... ... ... ... ... ... ... ... ...  [ 594]
TN00.43   ... ... ... ... ... .G. ... ... ... ... ... ... ... ... ... ... ... ... ... ... ... ... ... ... ... ... ... ... ... ... ... ... ...  [ 594]
CAN00.16  ... ..C ... ... ... ... ... ... ... ... ... ... ... ... ... ... ... ... ... ... ... ... ... ... ... ... ... ... ... ... ... ... ...  [ 594]
NL00.17   ... ..C ... ... ... ... ... ... ... ... ... ... ... ... ... ... ... ... ... ... ... ... ... ... ..T ... ... ... ... ... ... ... ...  [ 594]
JPS03.178 ... ... ... ... ..T ... ... ... ... ... ... ... ... ... ... ... ... ... ... ... ... ... ... ... ..T ... ... ... ... ... ... ... ...  [ 594]

TN82.79   AGG TTT CTA AAT GTT GTG CGG CAA TTT TCA GAC AAT GCT GGA ATA ACA CCA GCA ATA TCT TTG GAC TTA ATG ACA GAT GCT GAA CTA GCC AGG GCC GTT  [ 693]
TN84.127  ... ... ... ... ... ..A ... ... ... ... ... ... ... ... ... ... ... ... ... ... ... ... ... ... ... ... ... ... ... ... ... ... ...  [ 693]
TN85.115  ... ... ... ... ... ..A ... ... ... ... ... ... ... ... ... ... ... ... ... ... ... ... ... ... ... ... ... ... ... ... ... ... ...  [ 693]
TN85.57   ... ... ... ... ... ..A ... ... ... ... ... ... ... ... ... ... ... ... ... ... ... ... ... ... ... ... ... ... ... ... ... ... ...  [ 693]
TN85.63   ... ... ... ... ... ..A ... ... ... ... ... ... ... ... ... ... ... ... ... ... ... ... ... ... ... ... ... ... ... ... ... ... ...  [ 693]
TN86.314  ... ... ... ... ... ..A ... ... ... ... ... ... ... ... ... ... ... ... ... ... ... ... ... ... ... ... ... ... ... ... ... ... ...  [ 693]
TN88.59   ... ... ... ... ... ... ... ..G ... ... ... ... ... ... ... ... ... ... ... ... ... ... ... ... ... ... ... ... ... ... ... ... ...  [ 693]
JPY88.12  ... ... ... ... ... ... ... ... ... ... ... ... ... ... ... ... ... ... ... ... ..A ... ... ... ... ... ... ... ... ... ... ... ...  [ 693]
TN91.1292 ... ... ... ... ... ... ... ... ... ... ... ... ... ... ... ... ... ... ... ... ... ... ... ... ... ... ... ... ... ... ... ..T ...  [ 693]
TN92.313  ... ... ... ... ... ... ... ... ... ... ... ... ... ... ... ... ... ... ... ... ... ... ... ... ... ... ... ... ... ... ... ... ...  [ 693]
TN92.250  ... ... ... ... ... ... ... ... ... ... ... ... ... ... ... ... ... ... ... ... ... ... ... ... ... ... ... ... ... ... ... ... ...  [ 693]
TN92.435  ... ... ... ... .Y. ... ... ... ... ... ... ... ... ... ... ... ... ... ... ... ... ... ... ... ... ... ... ... ... ... ... ..T ...  [ 693]
TN94.49   ... ... ... ... ... ... ... ... ... ... ... ... ... ... ... ... ... ... ... ... ... ... ... ... ... ... ... ... ... ... ... ... ..C  [ 693]
TN94.616  ... ... ... ... ... ... ... ... ... ... ... ... ... ... ... ... ... ... ... ... ... ... ... ... ... ... ... ... ... ... ... ... ...  [ 693]
TN94.66   ... ... ... ... ... ... ... ... ... ... ... ... ... ... ... ... ... ... ... ... ..A ... ... ... ... ... ... ... ..T ... ... ... ...  [ 693]
TN95.354  ... ... ... ... ... ... ... ... ... ... ... ... ... ... ... ... ... ... ... ... ..A ... ... ... ... ... ... ... ... ... ... ... ...  [ 693]
CAN97.83  ... ... ... ... ... ... ... ... ... ... ... ... ... ... ... ... ... ... ... ... ... ... ... ... ... ... ... ... ... ... ... ... ...  [ 693]
TN00.31   ... ... ... ... ... ... ... ... ... ... ... ... ... ... ... ... ... ... ... ... ... ... ... ... ... ... ... ... ... ... ... ..T ...  [ 693]
TN00.314  ... ... ... ... ... ... ... ... ... ... ... ... ... ... ... ... ... ... ... ... ... ... ... ... ... ... ... ... ... ... ... ... ...  [ 693]
TN00.43   ... ... ... ... ... ... ... ... ... ... ... ... ... ... ... ... ... ... ... ... ... ... ... ... ... ... ... ... ... ... ... ... ...  [ 693]
CAN00.16  ... ... ... ... ... ... ... ... ... ... ... ... ... ... ... ... ... ... ... ... ... ... ... ... ... ... ... ... ... ... ... ... ...  [ 693]
NL00.17   ... ... ... ... ... ... ... ... ... ... ... ... ... ... ... ... ... ... ... ... ... ... ... ... ... ... ... ... ... ... ... ... ...  [ 693]
JPS03.178 ... ... ... ... ... ... ... ... ... ... ... ... ... ... ... ... ... ... ... ... ..A ... ... ... ... ... ... ... ... ... ... ... ...  [ 693]
```

```
TN82.79   TCC AAC ATG CCG ACA TCT GCA GGA CAA ATA AAA TTG ATG TTG GAG AAC CGT GCA ATG GTG CGA AGA AAG GGG TTC GGA ATC CTG ATA GGG GTC TAC GGG  [ 792]
TN84.127  ... ... ... ..A ... ... ... ... ... ... ... ... ... ... ... ... ... ... ... ... ... ... ... ... ... ... ... ... ... ... ... ... ...  [ 792]
TN85.115  ... ... ... ..A ... ... ... ... ... ... ... ... ... ... ... ... ... ... ... ... ... ... ... ... ... ... ... ... ... ... ... ... ...  [ 792]
TN85.57   ... ... ... ..A ... ... ... ... ... ... ... ... ... ... ... ... ... ... ... ... ... ... ... ... ... ... ... ... ... ... ... ... ...  [ 792]
TN85.63   ... ... ... ..A ... ... ... ... ... ... ... ... ... ... ... ... ... ... ... ... ... ... ... ... ... ... ... ... ... ... ... ... ...  [ 792]
TN86.314  ... ... ... ..A ... ... ... ... ... ... ... ... ... ... ... ... ... ... ... ... ... ... ... ... ... ... ... ... ... ... ... ... ...  [ 792]
TN88.59   ... ... ... ... ... ... ... ... ... ... ... ... ... ... ... ... ..C ... ... ... ... ... ... ... ..T ... ... ... ... ... ... ... ...  [ 792]
JPY88.12  ... ... ... ... ... ... ... ... ... ... ... ... ... ... ..A ... ... ... ... ... ... ..G ... ... ... ... ... ... ... ... ... ... ...  [ 792]
TN91.1292 ... ... ... ... ... ... ... ... ... ... ... ... ... ... ... ... ... ... ... ... ... ... ... ... ... ... ... ... ... ... ... ... ...  [ 792]
TN92.313  ... ... ... ... ... ... ... ... ... ... ... ... ... ... ... ... ... ... ... ... ... ... ... ... ... ... ... ... ... ... ... ... ...  [ 792]
TN92.250  ..T ... ... ... ... ... ... ... ... ... ... ... ... ... ... ... ... ... ... ... ... ... ... ... ... ... ... ... ... ... ... ... ...  [ 792]
TN92.435  ... ... ... ... ... ... ... ... ... ... ... ... ... ... ... ... ... ... ... ... ... ... ... ..A ... ... ... ... ... ... ... ... ...  [ 792]
TN94.49   ... ... ... ... ... ... ... ... ... ... ... ... ... ... ... ... ... ... ... ... ... ... ... ... ..T ... ... ... ... ... ... ... ...  [ 792]
TN94.616  ..T ... ... ... ... ... ... ... ... ... ... ... ... ... ... ... ... ... ... ... ... ... ... ... ... ... ... ... ... ... ... ... ...  [ 792]
TN94.66   ... ... ... ... ... ... ... ... ... ... ... ... ... ... ..A ... ... ... ... ... ... ..G ... ... ... ... ... T.. ... ... ... ... ...  [ 792]
TN95.354  ... ... ... ..A ... ... ... ... ... ... ... ... ... ... ..A ... ... ... ... ... ... ..G ... ... ... ... ... ... ... ... ... ... ...  [ 792]
CAN97.83  ..T ... ... ... ... ... ... ... ... ... ... ... ... ... ... ... ... ..G ... ... ... ... ... ... ... ... ... ... ... ... ... ... ...  [ 792]
TN00.31   ..T ... ... ... ... ... ... ... ... ... ... ... ... ... ... ... ... ... ... ... ... ... ... ... ... ... ... ... ... ... ... ... ...  [ 792]
TN00.314  ..T ... ... ... ... ... ... ... ... ... ... ... ... ... ... ... ... ... ... ... ... ... ... ... ... ... ... ... ... ... ... ... ...  [ 792]
TN00.43   ..T ... ... ... ... ... ... ... ... ... ..G ... ... ... ... ... ... ..G ... ... ... ... ... ... ... ... ... ... ... ... ... ... ...  [ 792]
CAN00.16  ..T ... ... ... ... ... ... ... ... ... ... ... ... ... ... ... ..C ..G ... ... ... ... ... ... ... ... ... ... ... ... ... ... ...  [ 792]
NL00.17   ..T ... ... ... ... ... ... ... ... ... ... ... ... ... ... ... ... ..G ... ... ... ... ... ... ... ... ... ... ... ... ... ... ...  [ 792]
JPS03.178 ... ... ... ... ... ... ... ... ... ... ... ... ... ... ..A ..T ... ... ... ... ... ..G ... ... ... ... ... ... ... ... ... ..T ...  [ 792]

TN82.79   AGC TCC GTA ATT TAC ATG GTG CAG CTG CCA ATC TTT GGC GTT ATA GAC ACG CCT TGC TGG ATA GTA AAA GCA GCC CCC TCT TGT TCC GAA AAA AAG GGA  [ 891]
TN84.127  ... ..T ... ... ... ... ... ... ... ... ... ... ... ... ... ... ... ... ... ... ... ... ... ... ... ... ..C ... ... ... ... ... ...  [ 891]
TN85.115  ... ... ... ... ... ... ... ... ... ... ... ... ... ... ... ... ..A ... ... ... ... ... ... ... ..T ... ... ... ... ... ... ... ...  [ 891]
TN85.57   ... ..T ... ... ... ... ... ... ... ... ... ... ... ... ... ... ... ... ... ... ... ... ... ... ... ... ... ... ... ... ... ... ...  [ 891]
TN85.63   ... ... ... ... ... ... ... ... ... ... ... ... ... ... ... ... ..A ... ... ... ... ... ... ... ..T ... ... ... ... ... ... .G. ...  [ 891]
TN86.314  ... ... ... ... ... ... ... ... ... ... ... ... ... ... ... ... ..A ... ... ... ... ... ... ... ..T ... ... ... ... ... ... .G. ...  [ 891]
TN88.59   ... ... ... ... ... ... ... ..A ... ... ... ... ... ... ... ... ... ... ... ... ... ... ... ... ... ... ... ... ... ... ... ... ...  [ 891]
JPY88.12  ... ... ... ..C ... ... ... ... ... ... ... ... ... ... ... ... ... ... ... ... ... ... ... ... ... ... ... ... ... ... ... ... ...  [ 891]
TN91.1292 ... ... ... ... ... ... ... ... ... ... ... ... ... ..C ... ... ... ... ... ... ... ... ... ... ... ... ... ... ... ... ... ... ...  [ 891]
TN92.313  ... ... ... ... ... ... ... ... ... ... ... ... ... ... ... ... ... ... ... ... ... ... ... ... ... ... ... ... ... ... ... ... ...  [ 891]
TN92.250  ... ... ... ... ... ... ... ... ... ... ... ... ... ... ... ... ... ... ... ... ... ... ... ... ... ... ... ... ... ... ... ... ...  [ 891]
TN92.435  ... ... ... ... ..T ... ... ... ... ... ... ... ... ..C ... ... ... ... ... ... ... ... ... ... ... ... ... ... ... ... ... ... ...  [ 891]
TN94.49   ... ... ... ... ... ... ... ... ... ... ... ... ... ..C ... ... ... ... ... ... ... ... ... ... ... ... ... ... ... ... ... ... ...  [ 891]
TN94.616  ... ... ... ... ... ... ... ... ... ... ... ... ... ... ... ... ... ... ... ... ... ... ... ... ... ... ... ... ... ... ... ... ...  [ 891]
TN94.66   ... ... ... ... ... ... ... ... ... ... ... ... ... ... ... ... ... ... ... ... ... ... ... ... ... ... ... ... ... ... ... ... ...  [ 891]
TN95.354  ... ... ... ... ... ... ... ... ... ... ... ... ... ... ... ... ... ... ... ... ... ... ... ... ... ... ... ... ... ... ... ... ...  [ 891]
CAN97.83  ... ... ... ... ... ... ... ... ... ... ... ... ... ... ... ... ... ... ... ... ... ... ... ... ... ..T ... ... ... .G. ... ... ...  [ 891]
TN00.31   ... ... ... ... ... ... ... ... ... ... ... ... ... ... ... ... ... ... ... ... ... ... ... ... ... ... ... ... ... ... ... ... ...  [ 891]
TN00.314  ... ... ... ... ... ... ... ... ... ... ... ... ... ... ... ... ... ... ... ... ... ... ... ... ... ... ... ... ... ... ... ... ...  [ 891]
TN00.43   ... ... ... ... ... ... ... ... ... ... ... ... ... ... ... ... ... ... ... ... ... ... ... ... ... ... ... ... ... ... ... ... ...  [ 891]
CAN00.16  ... ..T ... ... ... ... ... ... ... ... ... ... ... ... ... ... ... ... ... ... ... ... ... ... ... ..T ... ... ... ... ... ... ...  [ 891]
NL00.17   ... ... ... ... ... .C. ... ... ... ... ... ... ... ... ... ... ... ... ... ... ... ... ... ... ... ..T ... ... ... ... ... ... ...  [ 891]
JPS03.178 ... ... ... ..C ... ... ... ... ... ... ... ... ... ... ... ... ... ... ... ... ... ... ... ... ... ... ... ..G ... ... ... ... ...  [ 891]

TN82.79   AAC TAT GCT TGC CTT TTA AGA GAA GAT CAA GGG TGG TAT TGT CAG AAT GCA GGG TCA ACT GTT TAC TAC CCA AAT GAG AAA GAC TGT GAA ACA AGA GGA  [ 990]
TN84.127  ... ... ... ... ..C ... ... ... ..C ... ... ... ... ... ... ... ... ... ... ... ... ... ... ... ... ... ... ... ... ... ... ... ...  [ 990]
TN85.115  ... ... ... ... ..C ... ... ... ... ... ... ... ... ... ... ... ... ... ... ... ... ... ... ... ... ... ... ... ... ... ... ... ...  [ 990]
TN85.57   ... ... ... ... ..C ... ... ... ..C ... ... ... ... ... ... ... ... ... ... ... ... ... ... ... ... ... ... ... ... ... ... ... ...  [ 990]
TN85.63   ... ... ... ... ..C ... ... ... ... ... ... ... ... ... ... ... ... ... ... ... ... ... ... ... ... ... ... ... ... ... ... ... ...  [ 990]
TN86.314  ... ... ... ... ..C ... ... ... ... ... ... ... ... ... ... ... ... ... ... ... ... ... ... ... ... ... ... ... ... ... ... ... ...  [ 990]
TN88.59   ... ... ... ... ..C ... ... ... ..C ... ... ... ... ... ... ... ... ... ... ... ... ... ... ... ... ... ... ... ... ... ... ... ...  [ 990]
JPY88.12  ... ... ... ... ..C ... ... ... ..C ... ... ... ... ... ... ... ... ... ... ... ... ... ... ... ... ... ..G ... ... ... ... ... ...  [ 990]
```

```
TN91.1292 ... ... ... ... ... ... ... ... ... ... ... ... ... ... ... ... ... ... ... ... ... ... ... ... ... ... ... ... ... ... ... ... ...  [ 990]
TN92.313  ... ... ... ... ..C ... ... ... ..C ... ... ... ... ... ... ... ... ... ... ... ... ... ... ... ... ... ... ... ... ... ... ... ...  [ 990]
TN92.250  ... ... ... ... ..C ... ... ... ..C ... ... ... ... ... ... ... ... ... ... ... ... ... ... ... ... ... ... ... ... ... ... ... ...  [ 990]
TN92.435  .G. ... ... ... ... ... ... ... ... ... ... ... ... ... ... ... ... ... ... ... ... ... ... ... ... ... ... ... ... ... ... ... ...  [ 990]
TN94.49   ... ... ... ... ... ... ... ... ... ... ... ... ... ... ... ... ... ... ... ... ... ... ... ... ... ... ... ... ... ... ... ... ...  [ 990]
TN94.616  ... ... ... ... ..C ... ..G ... ..C ... ... ... ... ... ... ... ... ... ... ... ... ... ... ... ... ... ... ... ... ... ... ... ..G  [ 990]
TN94.66   ... ... ... ... ..C ... ... ... ..C ... ... ... ... ... ... ... ... ... ... ... ... ... ... ... ... ... ..G ... ... ... ... ... ...  [ 990]
TN95.354  ... ... ... ... ..C C.. ... ... ..C ... ... ... ... ... ... ... ... ... ... ... ... ... ... ... ... ... ..G ... ... ... ... ... ...  [ 990]
CAN97.83  ... ... ... ... ..C ... ... ... ..C ... ... ... ... ... ... ... ... ... ... ... ... ... ... ... ... ... ... ... ... ... ... ... ...  [ 990]
TN00.31   ... ... ... ... ..C ... ... ... ..C ... ... ... ... ... ... ... ... ... ... ... ... ... ... ... ... ... ... ... ... ... ... ... ...  [ 990]
TN00.314  ... ... ... ... ..C ... ... ... ..C ... ... ... ... ... ... ... ... ... ... ... ... ... ... ... ... ... ... ... ... ... ... ... ...  [ 990]
TN00.43   ... ... ... ..T ..C ... ... ... ..C ... ... ... ... ... ... ... ... ... ... ... ... ... ... ... ... ... ... ... ... ... ... ... ...  [ 990]
CAN00.16  ... ... ... ... ..C ... ... ... ..C ... ... ... ... ... ... ... ... ... ... ... ... ... ... ... ... ... ... ... ... ... ... ... ...  [ 990]
NL00.17   ... ... ... ... ..C ... ... ... ..C ... ... ... ... ... ... ... ... ... ... ... ... ... ... ... ... ... ... ... ... ... ... ... ...  [ 990]
JPS03.178 ... ... ... ... ..C ... ... ... ..C ... ... ... A.. ... ... ... ... ... ... ... ... ... ... ... ... ... ..G ... ... ... ... ... ...  [ 990]

TN82.79   GAC CAT GTC TTT TGC GAC ACA GCA GCA GGA ATT AAT GTT GCT GAG CAA TCA AAA GAG TGC AAC ATC AAC ATA TCC ACT ACA AAT TAC CCA TGC AAA GTC  [1089]
TN84.127  ... ... ... ..C ... ... ... ... ... ... ..C ... ... ... ... ... ... ..G ... ... ... ... ... ... ... ... ..C ... ... ... ... ... ...  [1089]
TN85.115  ... ... ... ... ... ... ... ... ... ... ... ... ... ... ... ... ... ..G ... ... ... ... ... ... ... ... ... ... ... ... ... ... ...  [1089]
TN85.57   ... ... ... ..C ... ... ... ... ... ... ..C ... ... ... ... ... ... ..G ... ... ... ... ... ... ... ... ..C ... ... ... ... ... ...  [1089]
TN85.63   ... ... ... ... ... ... ... ... ... ... ... ... ... ... ... ... ... ..G ... ... ... ... ... ... ... ... ... ... ... ... ... ... ...  [1089]
TN86.314  ... ... ... ... ... ... ... ... ... ... ... ... ... ... ... ... ... ..G ... ... ... ... ... ... ... ... ... ... ... ... ... ... ...  [1089]
TN88.59   ... ... ... ... ... ... ... ... ... ... ... ... ... ... ... ... ... ... ... ... ... ... ... ... ... ... ... ... ... ... ... ..G ...  [1089]
JPY88.12  ... ... ... ... ... ... ... ... ... ... ... ... ... ... ... ... ... ..G ... ... ... ... ... ... ... ..C ... ... ..T ..G ... ... ...  [1089]
TN91.1292 ... ... ... ... ... ... ... ... ... ... ... ... ... ... ... ... ... ... ... ... ... ... ... ... ... ... ... ... ... ... ... ... ...  [1089]
TN92.313  ... ... ... ... ... ... ... ... ... ... ... ... ... ... ... ... ... ... ... ... ... ... ... ... ... ... ... ... ... ... ... ... ...  [1089]
TN92.250  ... ... ... ... ... ... ... ... ... ... ... ... ... ... ... ... ... ..G ... ... ... ... ... ... ... ... ... ... ... ... ... ... ...  [1089]
TN92.435  ... ... ... ... ... ... ... ... ... ... ... ... ... ... ... ... ... ... ... ... ... ... ... ... ... ... ... ... ... ... ... ... ...  [1089]
TN94.49   ... ... ... ... ... ... ... ... ... ... ... ... ... ... ... ... ... ... ... ... ..T ... ... ... ... ... ... ... ... ... ... ... ...  [1089]
TN94.616  ... ... ... ... ... ... ... ... ... ... ... ..C ... ... ... ... ... ... ... ... ... ... ... ... ... ... ... ... ... ... ... ... ...  [1089]
TN94.66   ... ... ... ... ... ... ... ... ... ... ... ... ... ... ... ... ... ..G ... ... ... ... ... ... ... ... ... ... ... ... ... ... ...  [1089]
TN95.354  ... ... ... ... ... ... ... ... ... ... ... ... ... ... ... ... ... ..G ... ... ... ... ... ... ... ..C ... ... ... ... ... ... ...  [1089]
CAN97.83  ... ... ... ... ... ... ... ... ..G ... ... ... ... ... ... ... ... ..G ... ... ... ... ... ... ... ... ... ... ... ... ... ... ...  [1089]
TN00.31   ... ... ... ... ... ... ... ... ... ... ... ... ... ... ... ... ... ..G ... ... ... ... ... ... ... ... ... ... ... ... ... ... ...  [1089]
TN00.314  ... ... ... ... ... ... ... ... ... ... ... ... ... ... ... ... ... ..G ... ... ... ... ... ... ... ... ... ... ... ... ... ... ...  [1089]
TN00.43   ... ... ... ... ... ... ... ... ... ... ... ... ... ... ... ... ... ..G ... ... ... ... ... ... ... ... ... ... ... ... ... ... ...  [1089]
CAN00.16  ... ... ... ... ... ... ... ... ... ... ... ... ... ... ... ... ... ..G ... ... ... ... ... ... ... ... ... ... ... ... ... ... ...  [1089]
NL00.17   ... ... ... ... ... ... ... ... ... ... ... ... ... ... ... ... ... ..G ... ... ... ... ... ... ... ... ... ... ... ... ... ... ...  [1089]
JPS03.178 ... ... ... ... ... ... ... ... ... ... ... ... ... ... ... ... ... ..G ... ... ... ... ... ... ... ..C ... ... ..T ..G ... ... ...  [1089]

TN82.79   AGC ACA GGA AGA CAT CCT ATC AGT ATG GTT GCA CTG TCC CCT CTT GGG GCT CTG GTT GCT TGC TAC AAA GGA GTA AGC TGT TCC ATT GGC AGC AAT AGA  [1188]
TN84.127  ... ... ... ... ... ... ... ... ... ... ... ... ... ... ... ... ... ... ... ... ... ... ..G ... ... ... ... ... ... ... ... ... ...  [1188]
TN85.115  ... ... ... ... ... ... ... ... ... ... ... ... ... ... ... ... ... ... ... ... ... ... ... ... ... ... ... ... ... ... ... ... ...  [1188]
TN85.57   ... ... ... ... ... ... ... ... ... ... ... ... ... ... ... ... ... ... ... ... ... ... ..G ... ... ... ... ... ... ... ... ... ...  [1188]
TN85.63   ... ... ... ... A.. ... ... ... ... ... ... ... ... ... ... ... ... ..A ... ... ... ... ... ... ... ... ... ... ... ... ... ... ...  [1188]
TN86.314  ... ... ... ... ... ... ... ... ... ... ... ... ... ... ... ... ... ... ... ... ... ... ... ... ... ... ... ... ... ... ... ... ...  [1188]
TN88.59   ... ... ... ... ... ... ... ... ... ... ... ... ... ... ... ..A ... ... ... ... ... ... ... ... ... ... ... ... ... ... ... ... ...  [1188]
JPY88.12  ... ... ... ..G ... ... ... ... ... ... ... ... ... ... ..C ... ... ... ... ..C ..T ... ... ... ... ... ... ... ... ... ... ... ...  [1188]
TN91.1292 ... ... ... ... ... ... ... ... ... ... ... ... ... ... ... ... ... ... ... ... ... ... ... ... ... ... ... ... ... ... ... ... ...  [1188]
TN92.313  ... ... ... ... ... ... ... ... ... ... ... ... ... ... ... ... ... ... ... ... ... ... ... ..G ... ... ... ... ... ... ... ... ...  [1188]
TN92.250  ... ... ... ... ... ... ... ... ... ... ... ... ..T ... ... ... ... ... ... ... ... ... ... ... ... ... ... ... ... ... ... ..C ...  [1188]
TN92.435  ... ... ... ... ... ... ... ... ... ... ... ... ... ... ... ... ... ... ... ... ... ... ... ... ... ... ... ... ... ... ... ... ...  [1188]
TN94.49   ... ... ... ... ... ... ... ... ... ... ... ... ..T ... ... ... ... ..A ... ... ... ... ... ... ... ... ... ... ... ... ... ... ...  [1188]
TN94.616  ... ... ... ... ... ... ... ... ... ... ... ... ..T ... ... ... ... ... ... ... ... ... ... ... ... ... ... ..T ... ... ... ..C ...  [1188]
TN94.66   ... ... ... ..G ... ... ... ... ... ... ... ... ... ... ... ... ... ... ... ... ..T ... ... ... ... ... ... ... ... ... ... ... ...  [1188]
TN95.354  ... ... ... ..G ... ... ... ... ... ... ... ... ... ... ... ... ... ... ... ... ..T ... ... ... ... ... ... ..T ... ... ... ... ...  [1188]
```

```
CAN97.83  ... ... ... ... ... ... ... ... ... ... ... ... ..T ... ... ... ... ... ... ... ... ... ... ... ... ... ... ... ... ... ... ..C ...  [1188]
TN00.31   ... ... ... ... ... ... ... ... ... ... ... ... ..T ... ... ... ... ... ... ... ... ... ... ... ... ... ... ... ... ... ... ..C ...  [1188]
TN00.314  ... ... ... ... ... ... ... ... ... ... ... T.. ..T ... ... ... ... ... ... ... ... ... ... ... ... ... ... ... ... ... ... ..C ...  [1188]
TN00.43   ... ... ... ... ... ... ... ... ... ... ... ... ..T ... ... ..A ... ..A ... ... ... ... ... ... ... ... ... ... ... ... ... ..C ...  [1188]
CAN00.16  ... ... ... ... ... ... ... ... ... ... ... ... ..T ... ... ... ... ... ... ... ... ... ... ... ... ... ... ... ... ... ... ..C ...  [1188]
NL00.17   ... ... ... ... ... ... ... ... ... ... ... ... ..T ... ... ... ... ... ... ... ... ... ... ... ... ... ... ... ... ... ... ..C ...  [1188]
JPS03.178 ... ... ... ..G ... ... ... ... ... ... ... ... ... ... ..C ... ... ... ... ..C ..T ... ... ... ... ... ... ... ... ... ... ... ...  [1188]

TN82.79   GTA GGG ATC ATC AAG CAG CTG AAC AAA GGT TGC TCT TAT ATA ACC AAC CAA GAT GCA GAC ACA GTG ACA ATA GAC AAC ACT GTA TAT CAG CTA AGC AAA  [1287]
TN84.127  ... ... ... ... ... ... ... ... ... ... ... ... ... ... ... ... ... ... ... ... ... ... ... ... ... ... ... ... ... ... ... ... ...  [1287]
TN85.115  ... ... ... ... ... ... ... ... ... ... ... ... ... ... ... ... ... ... ... ... ... ... ... ... ... ... ... ... ... ... ... ... ...  [1287]
TN85.57   ... ... ... ... ... ... ... ... ... ... ... ... ... ... ... ... ... ... ... ... ... ... ... ... ... ... ... ... ... ... ... ... ...  [1287]
TN85.63   ... ... ... ... ... ... ... ... ... ... ... ... ... ... ... ... ... ... ... ... ... ... ... ... ... ... ... ... ... ... ... ... ...  [1287]
TN86.314  ... ... ... ... ... ... ... .G. ... ... ... ... ... ... ... ... ... ... ... ... ... ... ... ... ... ... ... ... ... ... ... ... ...  [1287]
TN88.59   ... ... ... ... ... ... ... ... ... ... ... ... ... ... ... ... ... ... ... ... ... ... ... ... ... ... ... ... ... ... ... ... ...  [1287]
JPY88.12  ... ... ... ... ... ... ... ... ... ... ... ... ... ... ... ... ... ... ... ... ... ... ... ... ..T ... ... ... ... ... ... ... ...  [1287]
TN91.1292 ... ... ... ... ... ... ... ... ... ... ... ..C ... ... ... ... ... ... ... ... ... ... ... ... ... ... ..A ... ... ... ... ... ...  [1287]
TN92.313  ... ... ... ... ... ... ... ... ... ..C ... ... ... ... ... ... ... ... ... ..T ... ... ... ... ... ... ... ... ... ... ... ... ...  [1287]
TN92.250  ... ... ... ... ... ... ... ... ... ... ... ..C ... ... ... ... ... ... ... ... ... ... ... ... ... ... ... ... ... ... ... ... ...  [1287]
TN92.435  ... ... ... ... ... ... ... ... ... ... ... ..C ... ... ... ... ... ... ... ... ... ... ... ... ... ... ..A ... ... ... ... ... ...  [1287]
TN94.49   ... ... ... ... ... ... ... ... ... ... ... ..C ... ... ... ... ... ... ... ... ... ... ... ... ... ... ... ... ... ... ... ... ...  [1287]
TN94.616  ... ... ... ... ... ... ... ... ... ... ... ..C ... ... ... ... ... ... ... ... ... ... ... ... ... ... ... ... ... ... ... ... ...  [1287]
TN94.66   ... ... ... ... ... ... ... .G. ... ... ... ... ... ... ... ... ... ... ... ... ... ... ... ... ... ... ... ... ... ..A ... ... ...  [1287]
TN95.354  ... ... ... ... ... ... ... ... ... ... ... ... ... ... ... ... ... ... ... ... ... ... ... ... ... ... ... ..G ... ... ... ... ...  [1287]
CAN97.83  ... ... ... ... ... ... ... ... ..G ... ... ..C ... ... ... ... ... ... ... ... ... ... ... ... ... ... ... ... ... ... ... ... ...  [1287]
TN00.31   ... ... ... ... ... ... ... ... ... ... ... ..C ... ... ... ... ... ... ... ... ... ... ... ... ... ... ... ... ... ... ... ... ...  [1287]
TN00.314  ... ... ... ... ... ... ... ... ... ... ... ..C ... ... ... ... ... ... ... ... ... ... ... ... ... ... ... ... ... ... ... ... ...  [1287]
TN00.43   ... ... ... ... ... ... ... ... ... ... ... ..C ... ... ... ... ... ... ... ... ... ... ... ... ..T ... ... ... ... ... ... ... ...  [1287]
CAN00.16  ... ... ... ... ... ... ... ... ... ... ... ..C ... ... ... ... ... ... ... ... ... ... ... ... ... ... ... ... ... ... ... ... ...  [1287]
NL00.17   ... ... ... ... ... ... ... ... ... ... ... ..C ... ... ... ... ... ... ... ... ... ... ... ... ... ... ... ... ... ... ... ... ...  [1287]
JPS03.178 ... ... ... ... ... ... ... ... ... ... ... ... ... ... ... ... ... ... ... ... ... ... ... ... ..T ... ... ... ... ... ... ... ...  [1287]

TN82.79   GTT GAG GGT GAA CAG CAT GTT ATA AAA GGC AGA CCA GTG TCA AGC AGC TTT GAT CCA GTC AAG TTT CCT GAA GAT CAA TTC AAT GTT GCA CTT GAC CAA  [1386]
TN84.127  ... ... ... ... ... ... ... ... ... ... ... ... ... ... ... ... ..C ... ... ... ... ... ... ... ... ... ... ... ... ... ... ... ...  [1386]
TN85.115  ... ... ... ... ... ... ... ... ... ... ... ... ... ... ..T ... ... ... ... ..T ... ... ... ... ... ... ... ... ... ... ... ... ...  [1386]
TN85.57   ... ... ... ... ... ... ... ... ... ... ... ... ... ... ... ... ..C ... ... ... ... ... ... ... ... ... ... ... ... ... ... ... ...  [1386]
TN85.63   ... ... ... ... ... ... ... ... ... ... ... ... ... ... ..T ... ... ... ... ..T ... ... ... ... ... ... ... ... ... ... ... ... ...  [1386]
TN86.314  ... ... ... ... ... ... ... ... ... ... ... ... ... ... ..T ... ... ... ... ..T ... ... ... ... ... ... ... ... ... ... ... ... ...  [1386]
TN88.59   ... ... ... ... ... ... ... ... ... ... ... ... ... ... ... ... ... ... ... ... ... ... ... ... ... ... ... ... ... ... ... ... ...  [1386]
JPY88.12  ... ..A ... ... ... ... ... ... ... ... ... ... ... ... ... ... ... ... ... ... ... ... ... ... ... ... ... ... ... ... ... ... ...  [1386]
TN91.1292 ... ... ... ... ... ... ... ... ... ... ... ... ... ... ... ... ... ... ... ... ... ... ... ... ... ... ... ... ... ... ... ... ...  [1386]
TN92.313  ... ..A ... ... ... ... ... ... ... ... ... ... ... ... ... ... ... ... ... ... ... ... ... ... ... ... ... ... ... ... ... ... ...  [1386]
TN92.250  ... ... ... ... ... ... ... ... ... ... ... ... ... ... ... ... ... ... ... A.. ... ... ... ... ... ... ... ... ... ... ... ... ...  [1386]
TN92.435  ... ... ... ... ... ... ... ... ... ... ... ... ... ... ... ... ... ... ... ... ... ... ... ... ... ... ... ... ... ... ... ... ...  [1386]
TN94.49   ... ... ... ... ... ... ... ... ... ... ... ... ... ... ... ... ... ... ... ... ... ... ... ... ... ... ... ... ... ... ... ... ...  [1386]
TN94.616  ... ... ... ... ... ... ... ... ... ... ... ... ... ... ... ... ... ... ... A.. ... ... ... ... ... ... ... ... ... ... ... ... ...  [1386]
TN94.66   ... ... ... ... ... ... ... ... ... ... ... ... ... ... ... ... ... ... ... ... ... ... ... ... ... ... ... ... ... ... ... ... ...  [1386]
TN95.354  ... ... ... ... ... ... ... ... ... ... ... ... ... ... ... ... ... ... ... ... ... ... ... ... ... ... ... ... ... ... ... ... ...  [1386]
CAN97.83  ... ... ... ... ... ... ... ... ... ... ... ... ... ... ... ... ... ... ... A.. ... ... ... ... ... ... ... ... ... ... ... ... ...  [1386]
TN00.31   ... ... ... ... ... ... ... ... ... ... ... ... ... ... ... ... ... ... ... A.. ... ... ... ... ... ... ... ..C ... ... ... ... ...  [1386]
TN00.314  ... ... ... ... ... ..C ... ... ... ... ... ... ... ... ... ... ... ... ... A.. ... ... ... ... ... ... ... ... ... ... ... ... ...  [1386]
TN00.43   ... ... ... ... ... ... ... ... ... ... ... ... ... ... ... ... ... ... ... A.. ... ..C ... ... ... ... ... ... ... ... ... ... ..G  [1386]
CAN00.16  ... ... ... ... ... ... ... ... ... ... ... ... ... ... ... ... ... ... ... A.. ... ... ... ... ... ... ... ... ... ... ... ... ...  [1386]
NL00.17   ... ... ... ... ... ... ... ... ... ... ... ... ... ... ... ... ... ... ... A.. ... ... ... ... ... ... ... ... ... ... ... ... ...  [1386]
JPS03.178 ... ... ... ... ... ... ... ... ... ... ... ... ... ... ... ... ... ... ... ... ... ... ... ... ... ... ... ... ... ... ... ... ...  [1386]
```

```
TN82.79   GTT TTT GAG AAC ATT GAA AAC AGC CAG GCC TTG GTA GAT CAA TCA AAC AGA ATC CTA AGC AGT GCA GAG AAA GGG AAC ACT GGC TTC ATC ATT GTA ATA  [1485]
TN84.127  ... ... ... ... ... ... ... ... ... ... ... ... ... ... ... ... ... ... ... ... ..C ... ... ... ... ... ... ... ... ... ... ... ...  [1485]
TN85.115  ... ... ... ... ... ... ... ... ... ... ... ... ... ... ... ... ... ... ... ... ... ... ... ... ... ... ... ... ... ... ... ... ...  [1485]
TN85.57   ... ... ... ... ... ... ... ... ... ... ... ... ... ... ... ... ... ... ... ... ..C ... ... ... ... ... ... ... ... G.. ... ... ...  [1485]
TN85.63   ... ... ... ... ... ... ... ... ... ... ... ... ... ... ... ... ... ... ... ... ... ... ... ... ... ... ... ... ... ... ... ... ...  [1485]
TN86.314  ... ... ... ... ... ... ... ... ... ... ... ... ... ... ... ... ... ... ... ... ... ... ... ... ... ... ... ... ... ... ... ... ...  [1485]
TN88.59   ... ... ... ... ... ... ... ... ... ... ... ... ... ... ... ... ... ... ... ... ... ... ... ... ... ... ... ... ... ... ... ... ...  [1485]
JPY88.12  ... ... ..A ... ... ... ... ... ... ... ... ... ... ... ... ... ... ... ... ... ... ... ... ... ... ... ... ... ... ... ... ..G ...  [1485]
TN91.1292 ... ... ... ... ... ... ... ... ... ... ... ... ... ... ... ... ... ... ... ... ... ... ... ... ... ... ... ... ... ... ... ... ...  [1485]
TN92.313  ... ... ... ... ... ... ... ... ... ... ... ... ... ... ... ... ... ... ... ... ... ... ... ... ... ... ... ... ... ... ... ... ...  [1485]
TN92.250  ... ... ... ... ... ... ... ... ... ... ... ... ... ... ... ... ... ... ... ... ... ... ... ... ... ..T ... ... ... ... ... ... ...  [1485]
TN92.435  ... ... ... ... ... ... ... ... ... ... ... ... ... ... ... ... ... ... ... ... ... ... ... ... ... ... ... ... ... ... ... ... ...  [1485]
TN94.49   ... ... ... ... ... ... ... ... ... ... ... ..G ... ... ... ... ..G ... ... ... ... ... ... ... ... ... ... ... ... ... ... ... ...  [1485]
TN94.616  ... ... ... ... ... ... ... ... ... ... ... ... ... ... ... ... ... ... ... ... ... ... ... ... ... ..T ... ... ... ... ... ... ...  [1485]
TN94.66   ... ... ... ... ... ... ... ... ... ... ... ... ... ... ... ..T ... ... ... ... ... ... ... ... ... ... ... ... ... ... ... ..G ...  [1485]
TN95.354  ... ... ... ... ... ... ... ... ... ... ... ... ... ... ... ... ... ... ... ... ... ... ... ... ... ... ... ... ... ... ... ..G ...  [1485]
CAN97.83  ... ... ... ... ... ... ... ... ... ... ... ... ... ... ... ... ... ... ... ... ... ... ... ... ... ..T ... ... ... ... ... ... ...  [1485]
TN00.31   ... ... ... ... ... ... ... ... ... ..T ... ... ... ... ... ... ... ... ... ... ... ... ... ... ... ..T ... ... ... ... ... ... ...  [1485]
TN00.314  ... ... ... ... ... ... ... ... ... ..T ... ... ... ... ... ... ... ... ... ... ... ... ... ... ... ..T ... ... ... ... ... ... ...  [1485]
TN00.43   ... ... ... ... ... ... ... ... ... ... ... ... ... ... ... ... ... ... ... ... ... ... ..A ... ... ..T ... ... ... ... ... ... ...  [1485]
CAN00.16  ... ... ... .G. ... ... ... ... ... ... ... ... ... ... ... ... ... ... ... ... ... ... ... ... ... ..T ... ... ... ... ... ... ...  [1485]
NL00.17   ... ... ... ... ... ... ... ... ... ... ..A ... ... ... ... ... ... ... ... ... ... ... ... ... ... ..T ... ... ..T ... ... ... ...  [1485]
JPS03.178 ... ... ..A ... ... ... ... ... ... ... ... ... ... ... ... ... ... ... ... ... ... ... ... ... ... ... ... ... ... ... ... ..G ...  [1485]

TN82.79   ATT CTA ATT GCT GTC CTT GGC TCT AGC ATG ATC CTA GTG AGC ATT TTC ATT ATA ATC AAG AAA ACA AAG AAA CCA ACA GGA GCA CCT CCA GAG CTG AGT  [1584]
TN84.127  ... ... ... ... ... ... ... ... ... ... ... ... ... ... ... ... ... ... ..T ... ... ... ... ... ... ... ... ... ... ... ... ... ...  [1584]
TN85.115  ... ... ... ... ... ... ... ... ... ... ... ... ... ... ... ... ... ... ..T ... ... ... ... ... ... ... ... ... ... ... ... ... ...  [1584]
TN85.57   ... ... ... ... ... ... ... ... ... ... ... ... ... ... ... ... ... ... ..T ... ... ... ... ... ... ... ... ... ... ... ... ... ...  [1584]
TN85.63   ... ... ... ... ... ... ... ... ... ... ... ... ... ... ... ... ... ... ..T ... ... ... ... ... ... ... ... ... ... ... ... ... ...  [1584]
TN86.314  ... ... ... ... ... ... ... ... ... ... ... ... ... ... ... ... ... ... ..T ... ... ... ... ... ... ... ... ... ... ... ... ... ...  [1584]
TN88.59   ... ... ... ... ... ... ... ... ... ... ... ... ... ... ... ... ... ... ... ... ... ... ... ... ... ... ... ... ... ... ... ... ...  [1584]
JPY88.12  ... ... ... ... ... ... ... ..C ... ... ... ... ... ... G.C ... ... ... ... ... ... ... .G. ... ... ... ... ... ... ... ... ... ...  [1584]
TN91.1292 ... ... ... ... ... ... ... ..C ... ... ... ... ... ... ... ... ... ... ... ... ... ... ... ... ... ... ... ... ... ... ... ... ...  [1584]
TN92.313  ... ... ... ... ... ... ... ... ... ... ... ... ... ... ... ... ... ... ... ... ... ... ... ... ... ... ... ... ... ... ... ... ...  [1584]
TN92.250  ... ... ... ... ... ... ... ... ... ... ... ... ... ... ... ... ... ... ... ... ... ... ... ... ... ... ... ... ... ... ... ... ...  [1584]
TN92.435  ... ... ... ... ... ... ... ..C ... ... ... ... ... ... ... ... ... ... ... ... ... ... ... ... ... ... ... ... ... ... ... ... ...  [1584]
TN94.49   ... ... ... ... ... ... ... ... ... ... ... ... ... ... ... ... ... ... ... ... ... ... ... ... .A. ... ... ... ... ... ... ... ...  [1584]
TN94.616  ... ... ... ... ... ... ... ... ... ... ... ... ... ... ... ... ... ... ... ... ... ... ... ... ... ... ... ... ... ... ... ... ...  [1584]
TN94.66   ... T.. ... ... ... ... ... ..C ... ... ... ... ... ... ..C ... ... ... ... ... ... ... ... ... ... ... ... ... ... ... ... ... ...  [1584]
TN95.354  ... ... ... ... ... ... ... ..C ... ... ... ... ... ... ..C ... ... ... ... ... ... ... ... ... ... ... ... ... ... ... ... ... ...  [1584]
CAN97.83  ... ... ... ... ... ... ... ... ... ... ... ... ... ... ..C ... ... ... ... ... ... ... ... ... ... ..G ... ... ... ... ... ... ...  [1584]
TN00.31   ... ... ... ... ... ... ... ..C ... ... ... ... ... ... ..C ... ... ... ... ... ... ... ... ... ... ... ... ... ... ... ... ... ...  [1584]
TN00.314  ... ... ... ... ... ... ... ... ... ... ... ... ... ... ... ... ... ... ... ... ... ... ... ... ... ... ... ... ... ... ... ... ...  [1584]
TN00.43   ... ... ... ... ... ... ..A ... ... ... ... ... ... ... ... ... ... ... ... ... ... ... ... ... ... ... ... ... ... ... ... ... ...  [1584]
CAN00.16  ... ... ... ... ... ... ... ... ... ... ... ... ... ... ..C ... ... ... ... ... ... ... ... ... ... ..G ... ... ... ... ... ... ...  [1584]
NL00.17   ... ... ... ... ... ... ... ... ... ... ... ... ... ... ..C ... ... ... ... ... ... ... ... ... ... ..G ... ... ... ... ... ... ...  [1584]
JPS03.178 ... ... ... ... ... ... ... ..C ... ... ... ... ... ... G.C ... ... ... ... ... ... ... ... ... ... ... ... ... ... ... ... ... ...  [1584]

TN82.79   GGT GTC ACA AAC AAT GGC TTC ATA CCA CAC AGT TAG - [1621]
TN84.127  ... ... ... ... ... ... ... ... ... ... ... ... - [1621]
TN85.115  ... ... ... ... ... ... ... ... ... ... ... ... - [1621]
TN85.57   ... ... ... ... ... ... ... ... ... ... ... ... - [1621]
TN85.63   ... ... ... ... ... ... ... ... ... ... ... ... - [1621]
TN86.314  ... ... ... ... ... ... ... ... ... ... ... ... - [1621]
TN88.59   ... ... ... ... ... ... ... ... ... ... ... ... - [1621]
JPY88.12  ... ... ... ... ... ... ... ... ... ..T .A. ... - [1621]
```

```
TN91.1292 ... ... ... ... ... ... ... ... ... ... ... ... - [1621]
TN92.313  ..C ... ... ... ... ... ... ... ... ... ... ... - [1621]
TN92.250  ... ... ... ... ... ... ... ... ... ... ... ... - [1621]
TN92.435  ... ... ... ... ... ... ... ... ... ... ... ... - [1621]
TN94.49   ... ... ... ... ... ... ... ... ... ... ... ... - [1621]
TN94.616  ... ... ... ... ... ... ... ... ... ... ... ... - [1621]
TN94.66   ... ... ... ... ... ... ... ... ... ... ... ... - [1621]
TN95.354  ... ... ... ... ... ... ... ... ... ... ... ... - [1621]
CAN97.83  ... ... ... ... ... ... ... ... ... ... ... ... - [1621]
TN00.31   ... ... ... ... ... ... ... ... ... ... ... ... - [1621]
TN00.314  ... ... ... ... ... ... ... ... ... ... ... ... - [1621]
TN00.43   ... ... ... ... ... ... ... ... ... ... ... ... - [1621]
CAN00.16  ... ... ... ... ... ... ... ... ... ... ... ... - [1621]
NL00.17   ... ... ... ... ... ... ... ... ... ... ... ... - [1621]
JPS03.178 ... ... ... ... ... ... ... ... ..G ... ... ... - [1621]
```

Supplemental figure 2. Nucleotide sequence alignment of full-length F genes from subgroup A2 HMPV isolates. Sequences are arranged in chronological order from top to bottom. The letters indicate origin: "TN" = Tennessee; "CAN" = Canada; "NL" = Netherlands; and "JPS" = Japan. The first two digits of each isolate indicate the year; the next digit(s) for TN sequences indicate month of original virus isolation. Sequences from Canada, the Netherlands and Japan obtained from GenBank as described in the text. TN sequences have been deposited in GenBank.
