# Supplementary material for: Genetic diversity and evolution of human metapneumovirus fusion protein over twenty years
Source: Virol J. 2009 Sep 9;6:138. doi: 10.1186/1743-422X-6-138 (PMC2753315; doi:10.1186/1743-422X-6-138)
Supplement: Additional file 3 — Supplemental Figure 3. Nucleotide sequence alignment of full-length F genes from subgroup B1 HMPV isolates, listed in chronological order. [file 1743-422X-6-138-S3.pdf]

Supplemental Figure 3

```
TN87.27   ATG TCT TGG AAA GTG ATG ATC ATC ATT TCG TTA CTC ATA ACA CCC CAG CAC GGG CTA AAG GAG AGT TAT TTG GAA GAA TCA TGC AGT ACT ATA ACT GAG  [  99]
TN88.421  ... ... ... ... ... ... ... ... ... ... ... ... ... ... ... ... ... ... ... ... ... ... ... ... ... ... ... ... ... ... ... ... ...  [  99]
TN89.713  ... ... ... ... ... ... ... ... ... ... ... ... ... ... ... ... ... ... ... ... ... ... ... ... ... ... ... ..T ... ... ... ... ...  [  99]
TN94.11   ... ... ... ... ... G.. ... ... ... ... ... ... ... ... ... ... ... ... ... ... ... ... ... ... ... ... ... ... ... ... ... ... ...  [  99]
TN94.137  ... ... ... ... ... G.. ... ... ... ... ... ... ... ... ... ... ... ... ... ... ... ... ... ... ... ... ... ... ... ... ... ... ...  [  99]
CAN97.82  ... ... ... ... ... G.. ... ... ... ... ... ... ... ... ... ... ... ..A ... ... ... ... ... ... ... ... ... ... ... ... ... ... ...  [  99]
TN97.237  ... ... ... ... ... ... ... ... ... ... ... ... ... ... ... ... ... ... ... ... ... ... ... ... ... ... ... ..T ... ... ... ... ...  [  99]
TN98.242  ... ... ... ... ... ... ... ... ... ... ... ... ... ... ... ... ... ... ... ... ... ... ... ... ... ... ... ..T ... ... ... ... ...  [  99]
NL99.1    ... ... ... ... ... ... ... ... ... ... ... ... ... ... ... ... ... ... ... ... ... ... ... ... ... ... ... ..T ... ... ... ... ...  [  99]
JPS02.76  ... ... ... ... ... ... ... ... ... ... ... ... ... ... ... ... ... ... ... ... ... ... ... ... ... ... ... ... ... ... ... ... ...  [  99]
JPS03.194 ... ... ... ... ... ... ... ... ... ... ... ... ... ... ... ... ... ... ... ... ... ... ... ... ... ... ... ... ... ... ... ... ...  [  99]

TN87.27   GGA TAC CTC AGT GTT TTA AGA ACA GGC TGG TAC ACT AAT GTC TTT ACA TTA GAA GTT GGT GAT GTT GAA AAT CTT ACA TGT ACT GAT GGA CCT AGC TTA  [ 198]
TN88.421  ... ... ... ... ... ... ... ... ..T ... ... ... ... ... ... ... ... ... ... ... ... ... ... ... ... ... ... ... ... ... ... ... ...  [ 198]
TN89.713  ... ... ... ... ... ... ... ... ... ... ... ... ... ... ..C ... ... ... ... ... ... ... ... ... ... ... ... ... ... ... ... ... ...  [ 198]
TN94.11   ... ... ... ... ... ... ... ... ... ... ... ... ... ... ..C ... ... ... ... ... ... ... ... ... ... ... ... ... ... ... ... ... ...  [ 198]
TN94.137  ... ... ... ... ... ... ... ... ... ... ... ... ... ... ..C ... ... ... ... ... ... ... ... ... ... ... ... ... ... ... ... ... ...  [ 198]
CAN97.82  ... ... ... ... ... ... ... ... ... ... ... ... ... ... ..C ... ... ... ... ... ... ... ... ... ... ... ... ... ... ... ... ... ...  [ 198]
TN97.237  ... ... ... ... ... ... ... ... ... ... ... ... ... ... ..C ... ... ... ... ... ... ... ... ... ... ... ... ... ... ... ... ... ...  [ 198]
TN98.242  ... ... ... ... ... ... ... ... ... ... ... ... ... ... ..C ... ... ... ... ... ... ... ... ... ... ... ... ... ... ... ... ... ...  [ 198]
NL99.1    ... ... ... ... ... ... ... ... ... ... ... ... ... ... ..C ... ... ... ... ... ... ... ... ... ... ... ... ... ... ... ... ... ...  [ 198]
JPS02.76  ... ... ... ... ... ... ... ... ... ... ... ... ... ... ..C ... ... ... ... ... ... ... ... ... ... ... ... ... ... ... ... ... ...  [ 198]
JPS03.194 ... ..T ... ..C ... ... ... ... ... ... ... ... ... ... ..C ... ... ... ... ... ... ... ..G ... ... ... ... ... ... ..G ... ... ...  [ 198]

TN87.27   ATC AAA ACA GAA CTT GAC CTA ACC AAA AGT GCT TTA AGG GAA CTC AAA ACA GTC TCT GCT GAT CAG TTA GCG AGA GAG GAG CAA ATT GAA AAT CCC AGA  [ 297]
TN88.421  ... ... ... ... ... ... ... ... ... ... ... ... ... ... ... ... ... ... ... ... ... ... ... ... ... ... ... ... ... ... ... ... ...  [ 297]
TN89.713  ... ... ... ... ... ..T ... ..A ... ... ... ... ... ... ... ... ... ... ... ... ... ... ..G ... ... ... ... ... ... ... ... ... ...  [ 297]
TN94.11   ... ... ... ... ... ... ... ..A ... ... ... ... ... ... ... ... ... ... ... ... ... ... ... ... ... ... ... ... ... ... ... ... ...  [ 297]
TN94.137  ... ... ... ... ... ... ... ..A ... ... ... ... ... ... ... ... ... ... ... ... ... ... ... ... ... ... ... ... ... ... ... ... ...  [ 297]
CAN97.82  ... ... ... ... ... ... ... ..A ... ... ... ... ... ... ... ... ... ... ... ... ... ... ..G ... ... ... ... ..G ... ... ... ... ...  [ 297]
TN97.237  ... ... ... ... ... ... ... ..A ... ... ... ... ... ... ... ... ... ... ... ... ... ... ... ... ... ... ... ... ... ... ... ... ...  [ 297]
TN98.242  ... ... ... ... ... ... ... ..A ... ... ... ... ... ... ... ... ... ... ... ... ... ... ... ... ... ... ... ... ... ... ... ... ...  [ 297]
NL99.1    ... ... ... ... ... ..T ... ..A ... ... ... ... ... ... ... ... ... ... ... ... ... ... ..G ... ... ... ... ... ... ... ... ... ...  [ 297]
JPS02.76  ... ... ... ... ... ... ... ..A ... ... ... ... ... ... ... ... ... ... ... ... ... ... ... ..T ... ... ... ... ... ... ... ... ...  [ 297]
JPS03.194 ... ... ... ... ... ... ... ..A ... ... ... ... ... ... ... ... ... ... ... ... ... ... ... ... ... ... ... ... ... ... ... ... ...  [ 297]

TN87.27   CAA TCA AGA TTT GTC CTA GGT GCA ATA GCT CTC GGA GTT GCT ACA GCA GCA GCA GTC ACA GCA GGT ATT GCA ATA GCC AAA ACC ATA AGG CTT GAA AGT  [ 396]
TN88.421  ... ... ... ... ... ... ... ... ... ... ... ... ... ... ... ... ... ... ... ... ... ... ... ... ... ... ... ... ... ... ... ..G ...  [ 396]
TN89.713  ... C.. ... ... ... T.. ... ..G ... ... ... ... ... ... ... ... ... ... ... ... ... ..C ... ... ... ... ... ... ... ... ... ..G ...  [ 396]
TN94.11   ... ... ... ... ... ... ... ... ... ... ... ... ... ... ... ... ... ... ... ... ... ..C ..C ... ... ... ... ..T ... ... ... ..G ...  [ 396]
TN94.137  ... ... ... ... ... ... ... ... ... ... ... ... ... ... ... ... ... ... ... ... ... ..C ..C ... ... ... ... ..T ... ... ... ..G ...  [ 396]
CAN97.82  ... ... ... ... ... ... ... ... ... ... ... ... ... ... ... ... ... ... ... ... ... ..C ... ... ... ... ... ... ... ... ... ..G ...  [ 396]
TN97.237  ... ... ... ... ... ... ... ... ... ... ... ... ... ... ... ... ... ... ... ... ... ... ... ... ... ... ... ... ... ... ... ..G ...  [ 396]
TN98.242  ... ... ... ... ... ... ... ... ... ... ... ... ... ... ... ... ... ... ... ... ... ... ... ... ... ... ... ... ... ... ... ..G ...  [ 396]
NL99.1    ... ... ... ... ... T.. ... ..G ... ... ... ... ... ... ... ... ... ... ... ... ... ..C ... ... ... ... ... ... ... ... ... ..G ...  [ 396]
JPS02.76  ... ... ... ... ... ... ... ... ... ... ... ... ... ... ... ... ... ... ... ... ... ..C ... ... ... ... ... ... ... ... ... ..G ...  [ 396]
JPS03.194 ... ... ... ... ... ... ... ... ... ... ... ... ... ... ... ... ... ... ... ... ... ..C ... ... ... ... ... ... ... ... ... ..G ...  [ 396]

TN87.27   GAA GTG AAC GCA ATC AAA GGT GCT CTC AAA CAA ACC AAT GAA GCA GTA TCC ACA TTA GGA AAT GGA GTG CGG GTC CTA GCC ACT GCA GTG AGA GAG CTG  [ 495]
TN88.421  ... ... ..T ... ... ... ... ... ... ... ... ..T ... ... ... ... ... ... ... ... ... ... ... ... ... ... ... ... ... ... ... ... ...  [ 495]
TN89.713  ..G ... ..T ... ..T ... ... ... ... ... ... ..T ... ... ... ... ... ... ... ..G ... ..T ... ... ... ... ... ... ... ... ... ... ..A  [ 495]
TN94.11   ... ... ..T ... ... ... ... ... ... ... ... ..T ... ... ... ... ... ... ... ... ... ... ... ... ... ... ... ... ... ... ... ... ...  [ 495]
TN94.137  ... ... ..T ... ... ... ... ... ... ... ... ..T ... ... ... ... ... ... ... ... ... ... ... ... ... ... ... ... ... ... ... ... ...  [ 495]
CAN97.82  ..G ... ..T ... ..T ... ... ... ... ... ... ..T ... ... ... ... ... ... ... ... ... ..T ... ... ... ... ... ... ... ... ... ... ...  [ 495]
```

```
TN97.237  ..G ... ..T ... ..T ... ... ... ... ... ... ..T ... ... ... ... ... ... ... ... ... ..T ... ... ... ... ... ... ... ... ... ... ...  [ 495]
TN98.242  ..G ... ..T ... ..T ... ... ... ... ... ... ..T ... ... ... ... ... ... ... ... ... ..T ... ... ... ... ... ... ... ... ... ... ...  [ 495]
NL99.1    ..G ... ..T ... ..T ... ... ... ... ... ... ..T ... ... ... ... ... ... ... ..G ... ..T ... ... ... ... ... ... ... ... ... ... ..A  [ 495]
JPS02.76  ..G ... ..T ... ..T ... ... ... ... ... ... ..T ... ... ... ... ... ... ... ... ... ..T ... ... ... ... ... ... ... ... ... ... ..A  [ 495]
JPS03.194 ..G ... ..T ... ..T ... ... ... ... ... ... ..T ... ... ... ... ... ... ... ... ... ..T ... ... ... ... ... ... ... ... ... ..A ..A  [ 495]

TN87.27   AAA GAA TTT GTG AGC AAA AAC CTG ACT AGT GCA ATC AAC AAG AAC AAA TGT GAC ATT GCT GAT CTG AAG ATG GCT GTC AGC TTC AGT CAA TTC AAC AGA  [ 594]
TN88.421  ... ... ... ... ... ... ... ... ... ... ... ... ... ... ... ... ... ... ... ... ... ... ... ... ... ... ... ... ... ... ... ... ...  [ 594]
TN89.713  ... ... ... ... ... ... ... ... ... ... ... ... ... .G. ... ... ... ... ... ... ... ... ... ... ... ... ... ... ... ... ... ... ...  [ 594]
TN94.11   ... ... ... ... ... ... ... ... ... ... ... ... ... .G. ... ... ... ... ... ... ... ... ... ... ... ... ... ... ... ... ... ... ...  [ 594]
TN94.137  ... ... ... ... ... ... ... ... ... ... ... ... ... .G. ... ... ... ... ... ... ... ... ... ... ... ... ... ... ... ... ... ... ...  [ 594]
CAN97.82  ... ... ... ... ... ... ... ... ... ... ... ... ... .G. ... ... ... ... ... ... ... ... ... ... ... ... ... ... ... ... ... ... ...  [ 594]
TN97.237  ... ... ... ... ... ... ... ... ... ... ... ... ... .G. ... ... ... ... ... ... ... ... ... ... ... ... ... ... ... ... ... ... ...  [ 594]
TN98.242  ... ... ... ... ... ... ... ... ... ... ... ... ... .G. ... ... ... ... ... ... ... ... ... ... ... ... ... ... ... ... ... ... ...  [ 594]
NL99.1    ... ... ... ... ... ... ... ... ... ... ... ... ... .G. ... ... ... ... ... ... ... ... ... ... ... ... ... ... ... ... ... ... ...  [ 594]
JPS02.76  ... ... ... ... ... ... ..T ... ... ... ... ... ... .G. ... ... ... ... ... ... ... ... ... ... ... ... ... ... ... ... ... ... ...  [ 594]
JPS03.194 ... ... ... ... ... ... ..T ... ... ... ... ... ... .G. ... ... ... ... ... ... ... ... ... ... ... ... ..T ... ... ... ... ... ...  [ 594]

TN87.27   AGA TTT CTA AAT GTT GTG CGG CAG TTT TCA GAC AAT GCA GGG ATA ACA CCA GCA ATA TCA TTG GAC CTA ATG ACT GAT GCT GAG TTG GCC AGA GCT GTA  [ 693]
TN88.421  ... ... ... ... ... ... ... ... ... ... ... ... ... ... ... ... ... ... ... ... ... ... ... ... ... ... ... ... ... ... ... ... ...  [ 693]
TN89.713  ... ... ... ... ... ... ... ... ... ... ... ... ... ... ... ... ... ... ... ... ... ... ..G ... ... ... ... ... ... ... ... ... ...  [ 693]
TN94.11   ... ... ... ... ... ... ... ... ... ... ... ... ... ... ... ... ... ... ... ... ... ... ... ... ... ... ... ... ... ... ... ... ...  [ 693]
TN94.137  ... ... ... ... ... ... ... ... ... ... ... ... ... ... ... ... ... ... ... ... ... ... ... ... ... ... ... ... ... ... ... ... ...  [ 693]
CAN97.82  ... ... ... ... ... ... ... ... ... ... ... ... ... ... ... ... ... ... ... ... ... ... ... ... ... ... ... ... ... ... ... ... ...  [ 693]
TN97.237  ... ... ... ... ... ... ... ... ... ... ... ... ... ... ... ... ... ... ... ... ... ... ... ... ... ... ... ... ... ... ... ... ...  [ 693]
TN98.242  ... ... ... ... ... ... ... ... ... ... ... ... ... ... ... ... ... ... ... ... ... ... ... ... ... ... ... ... ... ... ... ... ...  [ 693]
NL99.1    ... ... ... ... ... ... ... ... ... ... ... ... ... ... ... ... ... ... ... ... ... ... ..G ... ... ... ... ... ... ... ... ... ...  [ 693]
JPS02.76  ... ... ... ... ... ... ... ... ... ... ... ... ... ... ... ... ... ... ... ... ... ... ... ... ... ... ... ..A ... ... ... ... ...  [ 693]
JPS03.194 ... ... ... ... ... ... A.. ... ... ... ... ... ... ... ... ... ... ... ... ... ... ... ... ... ... ... ... ..A ... ... ... ... ...  [ 693]

TN87.27   TCA TAC ATG CCA ACA TCT GCA GGA CAG ATA AAA CTA ATG TTG GAG AAC CGC GCA ATG GTA AGG AGG AAA GGA TTT GGA ATC CTG ATA GGG GTC TAC GGA  [ 792]
TN88.421  ... ... ... ... ... ... ... ... ... ... ... ... ... ... ... ... ... ... ... ... ... ... ... ... ... ... ... ... ... ... ... ... ...  [ 792]
TN89.713  ... ... ... ... ... ... ... ..G ... ... ... ..G ... ... ... ... ... ... ... ... ... ..A ... ... ... ... ... ... ... ... ... ... ...  [ 792]
TN94.11   ... ... ... ... ... ... ... ..G ... ... ... ..G ... ... ... ... ... ... ... ... ... ..A ... ... ... ... ... ... ... ... ... ... ...  [ 792]
TN94.137  ... ... ... ... ... ... ... ..G ... ... ... ..G ... ... ... ... ... ... ... ... ... ..A ... ... ... ... ... ... ... ... ... ... ...  [ 792]
CAN97.82  ... ... ... ... ... ... ... ... ... ... ... ..G ... ... ... ... ... ... ... ... ... ..A ... ... ... ... ... ... ... ... ... ... ...  [ 792]
TN97.237  ... ... ... ... ... ... ... ... ... ... ... ..G ... ... ... ... ... ... ... ... ... ..A ... ... ... ... ... ... ... ... ... ... ...  [ 792]
TN98.242  ... ... ... ... ... ... ... ... ... ... ... ..G ... ... ... ... ... ... ... ... ... ..A ... ... ... ... ... ... ... ... ... ... ...  [ 792]
NL99.1    ... ... ... ... ... ... ... ..G ... ... ... ..G ... ... ... ... ... ... ... ... ... ..A ... ... ... ... ... ... ... ... ... ... ...  [ 792]
JPS02.76  ... ... ... ... ... ... ... ... ... ... ... ..G ... ... ... ... ... ... ... ... ... ..A ... ... ... ... ... ..A ... ... ... ... ...  [ 792]
JPS03.194 ... ... ... ... ... ... ... ..G ... ... ... ..G ... ... ... ... ... ... ... ... ... ..A ... ... ... ... ... ..A ... ... ... ... ...  [ 792]

TN87.27   AGC TCT GTG ATT TAC ATG GTT CAA TTG CCG ATC TTT GGT GTC ATA GAT ACA CCT TGT TGG ATA ATC AAG GCA GCT CCC TCT TGC TCA GAA AAA AAC GGA  [ 891]
TN88.421  ... ... ... ... ... ... ... ... ... ... ... ... ... ... ... ... ... ... ... ... ... ... ... ... ... ... ... ... ... ... ... ... ...  [ 891]
TN89.713  ... ... ... ... ... ... ... ... ... ... ... ... ... ... ... .G. ... ... ... ... ..C ... ... ... ... ... ... ... ... ... ... ... ..G  [ 891]
TN94.11   ... ... ... ... ... ... ... ... ... ... ... ... ... ... ... ... ... ... ... ... ... ... ... ... ... ... ... ... ... ... ... ... ..G  [ 891]
TN94.137  ... ... ... ... ... ... ... ... ... ... ... ... ... ... ... ... ... ... ... ... ... ... ... ... ... ... ... ... ... ... ... ... ..G  [ 891]
CAN97.82  ... ... ... ... ... ... ... ... ... ... ... ... ... ... ... ... ... ... ... ... ... ... ... ... ... ... ... ... ... ... ... ... ..G  [ 891]
TN97.237  ... ... ... ... ... ... ... ... ... ... ... ... ... ... ... ... ... ..C ... ... ... ... ... ... ... ... ... ... ... ... ... ... ..G  [ 891]
TN98.242  ... ... ... ... ... ... ... ... ... ... ... ... ... ... ... ... ... ..C ... ... ... ... ... ... ... ... ... ... ... ... ... ... ..G  [ 891]
NL99.1    ... ... ... ... ... ... ... ... ... ... ... ... ... ... ... ... ... ... ... ... ..C ... ... ... ... ... ... ... ... ... ... ... ..G  [ 891]
JPS02.76  ... ... ... ... ... ... ... ... ... ... ... ... ... ... ... ... ... ... ... ... .C. ... ... ... ... ... ... ... ... ... ... ... ..G  [ 891]
JPS03.194 ... ... ... ... ... ... ... ... ... ... ... ... ... ... ... ... ... ... ... ... ... ... ... ... ... ... ... ... ... ... ... ... ..G  [ 891]
```

```
TN87.27    AAT TAT GCT TGC CTC CTA AGA GAG GAT CAA GGG TGG TAT TGT AAA AAT GCA GGA TCC ACT GTT TAC TAC CCA AAT GAA AAA GAC TGC GAA ACA AGA GGT  [ 990]
TN88.421   ... ... ... ... ... ... ... ... ... ... ... ... ... ... ... ... ... ... ... ... ... ... ... ... ... ... ... ... ... ... ... ... ...  [ 990]
TN89.713   ... ... ... ... ... ... ... ... ... ... ... ... ... ... ... ... ... ... ..T ... ... ... ... ... ... ... ... ... ... ... ... ... ...  [ 990]
TN94.11    ... ... ... ... ... ... ... ... ... ... ... ... ... ... ... ... ... ... ... ... ... ... ... ... ... ... ... ... ... ... ... ... ...  [ 990]
TN94.137   ... ... ... ... ... ... ... ... ... ... ... ... ... ... ... ... ... ... ... ... ... ... ... ... ... ... ... ... ... ... ... ... ...  [ 990]
CAN97.82   ... ... ... ... ... ... ... ... ... ... ... ... ... ... ... ... ... ... ... ... ... ... ... ... ... ... ... ... ..T ... ... ... ...  [ 990]
TN97.237   ... ... ... ... ... ... ... ... ... ... ... ... ... ... ... ... ... ... ... ... ... ... ... ... ... ... ... ... ... ... ... ... ...  [ 990]
TN98.242   ... ... ... ... ... ... ... ... ... ... ... ... ... ... ... ... ... ... ... ... ... ... ... ... ... ... ... ... ... ... ... ... ...  [ 990]
NL99.1     ... ... ... ... ... ... ... ... ... ... ... ... ... ... ... ... ... ... ..T ... ... ... ... ... ... ... ... ... ... ... ... ... ...  [ 990]
JPS02.76   ... ... ... ... ... ... ... ... ... ... ... ... ... ... ... ... ... ... ... ... ... ... ... ... ... ... ... ... ..T ... ... ... ...  [ 990]
JPS03.194  ... ... ... ... ... ... ... ... ... ... ... ... ... ... ... ... ... ... ... ... ... ... ... ... ... ... ... ... ..T ... ... ... ...  [ 990]

TN87.27    GAT CAT GTT TTT TGT GAC ACA GCA GCA GGG ATC AAT GTT GCT GAG CAA TCA AGA GAA TGC AAC ATC AAC ATA TCT ACT ACC AAC TAC CCA TGC AAA GTC  [1089]
TN88.421   ... ... ... ... ... ... ... ... ... ... ... ... ... ... ... ... ... ... ... ... ... ... ... ... ... ... ... ... ... ... ... ... ...  [1089]
TN89.713   ... ... ... ... ... ... ... ... ... ... ... ... ... ... ... ... ... ... ... ... ... ... ... ... ... ... ... ... ... ... ... ... ...  [1089]
TN94.11    ... ... ... ... ... ... ... ... ... ... ... ... ... ... ... ... ... ... ... ... ... ... ... ... ... ... ... ... ... ... ... ... ...  [1089]
TN94.137   ... ... ... ... ... ... ... ... ... ... ... ... ... ... ... ... ... ... ... ... ... ... ... ... ... ... ... ... ... ... ... ... ...  [1089]
CAN97.82   ... ... ... ... ... ... ... ... ... ... ... ... ... ... ... ... ... ... ... ... ... ... ... ... ... ... ... ... ... ... ... ... ...  [1089]
TN97.237   ... ... ... ... ... ... ... ... ... ... ... ... ... ... ... ... ... ... ... ... ... ... ... ... ... ... ... ... ... ... ... ... ...  [1089]
TN98.242   ... ... ... ... ... ... ... ... ... ... ... ... ... ... ... ... ... ... ... ... ... ... ... ... ... ... ... ... ... ... ... ... ...  [1089]
NL99.1     ... ... ... ... ... ... ... ... ... ... ... ... ... ... ... ... ... ... ... ... ... ... ... ... ... ... ... ... ... ... ... ... ...  [1089]
JPS02.76   ... ... ... ... ... ... ... ... ... ... ... ... ..C ... ... ... ... ... ... ... ... ... ... ... ... ... ... ... ... ... ... ... ...  [1089]
JPS03.194  ... ... ... ... ... ... ... ... ... ... ... ... ... ... ... ... ... ... ... ... ... ... ... ... ... ... ... ... ... ... ... ... ...  [1089]

TN87.27    AGC ACA GGA AGA CAC CCT ATA AGC ATG GTT GCA CTA TCA CCT CTC GGT GCT TTG GTG GCT TGC TAT AAA GGG GTA AGC TGC TCG ATT GGC AGC AAT CGG  [1188]
TN88.421   ... ... ... ... ... ... ... ... ... ... ... ... ... ... ... ... ... ... ... ... ... ... ... ... ... ... ... ... ... ..T ... ... ...  [1188]
TN89.713   ... ... ... ... ... ... ... ... ... ... ... ... ... ... ... ... ... ... ... ... ... ... ... ... ... ... ... ... ... ... ... ... T..  [1188]
TN94.11    ... ... ... ... ... ... ... ... ... ... ... ... ... ... ... ... ... ... ... ... ... ... ... ... ... ... ... ... ... ... ... ... ...  [1188]
TN94.137   ... ... ... ... ... ... ... ... ... ... ... ... ... ... ... ... ... ... ... ... ... ... ... ... ... ... ... ... ... ... ... ... ...  [1188]
CAN97.82   ... ... ... ... ... ... ... ... ... ... ... ... ... ... ... ... ... ... ... ... ... ... ... ... ... ... ... ... ... ... ... ... ...  [1188]
TN97.237   ... ... ... ... ... ... ... ... ... ... ... ... ... ... ... ... ... ... ... ... ... ... ... ... ... ... ... ... ... ... ... ... ...  [1188]
TN98.242   ... ... ... ... ... ... ... ... ... ... ... ... ... ... ... ... ... ... ... ... ... ... ... ... ... ... ... ... ... ... ... ... ...  [1188]
NL99.1     ... ... ... ... ... ... ... ... ... ... ... ... ... ... ... ... ... ... ... ... ... ... ... ... ... ... ... ... ... ... ... ... T..  [1188]
JPS02.76   ... ... ... ... ... ... ... ... ... ... ... ... ... ... ... ... ... ... ... ... ... ... ... ... ... ... ... ... ... ... ... ... ..A  [1188]
JPS03.194  ... ... ... ... ... ... ... ... ... ... ... ... ... ... ... ... ... ... ... ... ... ... ... ... ... ... ... ... ... ... ... ... ...  [1188]

TN87.27    GTT GGA ATC ATC AAA CAA CTA CCT AAA GGC TGC TCA TAC ATA ACC AAC CAG GAT GCA GAC ACT GTA ACA ATT GAC AAT ACC GTG TAT CAA CTA AGC AAA  [1287]
TN88.421   ... ... ... ... ... ... ... ... ... ... ... ... ... ... ... ... ... ... ... ... ... ... ... ... ... ... ... ... ... ... ... ... ...  [1287]
TN89.713   ... ... ... ... ... ... T.. ..C ... ... ... ... ... ... ... ... ... ... ... ... ... ... ... ... ... ... ... ... ... ... ... ... ...  [1287]
TN94.11    ... ... ... ... ... ... T.. ... ... ... ... ... ... ... ... ... ... ... ... ... ... ... ... ... ... ... ... ... ... ... ... ... ...  [1287]
TN94.137   ... ... ... ... ... ... T.. ... ... ... ... ... ... ... ... ... ... ... ... ... ... ... ... ... ... ... ... ... ... ... ... ... ...  [1287]
CAN97.82   ... ... ... ... ... ... T.. ... ... ... ... ... ... ... ... ... ... ... ... ... ... ... ... ... ... ... ... ... ... ... ... ... ...  [1287]
TN97.237   ... ... ... ... ... ... T.. ... ... ... ... ... ... ... ... ... ... ... ... ... ... ... ... ... ... ... ... ... ... ... ... ... ...  [1287]
TN98.242   ... ... ... ... ... ... T.. ... ... ... ... ... ... ... ... ... ... ... ... ... ... ... ... ... ... ... ... ... ... ... ... ... ...  [1287]
NL99.1     ... ... ... ... ... ... T.. ..C ... ... ... ... ... ... ... ... ... ... ... ... ... ... ... ... ... ... ... ... ... ... ... ... ...  [1287]
JPS02.76   ... ... ... ... ... ... T.. ... ... ... ... ... ... ... ..T ... ... ... ... ... ... ... ... ... ... ... ... ... ... ... ... ... ...  [1287]
JPS03.194  ... ... ... ... ... ... T.. ... ... ... ... ... ... ... ..T ... ... ... ... ... ... ..G ... ... ... ... ... ... ... ... ... ... ...  [1287]

TN87.27    GTT GAA GGT GAA CAG CAT GTA ATA AAA GGG AGA CCA GTT TCA AGC AGT TTT GAT CCA ATC AGG TTT CCT GAG GAT CAG TTC AAT GTT GCG CTT GAT CAA  [1386]
TN88.421   ... ... ... ... ... ... ... ... ... ... ... ... ... ... ... ... ... ... ... ... ... ... ... ... ... ... ... ... ... ... ... ... ...  [1386]
TN89.713   ... ... ... ... ... ... ... ... ... ... ... ... ... ... ... ... ... ... ... ... .A. ... ... ... ... ... ... ... ... ... ... ... ...  [1386]
TN94.11    ... ... ... ... ... ... ... ... .G. ... ... ... ... ... ... ... ... ..C ... ... ... ... ... ... ... ... ... ... ... ... ... ... ...  [1386]
TN94.137   ... ... ... ... ... ... ... ... .G. ... ... ... ... ... ... ... ... ..C ... ... ... ... ... ... ... ... ... ... ... ... ... ... ...  [1386]
CAN97.82   ... ... ... ... ... ... ... ... ... ... ... ... ... ... ... ... ... ... ... ... ... ... ... ... ... ... ... ... ... ... ... ... ...  [1386]
```

```
TN97.237   ... ... ... ... ... ... ... ... ... ... ..G ... ... ... ... ... ... ... ... ... ... ... ... ... ... ... ... ... ... ... ... ... ...  [1386]
TN98.242   ... ... ... ... ... ... ... ... ... ... ..G ... ... ... ... ... ... ... ... ... ... ... ... ... ... ... ... ... ... ... ... ... ...  [1386]
NL99.1     ... ... ... ... ... ... ... ... ... ... ... ... ... ... ... ... ... ... ... ... .A. ... ... ... ... ... ... ... ... ... ... ... ...  [1386]
JPS02.76   ... ... ... ... ... ... ... ... ... ... ... ... ... ... ... ... ... ... ... ... ... ... ... ... ... ... ... ... ... ... ... ... ...  [1386]
JPS03.194  ... ... ... ... ... ... ... ... ... ... ... ... ... ... ... ... ... ... ... ... ... ... ... ... ... ... ... ... ... ... ... ... ...  [1386]

TN87.27    GTC TTT GAA AGC ATT GAA AAC AGT CAG GCA CTA GTG GAC CAG TCA AAC AGA ATT CTA AAC AGT GCA GAA AAA GGA AAC ACT GGT TTC ATT ATT GTA ATA  [1485]
TN88.421   ... ... ... ... ... ... ... ... ... ... ... ... ... ... ... ... ... ... ... ... ... ... ... ... ... ... ... ... ... ... ... ... ...  [1485]
TN89.713   ... ..C ... ... ... ..G ... ... ... ... ... ... ... ... ... ... .A. ... ... ... ... ... ... ... ... ... ... ... ... ... ..C ... G..  [1485]
TN94.11    ... ..C ... ... ... ..G ... ... ... ... ... ... ... ... ... ... .A. ... ... ... ... ... ... ... ... ... ... ... ... ... ... ... ...  [1485]
TN94.137   ... ..C ... ... ... ..G ... ... ... ... ... ... ... ... ... ... .A. ... ... ... ... ... ... ... ... ... ... ... ... ... ... ... ...  [1485]
CAN97.82   ... ..C ... ... ... ... ... ... ... ... ... ... ... ... ... ... .A. ... ... ... ... ... ... ... ... ... ... ..C ... ... ... ... ...  [1485]
TN97.237   ... ..C ... ... ... ..G ... ... ... ... ... ... ... ... ... ... .A. ... ... ... ... ... ... ... ... ... ... ... ... ... ... ... ...  [1485]
TN98.242   ... ..C ... ... ... ..G ... ... ... ... ... ... ... ... ... ... .A. ... ... ... ... ... ... ... ... ... ... ... ... ... ... ... ...  [1485]
NL99.1     ... ..C ... ... ... ..G ... ... ... ... ... ... ... ... ... ... .A. ... ... ... ... ... ... ... ... ... ... ... ... ... ..C ... G..  [1485]
JPS02.76   ... ..C ... ... ... ..G ... ... ... ... ..G ... ..A ... ... ... .A. ... ... ... ... ... ... ... ... ... ... ... ... ... ... ... ...  [1485]
JPS03.194  ... ..C ... ... ... ..G ... ... ... ... ..G ... ..A ... ... ... .A. ... ... ... ... ... ... ... ... ... ... ..C ... ... ... ... ...  [1485]

TN87.27    ATT TTG GTT GCC GTT CTT GGT TTA ACC ATG ATT TCA GTG AGC ATC ATC ATC ATA ATC AAG AAA ACA AGG AAG CCC ACA GGA GCA CCT CCA GAG CTG AAT  [1584]
TN88.421   ... ... ... ... ... ... ... ... ... ... ... ... ... ... ... ... ... ... ... ... ... ... ... ... ... ... ... ... ... ... ... ... ...  [1584]
TN89.713   ... ... ... ..T ... ... ... C.. ... ... ... ... ... ... ... ... ... ... ... ... ... ... ... ... ... ... ... ... ... ... ... ... ...  [1584]
TN94.11    ... ... ... ..T ... ... ... ... ... ... ... ... ... ... ... ... ... ... ... ... ... ... ... ... ... ... ... ... ... ... ... ... ...  [1584]
TN94.137   ... ... ... ..T ... ... ... ... ... ... ... ... ... ... ... ... ... ... ... ... ... ... ... ... ... ... ... ... ... ... ... ... ...  [1584]
CAN97.82   ... ... ... ..T ... ... ... ... ... ... ... ... ... ... ... ... ... ... ... ... ... ... ... ... ..A ... ... ... ... .T. ... ... ...  [1584]
TN97.237   ... ... ... ..T ... ... ... ... ... ... ... ... ... ... ... ... ... ... ... ... ... ... ... ... ... ... ... ... ... ... ... ... ...  [1584]
TN98.242   ... ... ... ..T ... ... ... ... ... ... ... ... ... ... ... ... ... ... ... ... ... ... ... ... ... ... ... ... ... ... ... ... ...  [1584]
NL99.1     ... ... ... ..T ... ... ... C.. ... ... ... ... ... ... ... ... ... ... ... ... ... ... ... ... ... ... ... ... ... ... ... ... ...  [1584]
JPS02.76   ... ... ... ..T ... ... ... ... ... ... ... ... ... ... ... ... ... ... ... ... ... ... ... ... ... ... ... ... ... ... ... ... ...  [1584]
JPS03.194  ... ... ... ..T ... ... ... ... ... ... ... ... ... ... ... ... ... ... ... ... ... ... ... ... ... ... ... ... ... ... ... ... ...  [1584]

TN87.27    GGT GTC ACC AAC GGC GGT TTC ATA CCG CAC AGT TAG - [1621]
TN88.421   ... ... ... ... ... ... ... ... ... ... ... ... - [1621]
TN89.713   ... ... ... ... ... ... ... ... ..A ..T ... ... - [1621]
TN94.11    ... ... ... ... ... ..G ... ... ..A ..T ... ... - [1621]
TN94.137   ... ... ... ... ... ..G ... ... ..A ..T ... ... - [1621]
CAN97.82   ... ... ... ... ... ... ... ... ..A ..T ... ... - [1621]
TN97.237   ... ... ... ... ... ... ... ... ..A ..T ... ... - [1621]
TN98.242   ... ... ... ... ... ... ... ... ..A ..T ... ... - [1621]
NL99.1     ... ... ... ... ... ... ... ... ..A ..T ... ... - [1621]
JPS02.76   ... ... ... ... ... ... ... ... ..A ..T ... ... - [1621]
JPS03.194  ... ... ... ... ..T ... ... ... ..A ..T ... ... - [1621]
```

Supplemental figure 3. Nucleotide sequence alignment of full-length F genes from subgroup B1 HMPV isolates. Sequences are arranged in chronological order from top to bottom. The letters indicate origin: "TN" = Tennessee; "CAN" = Canada; "NL" = Netherlands; and "JPS" = Japan. The first two digits of each isolate indicate the year; the next digit(s) for TN sequences indicate month of original virus isolation. Sequences from Canada, the Netherlands and Japan obtained from GenBank as described in the text. TN sequences have been deposited in GenBank.
