# Supplementary material for: Genetic diversity and evolution of human metapneumovirus fusion protein over twenty years
Source: Virol J. 2009 Sep 9;6:138. doi: 10.1186/1743-422X-6-138 (PMC2753315; doi:10.1186/1743-422X-6-138)
Supplement: Additional file 4 — Supplemental Figure 4. Nucleotide sequence alignment of full-length F genes from subgroup B2 HMPV isolates, listed in chronological order. [file 1743-422X-6-138-S4.pdf]

Supplemental Figure 4

```
TN82.59    ATG TCT TGG AAA GTG ATG ATT ATC ATT TCG TTA CTC ATA ACA CCT CAG CAT GGA CTA AAA GAA AGT TAT TTA GAG GAA TCA TGT AGT ACT ATA ACT GAA  [  99]
TN83.126   ... ... ... ... ... ... ... ... ... ... ... ... ... ... ... ... ... ... ... ... ... ... ... ... ..A ... ... ... ... ... ... ... ...  [  99]
TN83.1211  ... ... ... ... ... ... ... ... ... ... ... ... ... ... ... ... ... ... ... ... ... ... ... ... ..A ... ... ... ... ... ... ... ...  [  99]
TN84.611   ... ... ... ... ... ... ... ... ... ... ... ... ... ... ... ... ... ... ... ... ... ... ... ... ..A ... ... ... ... ... ... ... ...  [  99]
TN85.123   ... ... ... ... ... ... ... ... ... ... ... ... ... ... ... ... ... ... ... ... ... ... ... ... ..A ... ... ... ... ... ... ... ...  [  99]
TN85.417   ... ... ... ... ... ... ... ... ... ... ... ... ... ... ... ... ... ... ... ... ... ... ... ... ..A ... ... ... ... ... ... ... ...  [  99]
TN89.356   ... ... ... ... ... ... ... ... ... ... ... ... ... ... ... ... ..C ... ... ... ... ... ... ... ..A ... ... ... ... ... ... ... ...  [  99]
TN89.1144  ... ... ... ... ... ... ... ... ... ... ... ... ... ... ... ... ... ... ... ... ... ... ... ... ..A ... ... ... ... ... ... ... ...  [  99]
TN89.1259  ... ... ... ... ... ... ... ... ... ... ... ... ... ... ... ... ... ... ... ... ... ... ... ... ..A ... ... ... ... ... ... ... ...  [  99]
TN89.515   ... ... ... ... ... ... ... ... ... ... ... ... ... ... ... ... ... ... ... ... ... ... ... ... ..A ... ... ... ... ... ... ... ...  [  99]
TN90.140   ... ... ... ... ... ... ... ... ... ... ... ... ... ... ... ... ... ... ... ... ... ... ... ... ..A ... ... ... ... ... ... ... ...  [  99]
TN90.422   ... ... ... ... ... ... ... ... ... ... ... ... ... ... ... ... ... ... ... ... ... ... ... ... ..A ... ... ... ... ... ... ... ...  [  99]
TN90.1031  ... ... ... ... ... ... ... ... ... ... ... ... ... ... ... ... ..C ... ... ... ... ... ... C.. ..A ... ... ... ... ... ... ... ...  [  99]
TN91.316   ... ... ... ... ... ... ... ... ... ..A ... ... ... ... ... ... ..C ... ... ... ... ... ... ... ..A ... ... ... ... ... ... ... ...  [  99]
TN91.320   ... ... ... ... ... ... ... ... ... ... ... ... ... ... ... ... ..C ... ... ... ... ... ... ... ..A ... ... ... ... ... ... ... ...  [  99]
TN91.334   ... ... ... ... ... ... ... ... ... ... ... ... ... ... ... ... ... ... ... ... ... ... ... ... ..A ... ... ... ... ... ... ... ...  [  99]
TN91.349   ... ... ... ... ... ... ... ... ... ... ... ... ... ... ... ... ..C ... ... ... ... ... ... ... ..A ... ... ... ... ... ... ... ...  [  99]
TN91.451   ... ... ... ... ... ... ... ... ... ... ... ... ... ... ... ... ..C ... ... ... ... ... ... ... ..A ... ... ... ... ... ... ... ...  [  99]
TN91.521   ... ... ... ... ... ... ... ... ... ... ... ... ... ... ... ... ..C ... ... ... ... ... ... ... ..A ... ... ... ... ... ... ... ...  [  99]
TN92.1031  ... ... ... ... ... ... ... ... ... ... ... ... ... ... ... ... ..C ... ... ... ... ... ... ... ..A ... ... ... ... ... ... ... ...  [  99]
TN92.1032  ... ... ... ... ... ... ... ... ... ... ... ... ... ... ... ... ..C ... ... ... ... ... ... ... ..A ... ... ... ... ... ... ... ...  [  99]
TN92.1120  ... ... ... ... ... ... ... ... ... ... ... ... ... ... ... ... ..C ... ... ... ... ... ... ... ..A ... ... ... ... ... ... ... ...  [  99]
TN93.32    ... ... ... ... ... ... ... ... ... ... ... ... ... ... ... ... ..C ... ... ... ... ... ... ... ..A ... ... ... ... ... ... ... ...  [  99]
TN93.616   ... ... ... ... ... ... ... ... ... ... ... ... ... ... ... ... ..C ... ... ... ... ... ... ... ..A ... ... ... ... ... ... ... ...  [  99]
TN94.413   ... ... ... ... ... ... ... ... ... ... ... ... ... ... ... ... ..C ... ... ... ... ... ... ... ..A ... ... ... ... ... ... ... ...  [  99]
NL94.1     ... ... ... ... ... ... ... ... ... ... ... ... ... ... ... ... ..C ... ... ... ... ... ... ... ..A ... ... ... ... ... ... ... ...  [  99]
TN95.252   ... ... ... ... ... ... ... ... ... ... ... ... ... ... ... ... ..C ... ... ... ... ... ... ... ..A ... ... ... ... ... ... ... ...  [  99]
TN96.115   ... ... ... ... ... ... ... ... ... ... ... ... ... ... ... ... ..C ... ... ... ... ... ... ... ..A ... ... ... ... ... ... ... ...  [  99]
TN96.35    ... ... ... ... ... ... ... ... ... ... ... ... ... ... ... ... ..C ... ... ... ... ... ... ... ..A ... ... ... ... ... ... ... ...  [  99]
TN96.213   ... ... ... ... ... ... ... ... ... ... ... ... ... ... ... ... ..C ... ... ... ... ... ... ... ..A ... ... ... ... ... ... ... ...  [  99]
TN96.44    ... ... ... ... ... ... ... ... ... ... ... ... ... ... ... ... ..C ... ... ... ... .A. ... ... ..A ... ... ... ... ... ... ... ...  [  99]
TN97.235   ... ... ... ... ... ... ... ... ... ... ... ... ... ... ... ..A ... ... ... ... ... .A. ... ... ..A ... ... ... ..C ... ... ... ...  [  99]
TN98.512   ... ... ... ... ... ... ... ... ... ... ... ... ... ... ... ... ..C ... ... ... ... ... ... ... ..A ... ... ... ... ... ... ... ...  [  99]
TN99.419   ... ... ... ... ... ... ... ... ... ... ... ... ... ... ... ... ..C ... ... ... ... ... ... ... ..A ... ... ... ... ... ... ... ...  [  99]
CAN98.73   ... ... ... ... ... G.. ... ... ... ... ... ... ... ... ... ... ..C ... ... ... ... ... ... ... ..A ... ... ... ... ... ... ... ...  [  99]
CAN98.79   ... ... ... ... ... G.. ... ... ... ... ... ... ... ... ... ... ..C ... ... ... ... ... ... ... ..A ... ... ... ... ... ... ... ...  [  99]
CAN00.13   ... ... ... ... ... G.. ... ... ... ... ... ... ... ... ... ... ..C ... ... ... ... ... ... ... ..A ... ... ... ... ... ... ... ...  [  99]
TN01.28    ... ... ... ... ... ... ... ... ... ... ... ... ... ... ... ... ..C ... ... ... ... ... ... ... ..A ... ... ... ... ... ... ... ...  [  99]

TN82.59    GGA TAC CTC AGT GTT TTA AGA ACA GGT TGG TAC ACC AAT GTC TTT ACA TTA GAA GTT GGT GAT GTT GAA AAT CTT ACA TGT ACT GAT GGA CCT AGC TTA  [ 198]
TN83.126   ... ... ... ... ... ... ... ... ... ... ... ... ... ... ... ... ... ... ... ... ... ... ... ... ... ... ... ... ... ... ... ... ...  [ 198]
TN83.1211  ... ... ... ... ... ... ... ... ... ... ... ... ... ... ... ... ... ... ... ... ... ... ... ... ... ... ... ... ... ... ... ... ...  [ 198]
TN84.611   ... ... ... ... ... ... ... ... ... ... ... ... ... ... ... ... ... ... ... ... ... ... ... ... ... ... ... ... ... ... ... ... ...  [ 198]
TN85.123   ... ..T ... ... ... ... ... ... ... ... ... ... ... ... ... ... ... ... ... ... ... ... ... ... ... ... ... ... ... ... ... ... ...  [ 198]
TN85.417   ... ..T ... ... ... ... ... ... ... ... ... ... ... ... ... ... ... ... ... ... ... ... ... ... ... ... ... ... ... ... ... ... ...  [ 198]
TN89.356   ... ..T ... ... ... ... ... ... ... ... ... ... ... ... ... ... ... ... ... ... ... ... ... ... ... ... ... ... ... ... ... ... ...  [ 198]
TN89.1144  ... ..T ... ... ..C ... ... ... ... ... ... ... ... ... ... ... ... ... ... ... ... ... ... ... ..C ... ... ... ... ... ... ... ...  [ 198]
TN89.1259  ... ..T ... ... ..C ... ... ... ... ... ... ... ... ... ... ... ... ... ... ... ... ... ... ... ..C ... ... ... ... ... ... ... ...  [ 198]
TN89.515   ... ..T ... ... ... ... ... ... ... ... ... ... ... ... ... ... ... ... ... ... ... ... ... ... ..C ... ... ... ... ... ... ... ...  [ 198]
TN90.140   ... ..T ... ... ..C ... ... ... ... ... ... ... ... ... ... ... ... ... ... ... ... ... ... ... ..C ... ... ... ... ... ... ... ...  [ 198]
TN90.422   ... ..T ... ... ..C ... ... ... ... ... ... ... ... ... ... ... ... ... ... ... ... ... ... ... ..C ... ... ... ... ... ... ... ...  [ 198]
TN90.1031  ... ..T ... ... ... ... ... ... ... ... ... ... ... ... ... ... ... ... ... ... ... ... ... ... ... ... ... ... ... ... ... ... ...  [ 198]
TN91.316   ... ..T ... ... ... ... ... ... ... ... ... ... ... ... ... ... ... ... ... ... ... ... ... ... ... ... ... ... ... ... ... ... ...  [ 198]
```

```
TN91.320   ... ..T ..T ... ... ... ... ... ... ... ... ... ... ..T ... ... ... ... ... ... ... ... ... ... ... ... ... ... ... ... ... ... ...  [ 198]
TN91.334   ... ..T ... ... ... ... ... ... ... ... ... ... ... ... ... ... ... ... ... ... ... ... ... ... ..C ... ... ... ... ... ... ... ...  [ 198]
TN91.349   ... ..T ... ... ... ... ... ... ... ... ... ... ... ..T ... ... ... ... ... ... ... ... ... ... ... ... ... ... ... ... ... ... ...  [ 198]
TN91.451   ... ..T ... ... ... ... ... ... ... ... ... ... ... ..T ... ... ... ... ... ... ... ... ... ... ... ... ... ... ... ... ... ... ...  [ 198]
TN91.521   ... ..T ... ... ... ... ... ... ... ... ... ... ... ..T ... ... ... ... ... ... ... ... ... ... ... ... ... T.. ... ... ... ... ...  [ 198]
TN92.1031  ... ..T ... ... ... ... ... ... ... ... ... ... ... ... ... ... ... ... ... ... ... ... ... ... ... ... ... ... ... ... ... ... ...  [ 198]
TN92.1032  ... ..T ... ... ... ... ... ... ... ... ... ... ... ... ... ... ... ... ... ... ... ... ... ... ... ... ... ... ... ... ... ... ...  [ 198]
TN92.1120  ... ..T ... ... ... ... ... ... ... ... ... ... ... ... ... ... ... ... ... ... ... ... ... ... ... ... ... ... ... ... ... ... ...  [ 198]
TN93.32    ... ..T ... ... ... ... ... ... ... ... ... ... ... ... ... ... ... ... ... ... ... ... ... ... ... ... ... ... ... ... ... ... ...  [ 198]
TN93.616   ... ..T ... ... ... ... ... ... ... ... ... ... ... ... ... ... ... ... ... ... ... ... ... ... ... ... ... ... ... ... ... ... ...  [ 198]
TN94.413   ... ..T ... ... ... ... ... ... ... ... ... ... ... ... ... ... ... ... ... ... ... ... ... ... ... ... ... ... ... ... ... ... ...  [ 198]
NL94.1     ... ..T ... ... ... ... ... ... ... ... ... ... ... ... ... ... ... ... ... ... ... ... ... ... ... ... ... ... ... ... ... ... ...  [ 198]
TN95.252   ... ..T ... ... ... ... ... ... ... ... ... ... ... ... ... ... ... ... ... ... ... ... ... ... ... ... ... ... ... ..G ... ... ...  [ 198]
TN96.115   ... ..T ... ... ... ... ... ... ... ... ... ... ... ... ... ... ... ... ... ... ... ... ... ... ... ... ... ... ... ... ... ... ...  [ 198]
TN96.35    ... ..T ... ... ... ... ... ... ... ... ... ... ... ... ... ... ... ... ... ... ... ... ... ... ... ... ... ... ... ... ... ... ...  [ 198]
TN96.213   ... ..T ... ... ... ... ... ... ... ... ... ... ... ... ... ... ... ... ... ... ... ... ... ... ... ... ... ... ... ... ... ... ...  [ 198]
TN96.44    ... ..T ... ... ... ... ... ... ... ... ... ... ... ... ... ... ... ... ... ... ... ... ... ... ... ... ... ... ... ... ... ... ...  [ 198]
TN97.235   ..G ... ... ... ... ... ... ... ... ... ... ... ... ... ... ... ... ... ... ... ... ... ... ... ... ... ... ... ... ... ... ... ...  [ 198]
TN98.512   ... ..T ... ... ... ... ... ... ... ... ... ... ... ... ... ... ... ... ... ... ... ... ... ... ... ... ... ... ... ... ... ... ...  [ 198]
TN99.419   ... ..T ... ... ... ... ... ... ... ... ... ... ... ... ... ... ... ... ... ... ... ... ... ... ... ... ... ... ... ... ... ... ...  [ 198]
CAN98.73   ... ..T ... ... ... ... ... ... ... ... ... ... ... ... ... ... ... ... ... ... ... ... ... ... ... ... ... ... ... ... ... ... ...  [ 198]
CAN98.79   ... ..T ... ... ... ... ... ... ... ... ... ... ... ... ... ... ... ... ... ... ... ... ... ... ... ... ... ... ... ... ... ... ...  [ 198]
CAN00.13   ... ..T ... ... ... ... ... ... ... ... ... ... ... ... ... ... ... ... ... ... ... ... ... ... ... ... ... ... ... ... ... ... ...  [ 198]
TN01.28    ... ..T ... ... ... ... ... ... ... ... ... ... ... ... ... ... ... ... ... ... ... ... ... ... ... ... ... ... ... ... ... ... ...  [ 198]

TN82.59    ATC AAA ACA GAA CTT GAC CTA ACC AAA AGT GCT CTA AGA GAA CTC AAA ACA GTC TCT GCT GAT CAA TTA GCG AGA GAA GAA CAA ATT GAA AAT CCC AGA  [ 297]
TN83.126   ... ... ... ..G ... ... ... ... ... ... ... ... ... ... ... ... ... ... ... ... ... ... ... ..A ... ... ... ... ... ... ... ... ...  [ 297]
TN83.1211  ... ... ... ..G ... ... ... ... ... ... ... ... ... ... ... ... ... ... ... ... ... ... ... ..A ... ... ... ... ... ... ... ... ...  [ 297]
TN84.611   ... ... ... ..G ... ... ... ... ... ... ... ... ... ... ... ... ... ... ... ... ... ... ... ..A ... ... ... ... ... ... ... ... ...  [ 297]
TN85.123   ... ... ... ... ... ... ... ... ... ... ... ... ... ... ... ... ... ..T ... ... ... ..G ... ... ... ... ... ... ... ... ... ... ...  [ 297]
TN85.417   ... ... ... ... ... ... ... ... ... ... ... ... ... ... ... ... ... ..T ... ... ... ..G ... ... ... ... ... ... ... ... ... ... ...  [ 297]
TN89.356   ... ... ... ... ... ... ... ... ... ... ... ... ... ... ... ... ... ..T ... ... ... ..G ... ... ... ... ... ..G ... ... ... ... ...  [ 297]
TN89.1144  ... ... ... ... ... ... ... ... ... ... ... ... ... ... ... ... ... ..T ..C ... ... ..G ... ... ... ... ... ... ... ... ... ... ...  [ 297]
TN89.1259  ... ... ... ... ... ... ... ... ... ... ... ... ... ... ... ... ... ..T ..C ... ... ..G ... ... ... ... ... ... ... ... ... ... ...  [ 297]
TN89.515   ... ... ... ... ... ... ... ... ... ... ... ... ... ... ... ... ... ..T ..C ... ... ..G ... ... ... ... ... ... ... ... ... ... ...  [ 297]
TN90.140   ... ... ... ... ... ... ... ... ... ... ... ... ... ... ... ... ... ..T ..C ... ... ..G ... ... ... ... ... ... ... ... ... ... ...  [ 297]
TN90.422   ... ... ... ... ... ... ... ... ... ... ... ... ... ... ... ... ... ..T ..C ... ... ..G ... ... ... ... ... ... ... ... ... ... ...  [ 297]
TN90.1031  ... ... ... ... ... ... ... ... ... ... ... ... ... ... ... ... ... ..T ... ... ... ..G ... ... ... ... ... ... ... ... ... ... ...  [ 297]
TN91.316   ... ... ... ... ... ... ... ... ... ... ... ... ... ... ... ... ... ..T ... ... ... ..G ... ... ... ... ... ... ... ... ... ... ...  [ 297]
TN91.320   ... ... ... ... ... ... ... ... ... ... ... ... ... ... ... ... ... ..T ... ... ... ..G ... ..A ... ... ... ... ... ... ... ... ...  [ 297]
TN91.334   ... ... ... ... ... ... ... ... ... ... ... ... ... ... ... ... ... ..T ..C ... ... ..G ... ... ... ... ... ... ... ... ... ... ...  [ 297]
TN91.349   ... ... ... ... ... ... ... ... ... ... ... ... ... ... ... ... ... ..T ... ... ... ..G ... ..A ... ... ... ... ... ... ... ... ...  [ 297]
TN91.451   ... ... ... ... ... ... ... ... ... ... ... ... ... ... ... ... ... ..T ... ... ... ..G ... ..A ... ... ... ... ... ... ... ... ...  [ 297]
TN91.521   ... ... ... ... ... ... ... ... ... ... ... ... ... ... ... ... ... ..T ... ... ... ..G ... ..A ... ... ... ... ... ... ... ... ...  [ 297]
TN92.1031  ... ... ... ... ... ... ... ... ... ... ... ... ... ... ... ... ... ..T ... ... ... ..G ... ... ... ... ... ... ... ... ... ... ...  [ 297]
TN92.1032  ... ... ... ... ... ... ... ... ... ... ... ... ... ... ... ... ... ..T ... ... ... ..G ... ... ... ... ... ... ... ... ... ... ...  [ 297]
TN92.1120  ... ... ... ... ... ... ... ... ... ... ... ... ... ... ... ... ... ..T ... ... ... ..G ... ... ... ... ... ... ... ... ... ... ...  [ 297]
TN93.32    ... ... ... ... ... ... ... ... ... ... ... ... ... ... ... ... ... ..T ... ... ... ..G ... ... ... ... ... ... ... ... ... ... ...  [ 297]
TN93.616   ... ... ... ... ... ... ... ... ... ... ... ... ... ... ... ... ... ..T ... ... ... ..G ... ... ... ... ... ... ... ... ... ... ...  [ 297]
TN94.413   ... ... ... ... ... ... ... ... ... ... ... ... ... ... ... ... ... ..T ... ... ... ..G ... ... ... ... ... ... ... ... ... ... ...  [ 297]
NL94.1     ... ... ... ... ... ... ... ... ... ... ... ..G ... ... ... ... ... ..T ... ... ... ..G ... ... ... ... ... ... ... ... ... ... ...  [ 297]
TN95.252   ... ... ... ... ... ... ... ... ... ... ... ... ... ... ... ... ... ..T ... ... ... ..G ... ... ... ... ... ... ... ... ... ... ...  [ 297]
TN96.115   ... ... ... ... ... ... ... ... ... ... ... ... ... ... ... ... ... ..T ... ... ... ..G ... ... ... ... ... ... ... ... ... ... ...  [ 297]
```

```
TN96.35    ... ... ... ... ... ... ... ... ... ... ... ... ... ... ... ... ... ..T ... ... ... ..G ... ... ... ... ... ... ... ... ... ... ...  [ 297]
TN96.213   ... ... ... ... ... ... ... ... ... ... ... ... ... ... ... ... ... ..T ... ... ... ..G ... ... ... ... ... ... ... ... ... ... ...  [ 297]
TN96.44    ... ... ... ... ... ... ... ... ... ... ... ... ... ... ... ... ... ..T ... ... ... ..G ... ... ... ... ... ... ... ... ... ... ...  [ 297]
TN97.235   ... ... ... ... ... ... ... ... ... ... ... ... ... ... ... ... ... ... ... ... ... ... ... ..A ... ... ... ... ... ... ... ... ...  [ 297]
TN98.512   ... ... ... ... ... ... ... ... ... ... ... ... ... ... ... ... ... ..T ... ... ... ..G ... ... ... ... ..G ... ... ... ... ... ...  [ 297]
TN99.419   ... ... ... ... ... ... ... ... ... ... ... ... ... ... ... ... ... ..T ... ... ... ..G ... ... ... ... ... ... ... ..G ... ... ...  [ 297]
CAN98.73   ... ... ... ... ... ... ... ... ... ... ... ... ... ... ... ... ... ..T ... ... ... ..G ... ... ... ... ... ... ... ... ... ... ...  [ 297]
CAN98.79   ... ... ... ... ... ... ... ... ... ... ... ... ... ... ... ... ... ..T ... ... ... ..G ... ... ... ... ... ... ... ... ... ... ...  [ 297]
CAN00.13   ... ... ... ... ... ... ... ... ... ... ... ... ... ... ... ... ... ..T ... ... ... ..G ... ... ... ... ... ... ... ... ... ... ...  [ 297]
TN01.28    ... ... ... ... ... ... ... ... ... ... ... ... ... ... ... ... ... ..T ... ... ... ..G ... ... ... ... ... ... ... ... ... ... ...  [ 297]

TN82.59    CAA TCA AGG TTT GTC CTA GGT GCA ATA GCT CTT GGA GTT GCC ACA GCA GCA GCA GTC ACA GCA GGC ATT GCA ATA GCC AAA ACC ATA AGG CTT GAG AGT  [ 396]
TN83.126   ... ... ... ... ... ... ... ... ... ... ... ... ... ... ... ... ... ... ... ... ... ... ... ... ... ... ... ... ... ... ... ... ...  [ 396]
TN83.1211  ... ... ... ... ... ... ... ... ... ... ... ... ... ... ... ... ... ... ... ... ... ... ... ... ... ... ... ... ... ... ... ... ...  [ 396]
TN84.611   ... ... ... ... ... ... ... ... ... ... ... ... ... ... ... ... ... ... ... ... ... ... ... ... ... ... ... ... ... ... ... ... ...  [ 396]
TN85.123   ... ... ... ... ... ... ... ... ... ... ... ... ... ... ... ... ... ... ... ... ... ... ... ... ... ... ... ... ... ... ... ... ...  [ 396]
TN85.417   ... ... ... ... ... ... ... ... ... ... ... ... ... ... ... ... ... ... ... ... ... ... ... ... ... ... ... ... ... ... ... ... ...  [ 396]
TN89.356   ... ... ... ... ... ... ... ... ... ... ..C ... ... ... ... ... ... ... ... ... ... ... ... ... ... ... ... ... ... ... ... ... ...  [ 396]
TN89.1144  ... ... ... ... ... ... ... ... ... ... ... ... ... ... ... ... ... ... ... ... ... ... ... ... ... ... ... ... ... ... ... ... ...  [ 396]
TN89.1259  ... ... ... ... ... ... ... ... ... ... ... ... ... ... ... ... ... ... ... ... ... ... ... ... ... ... ... ... ... ... ... ... ...  [ 396]
TN89.515   ... ... ... ... ... ... ... ... ... ... ... ... ... ... ... ... ... ... ... ... ... ... ... ... ... ... ... ... ... ... ... ... ...  [ 396]
TN90.140   ... ... ... ... ... ... ... ... ... ... ... ... ... ... ... ... ... ... ... ... ... ... ... ... ... ... ... ... ... ... ... ... ...  [ 396]
TN90.422   ... ... ... ... ... ... ... ... ... ... ... ... ... ... ... ... ... ... ... ... ... ... ... ... ... ... ... ... ... ... ... ... ...  [ 396]
TN90.1031  ... ... ... ... ... ... ... ... ... ... ... ..T ... ... ... ... ... ... ... ... ... ... ... ... ... ... ... ... ... ... ... ... ...  [ 396]
TN91.316   ... ... ... ... ... ... ... ... ... ... ... ... ... ... ... ... ... ... ... ... ... ... ... ... ... ... ... ... ... ... ... ... ...  [ 396]
TN91.320   ... ... ... ... ... ... ... ... ... ... ... ... ... ... ... ... ... ... ... ... ... ... ... ... ... ... ... ... ... ... ... ... ...  [ 396]
TN91.334   ... ... ... ... ... ... ... ... ... ... ... ... ... ... ... ... ... ... ..A ... ... ... ... ... ... ... ... ... ... ... ... ... ...  [ 396]
TN91.349   ... ... ... ... ... ... ... ... ... ... ... ... ... ... ... ... ... ... ... ... ... ... ... ... ... ... ... ... ... ... ... ... ...  [ 396]
TN91.451   ... ... ... ... ... ... ... ... ... ... ... ... ... ... ... ... ... ... ... ... ... ... ... ... ... ... ... ... ... ... ... ... ...  [ 396]
TN91.521   ... ... ... ... ... ... ... ... ... ... ... ... ... ... ... ... ... ... ... ... ... ... ... ... ... ... ... ... ... ... ... ... ...  [ 396]
TN92.1031  ... ... ... ... ... ... ... ... ... ... ... ... ... ... ... ... ... ... ... ... ... ... ... ... ... ... ... ... ... ... ... ... ...  [ 396]
TN92.1032  ... ... ... ... ... ... ... ... ... ... ... ... ... ... ... ... ... ... ... ... ... ... ... ... ... ... ... ... ... ... ... ... ...  [ 396]
TN92.1120  ... ... ... ... ... ... ... ... ... ... ... ... ... ... ... ... ... ... ... ... ... ... ... ... ... ..T ... ... ... ... ... ... ...  [ 396]
TN93.32    ... ... ... ... ... ... ... ... ... ... ... ... ... ... ... ... ... ... ... ... ... ... ... ... ... ..T ... ... ... ... ... ... ...  [ 396]
TN93.616   ... ... ... ... ... ... ... ... ... ... ... ... ... ... ... ... ... ... ... ... ... ... ... ... ... ..T ... ... ... ... ... ... ...  [ 396]
TN94.413   ... ... ... ... ... ... ... ... ... ... ... ... ... ... ... ... ... ... ... ... ... ... ... ... ... ..T ... ... ... ... ... ... ...  [ 396]
NL94.1     ... ... ... ... ... ... ... ... ... ... ... ... ... ... ... ... ... ... ... ... ... ... ... ... ... ... ... ... ... ..A ... ... ...  [ 396]
TN95.252   ... ... ... ... ... ... ... ... ... ... ... ... ... ... ... ... ... ... ... ... ... ... ... ... ... ... ... ... ... ... ... ... ...  [ 396]
TN96.115   ... ... ... ... ... ... ... ... ... ... ... ... ... ... ... ... ... ... ... ... ... ... ... ... ... ... ... ... ... ... ... ... ...  [ 396]
TN96.35    ... ... ... ... ... ... ... ... ... ... ... ... ... ... ... ... ... ... ... ... ... ... ... ... ... ... ... ... ... ... ... ... ...  [ 396]
TN96.213   ... ... ... ... ... ... ... ... ... ... ... ... ... ... ... ... ... ... ... ... ... ... ... ... ... ... ... ... ... ... ... ... ...  [ 396]
TN96.44    ... ... ... ... ... ... ... ... ... ... ... ... ... ... ... ... ... ... ... ... ... ... ... ... ... ... ... ... ... ... ... ... ...  [ 396]
TN97.235   ... ... ... ... ... ... ... ... ... ... ... ... ... ... ... ... ... ... ... ... ... ... ... ... ... ..A ... ... ... ... ... ... ...  [ 396]
TN98.512   ... ... ... ... ... ... ... ... ... ... ... ... ... ... ... ... ... ... ... ... ... ... ... ... ... ... ... ... ... ... ... ... ...  [ 396]
TN99.419   ... ... ... ... ... ... ... ... ... ... ... ..T ... ... ... ... ... ... ... ..G ... ... ... ..G ... ... ... ... ... ... ... ... ...  [ 396]
CAN98.73   ... ... ... ... ... ... ... ... ... ... ... ..T ... ... ... ... ... ... ... ... ... ... ... ..G ... ... ... ... ... ... ... ... ...  [ 396]
CAN98.79   ... ... ... ... ... ... ... ... ... ... ... ..T ... ... ... ... ... ... ... ... ... ... ... ..G ... ... ... ... ... ... ... ... ...  [ 396]
CAN00.13   ... ... ... ... ... ... ... ... ... ... ... ..T ... ... ... A.. ... ... ... ... ... ... ... ..G ... ... ... ... ... ... ... ... ...  [ 396]
TN01.28    ... ... ... ... ... ... ... ... ... ... ... ... ... ... ... ... ... ... ... ... ... ... ... ... ... ... ... ... ... ... ... ... ...  [ 396]

TN82.59    GAG GTG AAT GCA ATC AAA GGT GCT CTC AAA AAA ACC AAC GAG GCA GTA TCC ACG TTA GGA AAT GGA GTG CGA GTC CTA GCC ACT GCA GTA AGA GAG CTG  [ 495]
TN83.126   ..A ... ... ... ... ... ... ... ... ... ... ... ... ... ... ... ..T ... ... ... ... ... ... ... ... ... ... ... ... ... ..G ... ...  [ 495]
TN83.1211  ..A ... ... ... ... ... ... ... ... ... ... ... ... ... ... ... ..T ... ... ... ... ... ... ... ... ... ... ... ... ... ..G ... ...  [ 495]
```

```
TN84.611    ..A ... ... ... ... ... ... ... ... ... ... ... ... ... ... ... ..T ... ... ... ... ... ... ... ... ... ... ... ... ... ..G ... ...  [ 495]
TN85.123    ..A ... ... ... ... ... ... ... ... ... ... ... ... ... ... ... ... ..A C.. ... ... ... ... ... ... ... ... ... ... ... ..G ... ...  [ 495]
TN85.417    ..A ... ... ... ... ... ... ... ... ... ... ... ... ... ... ... ... ..A C.. ... ... ... ... ... ... ... ... ... ... ... ..G ... ...  [ 495]
TN89.356    ..A ..A ... ... ... ... ... ... ... ... ... ... ... ... ... ... ... ..A C.. ... ... ... ... ... ... ... ... ... ... ... ... ... ...  [ 495]
TN89.1144   ..A ... ... ... ... ... ... ... ... ... ... ... ... ... ... ... ... ..A C.. ... ... ... ... ... ... ... ... ... ... ... ..G ... ...  [ 495]
TN89.1259   ..A ... ... ... ... ... ... ... ... ... ... ... ... ... ... ... ... ..A C.. ... ... ... ... ... ... ... ... ... ... ... ..G ... ...  [ 495]
TN89.515    ..A ... ... ... ... ... ... ... ... ... ... ... ... ... ... ... ... ..A C.. ... ... ... ... ... ... ... ... ... ... ... ..G ... ...  [ 495]
TN90.140    ..A ... ... ... ... ... ... ... ... ... ... ... ... ... ... ... ... ..A C.. ... ... ... ... ... ... ... ... ... ... ... ..G ... ...  [ 495]
TN90.422    ..A ... ... ... ... ... ... ... ... ... ... ... ... ... ... ... ... ..A C.. ... ... ... ... ... ... ... ... ... ... ... ..G ... ...  [ 495]
TN90.1031   ..A ... ... ... ... ... ... ... ... ... ... ... ... ... ... ... ... ..A C.. ... ... ... ... ... ... ... ... ... ... ... ... ... ...  [ 495]
TN91.316    ..A ... ... ... ... ... ... ... ... ... ... ... ... ... ... ... ... ..A C.. ... ... ... ... ... ... ... ... ... ... ... ... ... ...  [ 495]
TN91.320    ..A ... ... ... ..T ... ... ... ... ... ... ... ... ... ... ... ... ..A C.. ... ... ... ... ... ... ... ... ... ... ... ... ... ...  [ 495]
TN91.334    ..A ... ... ... ... ... ... ... ... ... ... ... ... ... ... ... ... ..A C.. ... ... ... ... ... ... ... ... ... ... ... ..G ... ...  [ 495]
TN91.349    ..A ... ... ... ..T ... ... ... ... ... ... ... ... ... ... ... ... ..A C.. ... ... ... ... ... ... ... ... ... ... ... ... ... ...  [ 495]
TN91.451    ..A ... ... ... ..T ... ... ... ... ... ... ... ... ... ... ... ... ..A C.. ... ... ... ... ... ... ... ... ... ... ... ... ... ...  [ 495]
TN91.521    ..A ... ... ... ..T ... ... ... ... ... ... ... ... ... ... ... ... ..A C.. ... ... ... ... ... ... ... ... ... ... ... ... ... ...  [ 495]
TN92.1031   ..A ... ... ... ... ... ... ... ... ... .C. ... ... ... ... ... ... ..A C.. ... ... ... ... ..G ... ... ... ... ... ... ... ... ...  [ 495]
TN92.1032   ..A ... ... ... ... ... ... ... ... ... .C. ... ... ... ... ... ... ..A C.. ... ... ... ... ..G ... ... ... ... ... ... ... ... ...  [ 495]
TN92.1120   ..A ... ... ... ... ... ... ... ... ... .C. ... ... ... ... ... ... ..A C.. ... ... ... ... ..G ... ... ... ... ... ... ... ... ...  [ 495]
TN93.32     ..A ... ... ... ... ... ... ... ... ... .C. ... ... ... ... ... ... ..A C.. ... ... ... ... ..G ... ... ... ... ... ... ... ... ...  [ 495]
TN93.616    ..A ... ... ... ... ... ... ... ... ... .C. ... ... ... ... ... ... ..A C.. ... ... ... ... ..G ... ... ... ... ... ... ... ... ...  [ 495]
TN94.413    ..A ... ... ... ... ... ... ... ... ... .C. ... ... ... ... ... ... ..A C.. ... ... ... ... ..G ... ... ... ... ... ... ... ... ...  [ 495]
NL94.1      ..A ... ... ... ... ... ... ... ... ... .C. ... ... ... ... ... ... ..A C.. ... ... ... ... ... ... ... ... ... ... ... ... ... ...  [ 495]
TN95.252    ..A ... ... ... ... ... ... ... ... ... .C. ... ... ... ... ... ... ..A C.. ... ... ... ... ..G ... ... ... ... ... ... ... ... ...  [ 495]
TN96.115    ..A ... ... ... ... ... ... ... ... ... .C. ... ... ... ... ... ... ..A C.. ... ... ... ... ..G ... ... ... ... ... ... ... ... ...  [ 495]
TN96.35     ..A ... ... ... ... ... ... ... ... ... .C. ... ... ... ... ... ... ..A C.. ... ... ... ... ..G ... ... ... ... ... ... ... ... ...  [ 495]
TN96.213    ..A ... ... ... ... ... ... ... ... ... .C. ... ... ... ... ... ... ..A C.. ... ... ... ... ..G ... ... ... ... ... ... ... ... ...  [ 495]
TN96.44     ..A ... ... ... ... ... ... ... ... ... .C. ... ... ... ... ... ... ..A C.. ... ... ... ... ..G ... ... ... ... ... ... ... ... ...  [ 495]
TN97.235    ..A ... ... ... ... ... ... ... ... ... ... ... ... ... ... ... ..T ... ... ... ... ... ... ... ... ... ... ... ... ... ..G ... ...  [ 495]
TN98.512    ..A ... ... ... ... ... ... ... ... ... ... ... ... ... ... ... ... ..A C.. ... ... ... ... ... ... ... ... ... ... ... ... ... ...  [ 495]
TN99.419    ..A ... ... ... ... ... ... ... ... ... .C. ... ..N ... ... ... ... ..A C.N ... ..N ... ... N.. ... ... ... ..N ... ... ... ... N..  [ 495]
CAN98.73    ..A ... ... ... ... ... ... ... ... ... .C. ... ... ... ... ... ... ..A C.. ... ... ... ... ... ... ... ... ..C ... ... ... ... ...  [ 495]
CAN98.79    ..A ... ... ... ... ... .A. ... ... ... .C. ... ... ... ... ... ... ..A C.. ... ... ... ... ... ... ... ... ..C ... ... ... ... ...  [ 495]
CAN00.13    ..A ... ... ... ... ... ... ... ... ... .C. ... ... ... ... ... ... ..A C.. ... ... ... ... ... ... ... ... ..C ... ... ... ... ...  [ 495]
TN01.28     ..A ... ... ... ... ... ... ... ... ... .C. ... ... ... ... ... ... ..A C.. ... ... ... ... ... ... ... ... ... ... ... ... ... ...  [ 495]

TN82.59     AAA GAA TTT GTG AGC AAA AAC CTG ACT AGT GCA ATC AAC AAG AAC AAA TGT GAC ATT GCT GAT CTG AAG ATG GCT GTC AGC TTC AGT CAA TTC AAC AGA  [ 594]
TN83.126    ... ... ... ... ... ... ... ... ... ... ... ... ... ... ... ... ... ... ... ... ... ... ... ... ... ... ... ... ... ... ... ... ...  [ 594]
TN83.1211   ... ... ... ... ... ... ... ... ... ... ... ... ... ... ... ... ... ... ... ... ... ... ... ... ... ... ... ... ... ... ... ... ...  [ 594]
TN84.611    ... ... ... ... ... ... ... ... ... ... ... ... ... ... ... ... ... ... ... ... ... ... ... ... ... ... ... ... ... ... ... ... ...  [ 594]
TN85.123    ... ... ... ... ... ... ... ... ... ... ..G ... ... ... ... ... ... ... ... ... ... ... ... ... ... ... ... ... ... ... ... ... ...  [ 594]
TN85.417    ... ... ... ... ... ... ... T.. ... ... ..G ... ... ... ... ... ... ... ... ... ... ... ... ... ... ... ... ... ... ... ... ... ...  [ 594]
TN89.356    ... ... ... ... ... ... ... ... ... ... ..G ... ... ... ... ... ... ... ... ... ... ... ... ... ... ... ... ... ... ... ... ... ...  [ 594]
TN89.1144   ... ... ... ... ... ... ... ... ... ... ..G ... ... ... ... ... ... ... ... ... ... ... ... ... ... ... ... ... ... ... ... ... ...  [ 594]
TN89.1259   ... ... ... ... ... ... ... ... ... ... ..G ... ... ... ... ... ... ... ... ... ... ... ... ... ... ... ... ... ... ... ... ... ...  [ 594]
TN89.515    ... ... ... ... ... ... ... ... ... ... ..G ... ... ... ... ... ... ... ... ... ... ... ... ... ... ... ... ... ... ... ... ... ...  [ 594]
TN90.140    ... ... ... ... ... ... ... ... ... ... ..G ... ... ... ... ... ... ... ... ... ... ... ... ... ... ... ... ... ... ... ... ... ...  [ 594]
TN90.422    ... ... ... ... ... ... ... ... ... ... ..G ... ... ... ... ... ... ... ... ... ... ... ... ... ... ... ... ... ... ... ... ... ...  [ 594]
TN90.1031   ... ... ... ... ... ... ... ... ... ... ..G ... ... ... ... ... ... ... ... ... ... ... ... ... ... ... ... ... ... ... ... ... ...  [ 594]
TN91.316    ... ... ... ... ... ... ... ... ... ... ..G ... ... ... ... ... ... ... ... ... ... ... ... ... ... ... ... ... ... ... ... ... ...  [ 594]
TN91.320    ... ... ... ... ... ... ... ... ... ... ..G ... ... ... ... ... ... ... ... ... ... ... ... ... ... ... ... ... ... ... ... ... ...  [ 594]
TN91.334    ... ... ... ... ... ... ... ... ... ... ..G ... ... ... ... ... ... ... ... ... ... ... ... ... ... ... ... ... ... ... ... ... ...  [ 594]
TN91.349    ... ... ... ... ... ... ... ... ... ... ..G ... ... ... ... ... ... ... ... ... ... ... ... ... ... ... ... ... ... ... ... ... ...  [ 594]
```

```
TN91.451   ... ... ... ... ... ... ... ... ... ... ..G ... ... ... ... ... ... ... ... ... ... ... ... ... ... ... ... ... ... ... ... ... ...  [ 594]
TN91.521   ... ... ... ... ... ... ... ... ... ... ..G ... ... ... ... ... ... ... ... ... ... ... ... ... ... ... ... ... ... ... ... ... ...  [ 594]
TN92.1031  ... ... ... ... ... ... ... ... ... ... ..G ... ... ... ... ... ... ... ... ... ... ... ... ... ... ... ... ... ... ..G ... ... ...  [ 594]
TN92.1032  ... ... ... ... ... ... ... ... ... ... ..G ... ... ... ... ... ... ... ... ... ... ... ... ... ... ... ... ... ... ..G ... ... ...  [ 594]
TN92.1120  ... ... ... ... ... ... ... ... ... ... ..G ... ... ... ... ... ... ... ... ... ... ... ... ... ... ... ... ... ... ..G ... ... ...  [ 594]
TN93.32    ... ... ... ... ... ... ... ... ... ... ..G ... ... ... ... ... ... ... ... ... ... ... ... ... ... ... ... ... ... ..G ... ... ...  [ 594]
TN93.616   ... ... ... ... ... ... ... ... ... ... ..G ... ... ... ... ... ... ... ... ... ... ... ... ... ... ... ... ... ... ..G ... ... ...  [ 594]
TN94.413   ... ... ... ... ... ... ... ... ... ... ..G ... ... ... ... ... ... ... ... ... ... ... ... ... ... ... ... ... ... ..G ... ... ...  [ 594]
NL94.1     ... ... ... ... ... ... ... ... ... ... ..G ... ... ... ... ... ... ... ... ... ... ... ... ... ... ... ... ... ... ... ... ... ...  [ 594]
TN95.252   ..G ... ... ... ... ... ... ... ... ... ..G ... ... ... ... ... ... ... ... ... ... ... ... ... ... ... ... ... ... ..G ... ... ...  [ 594]
TN96.115   ... ... ... ... ... ... ... ... ... ... ..G ... ... ... ... ... ... ... ... ... ... ... ... ... ... ... ... ... ... ..G ... ... ...  [ 594]
TN96.35    ... ... ... ... ... ... ... ... ... ... ..G ... ... ... ... ... ... ... ... ... ... ... ... ... ... ... ... ... ... ..G ... ... ...  [ 594]
TN96.213   ... ... ... ... ... ... ... ... ... ... ..G ... ... ... ... ... ... ... ... ... ... ... ... ... ... ... ... ... ... ..G ... ... ...  [ 594]
TN96.44    ... ... ... ... ... ... ... ... ... ... ..G ... ... ... ... ... ... ... ... ... ... ... ... ... ... ... ... ... ... ..G ... ... ...  [ 594]
TN97.235   ... ... ... ... ... ... ... ... ... ... ... ... ... ... ... ... ... ... ... ... ... ... ... ... ... ... ... ... ... ... .A. ... ...  [ 594]
TN98.512   ... ... ... ... ... ... ... ... ... ... ..G ... ... ... ... ... ... ... ... ... ... ... ... ... ... ... ... ... ... ... ... ... ...  [ 594]
TN99.419   ... ... ... ... ... ... ... N.. .N. ... ... ..T ... ... ... ... ... ... .N. .N. ... ... ... ... N.. ... ... ... ... ... ... ... ...  [ 594]
CAN98.73   ... ... ... ... ... ... ... ... ... ... ... ..T ... ... ... ... ... ... ... ... ... ... ... ... ... ... ... ... ... ... ... ... ...  [ 594]
CAN98.79   ... ... ... ... ... ... ... ... ... ... ... ..T ... ... ... ... ... ... ... ... ... ... ... ... ... ... ... ... ... ... ... ... ...  [ 594]
CAN00.13   ... ... ... ... ... ... ... ... ... ... ... ..T ... ... ... ... ... ... ... ... ... ... ... ... ... ... ... ... ... ... ... ... ...  [ 594]
TN01.28    ... ... ... ... ... ... ... ... ... ... ..G ... ... ... ... ... ... ... ... ... ... ... ... ... ... ... ... ... ... ... ... ... ...  [ 594]

TN82.59    AGA TTC CTA AAT GTT GTG CGG CAG TTT TCA GAC AAT GCA GGG ATA ACA CCA GCA ATA TCA TTG GAC CTA ATG ACT GAT GCT GAG TTG GCC AGA GCC GTA  [ 693]
TN83.126   ... ... T.. ... ... ... ... ... ... ... ... ... ... ... ... ... ... ... ... ... ... ... ... ... ... ... ... ... ... ... ... ..T ..G  [ 693]
TN83.1211  ... ... T.. ... ... ... ... ... ... ... ... ... ... ... ... ... ... ... ... ... ... ... ... ... ... ... ... ... ... ... ... ..T ..G  [ 693]
TN84.611   ... ... T.. ... ... ... ... ... ... ... ... ... ... ... ... ... ... ... ... ... ... ... ... ... ... ... ... ... ... ... ... ..T ..G  [ 693]
TN85.123   ... ... ... ... ... ... ... ... ... ... ... ... ... ... ... ... ... ... ... ... ... ... ... ... ... ... ... ... ... ... ... ..T ...  [ 693]
TN85.417   ... ... ... ... ... ... ... ... ... ... ... ... ... ... ... ... ... ... ... ... ... ... ... ... ... ... ... ... ... ... ... ..T ...  [ 693]
TN89.356   ... ... ... ... ... ... ... ... ... ... ... ... ... ... ... ... ... ... ... ... ... ... ... ... ... ... ... ... C.. ... ... ..T ...  [ 693]
TN89.1144  ... ... ... ... ... ... ... ... ... ... ... ... ... ... ... ... ... ... ... ... ... ... ... ... ... ... ... ... ... ... ... ..T ...  [ 693]
TN89.1259  ... ... ... ... ... ... ... ... ... ... ... ... ... ... ... ... ... ... ... ... ... ... ... ... ... ... ... ... ... ... ... ..T ...  [ 693]
TN89.515   ... ... ... ... ... ... ... ... ... ... ... ... ... ... ... ... ... ... ... ... ... ... ... ... ... ... ... ... ... ... ... ..T ...  [ 693]
TN90.140   ... ... ... ... ... ... ... ... ... ... ... ... ... ... ... ... ... ... ... ... ... ... ... ... ... ... ... ... ... ... ... ..T ...  [ 693]
TN90.422   ... ... ... ... ... ... ... ... ... ... ... ... ... ... ... ... ... ... ... ... ... ... ... ... ... ... ... ... ... ... ... ..T ...  [ 693]
TN90.1031  ... ... ... ... ... ... ... ... ... ... ... ... ... ... ... ... ... ... ... ... ... ... ... ... ... ... ... ... C.. ... ... ..T ...  [ 693]
TN91.316   ... ... ... ... ... ... ... ... ... ... ... ... ... ... ... ... ... ... ... ... ... ... ... ... ... ... ... ... C.. ... ... ..T ...  [ 693]
TN91.320   ... ... ... ... ... ... ... ... ... ... ... ... ... ... ... ... ... ... ... ... ... ... ... ... ... ... ... ... C.. ... ... ..T ...  [ 693]
TN91.334   ... ... ... ... ... ... ... ... ... ... ... ... ... ... ... ... ... ... ... ... ... ... ... ... ... ... ... ... ... ... ... ..T ...  [ 693]
TN91.349   ... ... ... ... ... ... ... ... ... ... ... ... ... ... ... ... ... ... ... ... ... ... ... ... ... ... ... ... ... ... ... ..T ...  [ 693]
TN91.451   ... ... ... ... ... ... ... ... ... ... ... ... ... ... ... ... ... ... ... ... ... ... ... ... ... ... ... ... C.. ... ... ..T ...  [ 693]
TN91.521   ... ... ... ... ... ... ... ... ... ... ... ... ... ... ... ... ... ... ... ... ... ... ... ... ... ... ... ... C.. ... ... ..T ...  [ 693]
TN92.1031  ... ... ... ... ... ... ... ... ... ... ... ... ... ... ... ... ... ... ... ... ... ... ... ... ... ... ... ... C.. ... ... ..T ...  [ 693]
TN92.1032  ... ... ... ... ... ... ... ... ... ... ... ... ... ... ... ... ... ... ... ... ... ... ... ... ... ... ... ... C.. ... ... ..T ...  [ 693]
TN92.1120  ... ... ... ... ... ... ... ... ... ... ... ... ... ... ... ... ... ... ... ... ... ... ... ... ... ... ... ... C.. ... ... ..T ...  [ 693]
TN93.32    ... ... ... ... ... ... ... ... ... ... ... ... ... ... ... ... ... ... ... ... ... ... ... ... ... ... ... ... C.. ... ... ..T ...  [ 693]
TN93.616   ... ... ... ... ... ... ... ... ... ... ... ... ... ... ... ... ... ... ... ... ... ... ... ... ... ... ... ... C.. ... ... ..T ...  [ 693]
TN94.413   ... ... ... ... ... ... ... ... ... ... ... ... ... ... ... ... ... ... ... ... ... ... ... ... ... ... ... ... C.. ... ... ..T ...  [ 693]
NL94.1     ... ... ... ... ... ... ... ... ... ... ... ... ... ... ... ... ... ... ... ... ... ... ... ... ... ... ... ... C.. ... ... ..T ...  [ 693]
TN95.252   ... ..T ... ... ... ... ... ... ... ... ... ... ... ... ... ... ... ... ... ... ... ... ... ... ... ... ... ... C.. ... ... ..T ...  [ 693]
TN96.115   ... ... ... ... ... ... ... ... ... ... ... ... ... ... ... ... ... ... ... ... ... ... ... ... ... ... ... ... C.. ... ... ..T ...  [ 693]
TN96.35    ... ... ... ... ... ... ... ... ... ... ... ... ... ... ... ... ... ... ... ... ... ... ... ... ... ... ... ... C.. ... ... ..T ...  [ 693]
TN96.213   ... ... T.. ... ... ... ... ... ... ... ... ... ... ... ... ... ... ... ... ... ... ... ... ... ... ... ... ... C.. ... ... ..T ...  [ 693]
TN96.44    ... ... ... ... ... ... ... ... ... ... ... ... ... ... ... ... ... ... ... ... ... ... ... ... ... ... ... ... C.. ... ... ..T ...  [ 693]
```

```
TN97.235    ... ... T.. ... ... ... ... ... ... ... ... ... ... ... ... ... ... ... ... ... ... ... ... ... ... ... ... ..A ... ... ... ..T ...  [ 693]
TN98.512    ... ... ... ... ... ... ... ... ... ... ... ... ... ... ... ... ... ... ... ... ... ... ... ... ..C ... ... ... C.. ... ... ..T ...  [ 693]
TN99.419    ... ... ... ... ... ... ... ... ... ... ... ... ... ... ... ... ... ... ... ... ... ... ... ... ... ... ... ... C.. ... ... ..T ...  [ 693]
CAN98.73    ... ... ... ... ... ... ... ... ... ... ... ... ... ... ... ... ... ... ... ... ... ... ... ... ... ... ... ... C.. ... ... ..T ...  [ 693]
CAN98.79    ... ... ... ... ... ... ... ... ... ... ... ... ... ... ... ... ... ... ... ... ... ... ... ... ... ... ... ... C.. ... ... ..T ...  [ 693]
CAN00.13    ... ... ... ... ... ... ... ... ... ... ... ... ... ... ... ... ... ... ... ... ..A ... ... ... ... ... ... ... C.. ... ... ..T ...  [ 693]
TN01.28     ... ... ... ... ... ... ... ... ... ... ... ... ... ... ... ... ... ... ... ... ... ... ... ... ..C ... ... ... C.. ... ... ..T ...  [ 693]

TN82.59     TCA TAC ATG CCA ACA TCT GCA GGA CAG ATA AAA CTA ATG TTA GAG AAC CGC GCA ATG GTG AGG AGG AAA GGA TTT GGA ATC TTG ATA GGG GTC TAC GGA  [ 792]
TN83.126    ... ... ... ... ... ... ... ... ... ... ... ... ... ... ... ... ... ... ... ... ... ..A ... ... ... ... ... ... ... ... ... ... ...  [ 792]
TN83.1211   ... ... ... ... ... ... ... ... ... ... ... ... ... ... ... ... ... ... ... ... ... ..A ... ... ... ... ... ... ... ... ... ... ...  [ 792]
TN84.611    ... ... ... ... ... ... ... ... ... ... ... ... ... ... ... ... ... ... ... ... ... ..A ... ... ... ... ... ... ... ... ... ... ...  [ 792]
TN85.123    ... ... ... ... ... ... ... ... ... ... ... ... ... ... ... ... ..T ... ... ... ... ..A ... ... ... ... ... ... ... ... ... ... ...  [ 792]
TN85.417    ... ... ... ... ... ... ... ... ... ... ... ... ... ... ... ... ..T ... ... ... ... ..A ... ... ... ... ... ... ... ... ... ... ...  [ 792]
TN89.356    ... ... ... ... ... ... ... ... ... ... ... ... ... ... ... ... ..T ... ... ... ... ..A ... ... ... ... ... ... ... ... ... ... ...  [ 792]
TN89.1144   ... ... ... ... ... ... ... ... ... ... ... ... ... ... ... ... ..T ... ... ... ... ..A ... ... ... ... ... ... ... ... ... ... ...  [ 792]
TN89.1259   ... ... ... ... ... ... ... ... ... ... ... ... ... ... ... ... ..T ... ... ... ... ..A ... ... ... ... ... ... ... ... ... ... ...  [ 792]
TN89.515    ... ... ... ... ... ... ... ... ... ... ... ... ... ... ... ... ..T ... ... ... ... ..A ... ... ... ... ... ... ... ... ... ... ...  [ 792]
TN90.140    ... ... ... ... ... ... ... ... ... ... ... ... ... ... ... ... ..T ... ... ... ... ..A ... ... ... ... ... ... ... ... ... ... ...  [ 792]
TN90.422    ... ... ... ... ... ... ... ... ... ... ... ... ... ... ... ... ..T ... ... ... ... ..A ... ... ... ... ... ... ... ... ... ... ...  [ 792]
TN90.1031   ... ... ... ... ... ... ... ... ... ... ... ... ... ... ... ... ..T ... ... ... ... ..A ... ... ... ... ... ... ... ... ... ... ...  [ 792]
TN91.316    ... ... ... ... ... ... ... ... ... ... ... ... ... ... ... ... ..T ... ... ... ... ..A ... ... ... ... ... ... ... ... ... ... ...  [ 792]
TN91.320    ... ... ... ... ... ... ... ... ... ... ... ... ... ... ... ... ..T ... ... ... ... ..A ... ... ... ... ... ... ... ... ... ... ...  [ 792]
TN91.334    ... ... ... ... ... ... ... ... ... ... ... ... ... ... ... ... ..T ... ... ... ... ..A ... ... ... ... ... ... ... ... ... ... ...  [ 792]
TN91.349    ... ... ... ... ... ... ... ... ... ... ... ... ... ... ... ... ..T ... ... ... ... ..A ... ... ... ... ... ... ... ... ... ... ...  [ 792]
TN91.451    ... ... ... ... ... ... ... ... ... ... ... ... ... ... ... ... ..T ... ... ... ... ..A ... ... ... ... ... ... ... ... ... ... ...  [ 792]
TN91.521    ... ... ... ... ... ... ... ... ... ... ... ... ... ... ... ... ..T ... ... ... ... ..A ... ... ... ... ... ... ... ... ... ... ...  [ 792]
TN92.1031   ... ... ... ... ... ... ... ... ... ... ... ... ... ... ... ... ..T ... ... ... ... ..A ... ... ... ... ... ... ... ... ... ... ...  [ 792]
TN92.1032   ... ... ... ... ... ... ... ... ... ... ... ... ... ... ... ... ..T ... ... ... ... ..A ... ... ... ... ... ... ... ... ... ... ...  [ 792]
TN92.1120   ... ... ... ... ... ... ... ... ... ... ... ... ... ... ... ... ..T ... ... ... ... ..A ... ... ... ... ... ... ... ... ... ... ...  [ 792]
TN93.32     ... ... ... ... ... ... ... ... ... ... ... ... ... ... ... ... ..T ... ... ... ... ..A ... ... ... ... ... ... ... ... ... ... ...  [ 792]
TN93.616    ... ... ... ... ... ... ... ... ... ... ... ... ... ... ... ... ..T ... ... ... ... ..A ... ... ... ... ... ... ... ... ... ... ...  [ 792]
TN94.413    ... ... ... ... ... ... ... ... ... ... ... ... ... ... ... ... ..T ... ... ... ... ..A ... ... ... ... ... ... ... ... ... ... ...  [ 792]
NL94.1      ... ... ... ... ... ... ... ... ... ... ... ... ... ... ... ... ..T ... ... ... ... ..A ... ... ... ... ... ... ... ... ... ... ...  [ 792]
TN95.252    ... ... ... ... ... ... ... ... ... ... ... ... ... ... ... ... ..T ... ... ... ... ..A ... ... ... ... ... ... ... ..A ... ... ...  [ 792]
TN96.115    ... ... ... ... ... ... ... ... ... ... ... ... ... ... ... ... ..T ... ... ... ... ..A ... ... ... ... ... ... ... ... ... ... ...  [ 792]
TN96.35     ... ... ... ... ... ... ... ... ... ... ... ... ... ... ... ... ..T ... ... ... ... ..A ... ... ... ... ... ... ... ... ... ... ...  [ 792]
TN96.213    ... ... ... ... ... ... ... ... ... ... ... ... ... ... ... ... ..T ... ... ... ... ..A ... ... ... ... ... ... ... ... ... ... ...  [ 792]
TN96.44     ... ... ... ... ... ... ... ... ... ... ... ... ... ... ... ... ..T ... ... ... ... ..A ... ... ... ... ... ... ... ... ... ... ...  [ 792]
TN97.235    ... ... ... ... ... ... ... ... ... ... ... ... ... ... ... ... ... ... ... ... ... ..A ... ... ... ... ..A ... ... ... ... ... ...  [ 792]
TN98.512    ... ... ... ... ... ... ... ... ... ... ... ... ... ... ... ... ..T ... ... ... ... ..A ... ... ... ... ... ... ... ... ... ... ...  [ 792]
TN99.419    ... ... ... ... ... ... ... ... ... ... ... ... ... ... ... ... ..T ... ... ... ... ..A ... ... ... ... ... ... ... ... ... ... ...  [ 792]
CAN98.73    ... ... ... ... ... ... ... ... ... ... ... ... ... ... ... ... ..T ... ... ... ... ..A ... ... ... ... ... ... ... ... ... ... ...  [ 792]
CAN98.79    ... ... ... ... ... ... ... ... ... ... ... ... ... ... ... ... ..T ... ... ... ... ..A ... ... ... ... ... ... ... ... ... ... ...  [ 792]
CAN00.13    ... ... ... ... ... ... ... ... ... ... ... ... ... ... ... ... ..T ... ... ... ... ..A ... ..T ... ... ... ... ... ... ... ... ...  [ 792]
TN01.28     ... ... ... ... ... ... ... ... ... ... ... ... ... ... ... ... ..T ... ... ... ... ..A ... ... ... ... ... ... ... ... ... ... ...  [ 792]

TN82.59     AGC TCT GTG ATT TAC ATG GTT CAG TTG CCG ATC TTT GGT GTC ATA GAT ACG CCT TGT TGG ATA ATT AAG GCA GCT CCC TCT TGT TCA GAA AAA GAC GGA  [ 891]
TN83.126    ... ... ... ... ... ... ... ... ... ... ... ... ... ... ... ... ..A ... ... ... ... ..C ... ... ... ... ... ... ... ... ... ..T ...  [ 891]
TN83.1211   ... ... ... ... ... ... ... ... ... ... ... ... ... ... ... ... ..A ... ... ... ... ..C ... ... ... ... ... ... ... ... ... ..T ...  [ 891]
TN84.611    ... ... ... ... ... ... ... ... ... ... ... ... ... ... ... ... ..A ... ... ... ... ..C ... ... ... ... ... ... ... ... ... ..T ...  [ 891]
TN85.123    ... ... ... ... ... ... ..C ... ... ... ... ... ... ... ... ... ..A ... ... ... ... ..C ... ... ... ... ... ... ... ... ... ..T ...  [ 891]
TN85.417    ... ... ... ... ... ... ..C ... ... ... ... ... ... ... ... ... ..A ..C ... ... ... ..C ... ... ... ... ... ... ... ... ... ..T ...  [ 891]
```

```
TN89.356   ... ... ... ... ... ... ..C ... C.. ... ... ... ... ... ... ... ..A ... ... ... ... ..C ... ... ... ... ... ... ... ... ... ..T ...  [ 891]
TN89.1144  ... ... ... ... ... ... ..C ... C.. ... ... ... ... ... ... ... ..A ... ... ... ... ..C ... ... ... ... ... ... ... ... ... ..T ...  [ 891]
TN89.1259  ... ... ... ... ... ... ..C ... C.. ... ... ... ... ... ... ... ..A ... ... ... ... ..C ... ... ... ... ... ... ... ... ... ..T ...  [ 891]
TN89.515   ... ... ... ... ... ... ..C ... C.. ... ..T ... ... ... ... ... ..A ... ... ... ... ..C ... ... ... ... ... ... ... ... ... ..T ...  [ 891]
TN90.140   ... ... ... ... ... ... ..C ... C.. ... ... ... ... ... ... ... ..A ... ... ... ... ..C ... ... ... ... ... ... ... ... ... ..T ...  [ 891]
TN90.422   ... ... ... ... ... ... ..C ... C.. ... ... ... ... ... ... ... ..A ... ... ... ... ..C ... ... ... ... ... ... ... ... ... ..T ...  [ 891]
TN90.1031  ... ... ... ... ... ... ..C ... C.. ... ... ... ... ... ... ... ..A ... ... ... ... ..C ... ... ... ... ... ... ... ... ... ..T ...  [ 891]
TN91.316   ... ... ... ... ... ... ..C ... C.. ... ... ... ... ... ... ... ..A ... ... ... ... ..C ... ... ... ... ... ... ... ... ... ..T ...  [ 891]
TN91.320   ... ... ... ... ... ... ..C ... C.. ... ... ... ... ... ... ... ..A ... ... ... ... ..C ... ... ... ... ... ... ... ... ... ..T ...  [ 891]
TN91.334   ... ... ... ... ... ... ..C ... C.. ... ... ... ... ... ... ... ..A ... ... ... ... ..C ... ... ... ... ... ... ... ... ... ..T ...  [ 891]
TN91.349   ... ... ... ... ... ... ..C ... C.. ... ... ... ... ... ... ... ..A ... ... ... ... ..C ... ... ... ... ... ... ... ... ... ..T ...  [ 891]
TN91.451   ... ... ... ... ... ... ..C ... C.. ... ... ... ... ... ... ... ..A ... ... ... ... ..C ... ... ... ... ... ... ... ... ... ..T ...  [ 891]
TN91.521   ... ... ... ... ... ... ..C ... C.. ... ... ... ... ... ... ... ..A ... ... ... ... ..C ... ... ... ... ... ... ... ... ... ..T ...  [ 891]
TN92.1031  ... ... ... ... ... ... ..C ... C.. ... ... ... ... ... ... ... ..A ... ... ... ... ..C ... ... ... ... ... ... ... ... ... ..T ...  [ 891]
TN92.1032  ... ... ... ... ... ... ..C ... C.. ... ... ... ... ... ... ... ..A ... ... ... ... ..C ... ... ... ... ... ... ... ... ... ..T ...  [ 891]
TN92.1120  ... ... ... ... ... ... ..C ... C.. ... ... ... ... ... ... ... ..A ... ... ... ... ..C ..A ... ... ... ... ... ... ... ... ..T ...  [ 891]
TN93.32    ... ... ... ... ... ... ..C ... C.. ... ... ... ... ... ... ... ..A ... ... ... ... ..C ..A ... ... ... ... ... ... ... ... ..T ...  [ 891]
TN93.616   ... ... ... ... ... ... ..C ... C.. ... ... ... ... ... ... ... ..A ... ... ... ... ..C ..A ... ... ... ... ... ... ... ... ..T ...  [ 891]
TN94.413   ... ... ... ... ... ... ..C ... C.. ... ... ... ... ... ... ... ..A ... ... ... ... ..C ... ... ... ... ... ... ... ... ... ..T ...  [ 891]
NL94.1     ... ... ... ... ... ... ..C ... C.. ... ... ... ... ... ... ... ..A ... ... ... ... ..C ... ... ... ... ... ... ... ... ... ..T ...  [ 891]
TN95.252   ... ... ... ... ... ... ..C ... C.. ... ... ... ... ... ... ... ..A ... ... ... ... ..C ... ... ..C ... ... ... ... ... ... ..T ...  [ 891]
TN96.115   ... ... ... ... ... ... ..C ... C.. ... ... ... ... ... ... ... ..A ... ... ... ... ..C ... ... ..C ... ... ... ... ... ... ..T ...  [ 891]
TN96.35    ... ... ... ... ... ... ..C ... C.. ... ... ... ... ... ... ... ..A ... ... ... ... ..C ... ... ..C ... ... ... ... ... ... ..T ...  [ 891]
TN96.213   ... ... ... ... ... ... ..C ... C.. ... ... ... ... ... ... ... ..A ... ... ... ... ..C ... ... ..C ... ... ... ... ... ... ..T ...  [ 891]
TN96.44    ... ... ... ... ... ... ..C ... C.. ... ... ... ... ... ... ... ..A ... ... ... ... ..C ... ... ..C ... ... ... ... ... ... ..T ...  [ 891]
TN97.235   ... ... ... ... ... ... ... ... ... ... ... ... ... ... ... ... ..A ... ... ... ... ..C ... ... ... ... ... ... ... ... ... ..T ...  [ 891]
TN98.512   ... ... ... ... ... ... ..C ... C.. ... ... ... ... ... ... ... ..A ... ... ... ... ..C ... ... ... ... ... ... ... ... ... ..T ...  [ 891]
TN99.419   ... ..C ... ... ... ... ..C ... C.. ... ... ... ... ... ... ... ..A ... ... ... ... ..C ... ... ... ... ... ... ... ... ... ..T ...  [ 891]
CAN98.73   ... ..C ... ... ... ... ..C ... C.. ... ... ... ... ... ... ... ..A ... ... ... ... ..C ... ... ... ... ... ... ... ... ... ..T ...  [ 891]
CAN98.79   ... ..C ... ... ... ... ..C ... C.. ... ... ... ... ... ... ... ..A ... ... ... ... ..C ... ... ... ... ... ... ... ... ... ..T ...  [ 891]
CAN00.13   ... ..C ... ... ... ... ..C ... C.. ... ... ... ... ... ... ... ..A ... ... ... ... ..C ... ... ... ... ... ... ... ... ... ..T ...  [ 891]
TN01.28    ... ... ... ... ... ... ..C ... C.. ... ... ... ... ... ... ... ..A ... ... ... ... ..C ... ... ... ... ... ... ... ... ... ..T ...  [ 891]

TN82.59    AAC TAT GCC TGC CTC CTA AGA GAG GAT CAA GGG TGG TAT TGT AAA AAT GCA GGA TCC ACT GTT TAC TAC CCA AAT GAA AAA GAC TGC GAA ACA AGA GGT  [ 990]
TN83.126   ... ... ..T ... ... ... ... ... ... ... ... ... ... ... ... ... ... ... ... ... ... ... ... ... ..C ... ... ... ... ... ... ... ...  [ 990]
TN83.1211  ... ... ..T ... ... ... ... ... ... ... ... ... ... ... ... ... ... ... ... ... ... ... ... ... ..C ... ... ... ... ... ... ... ...  [ 990]
TN84.611   ... ... ..T ... ... ... ... ... ... ... ... ... ... ... ... ... ... ... ... ... ... ... ... ... ..C ... ... ... ... ... ... ... ...  [ 990]
TN85.123   ..T ... ..T ... ... ... ... ... ... ... ... ... ... ... ... ... ... ... ... ... ... ... ... ... ... ... ... ... ... ... ... ... ...  [ 990]
TN85.417   ..T ... ..T ... ..T ... ... ... ... ... ... ... ... ... ... ... ... ... ... ... ... ... ... ... ... ... ... ... ... ... ... ... ...  [ 990]
TN89.356   ..T ... ..T ... ... ... ... ... ... ... ... ... ... ... ... ... ... ... ... ... ... ... ... ... ... ... ... ... ... ... ... ... ...  [ 990]
TN89.1144  ..T ... ..T ... ... ... ... ... ... ... ... ... ... ... ... ... ... ... ... ... ... ... ... ... ... ... ... ... ... ... ... ... ...  [ 990]
TN89.1259  ..T ... ..T ... ... ... ... ... ... ... ... ... ... ... ... ... ... ... ... ... ... ... ... ... ... ... ... ... ... ... ... ... ...  [ 990]
TN89.515   ..T ... ..T ... ... T.. ... ... ... ... ... ... ... ... ... ... ... ... ... ... ... ... ... ... ... ... ... ... ... ... ... ... ...  [ 990]
TN90.140   ..T ... ..T ... ... ... ... ... ... ... ... ... ... ... ... ... ... ... ... ... ... ... ... ... ... ... ... ... ... ... ... ... ...  [ 990]
TN90.422   ..T ... ..T ... ... ... ... ... ... ... ... ... ... ... ... ... ... ... ... ... ... ... ... ... ... ... ... ... ... ... ... ... ...  [ 990]
TN90.1031  ..T ... ..T ... ... ... ... ... ... ... ... ... ... ... ... ... ... ... ... ... ... ... ... ... ... ... ... ... ... ... ... ... ...  [ 990]
TN91.316   ..T ... ..T ... ... ... ... ... ... ... ... ... ... ... ... ... ... ... ... ... ... ... ... ... ... ... ... ... ... ... ... ... ...  [ 990]
TN91.320   ..T ... ..T ... ..T ... ... ... ... ... ... ... ... ... ... ... ... ... ... ... ... ... ... ... ... ... ... ... ... ... ... ... ...  [ 990]
TN91.334   ..T ... ..T ... ... ... ... ... ... ... ... ... ... ... ... ... ... ... ... ... ... ... ... ... ... ... ... ... ... ... ... ... ...  [ 990]
TN91.349   ..T ... ..T ... ..T ... ... ... ... ... ... ... ... ... ... ... ... ... ... ... ... ... ... ... ... ... ... ... ... ... ... ... ...  [ 990]
TN91.451   ..T ... ..T ... ..T ... ... ... ... ... ... ... ... ... ... ... ... ... ... ... ... ... ... ... ... ... ... ... ... ... ... ... ...  [ 990]
TN91.521   ..T ... ..T ... ..T ... ... ... ... ... ... ... ... ... ... ... ... ... ... ... ... ... ... ... ... ... ... ... ... ... ... ... ...  [ 990]
TN92.1031  ..T ... ..T ... ... ... ... ... ... ... ... ... ... ... ... ... ... ... ... ... ... ... ... ... ... ... ... ... ... ... ... ... ...  [ 990]
```

```
TN92.1032 ..T ... ..T ... ... ... ... ... ... ... ... ... ... ... ... ... ... ... ... ... ... ... ... ... ... ... ... ... ... ... ... ... ...  [ 990]
TN92.1120 ..T ... ..T ... ... ... ... ... ... ... ... ... ... ... ... ... ... ... ... ... ... ... ... ... ... ... ... ... ... ... ... ... ...  [ 990]
TN93.32   ..T ... ..T ... ... ... ... ... ... ... ... ... ... ... ... ... ... ... ... ... ... ... ... ... ... ... ... ... ... ... ... ... ...  [ 990]
TN93.616  ..T ... ..T ... ... ... ... ... ... ... ... ... ... ... ... ... ... ... ... ... ... ... ... ... ... ... ... ... ... ... ... ... ...  [ 990]
TN94.413  ..T ... ..T ... ... ... ... ... ... ... ... ... ... ... ... ... ... ... ... ... ... ... ... ... ... ... ... ... ... ... ... ... ...  [ 990]
NL94.1    ..T ... ..T ... ... ... ... ... ... ... ... ... ... ..C ... ... ... ... ... ... ... ... ... ... ... ... ... ... ... ... ... ... ...  [ 990]
TN95.252  ..T ... ..T ... ..T ... ... ... ... ... ... ... ... ... ... ... ... ... ... ... ... ... ... ... ... ... ... ... ... ... ... ... ...  [ 990]
TN96.115  ..T ... ..T ... ... ... ... ... ... ... ... ... ... ... ... ... ... ... ... ... ... ... ... ... ... ... ... ... ... ... ... ... ...  [ 990]
TN96.35   ..T ... ..T ... ... ... ... ... ... ... ... ... ... ... ... ... ... ... ... ... ... ... ... ... ... ... ... ... ... ... ... ... ...  [ 990]
TN96.213  ..T ... ..T ... ... ... ... ... ... ... ... ... ... ... ... ... ... ... ... ... ... ... ... ... ... ... ... ... ... ... ... ... ...  [ 990]
TN96.44   ..T ... ..T ... ... ... ... ... ... ... ... ... ... ... ... ... ... ... ... ... ... ... ... ... ... ... ... ... ... ... ... ... ...  [ 990]
TN97.235  ... ... ..T ... ... ..G ... ... ... ... ... ... ..C ..C ... ... ... ... ... ... ... ... ... ... ... ... ... ... ... ... ... ... ...  [ 990]
TN98.512  ..T ... ..T ... ... ... ... ... ... ... ... ... ... ... ... ... ... ... ... ... ... ... ... ... ... ... ... ... ... ... ... ... ...  [ 990]
TN99.419  ..T ... ..T ... ... ... ... ... ... ... ... ... ... ... ... ... ... ... ... ... ... ... ... ... ... ..T ... ... ... ... ... ... ...  [ 990]
CAN98.73  ..T ... ..T ... ... ... ... ... ... ... ... ... ... ... ... ... ... ... ... ... ... ... ... ... ... ... ... ..T ... ... ... ... ...  [ 990]
CAN98.79  ..T ... ..T ... ... ... ... ... ... ... ... ... ... ... ... ... ... ... ... ... ... ... ... ... ... ... ... ..T ..T ... ... ... ...  [ 990]
CAN00.13  ..T ... ..T ... ... ... ... ... ... ... ... ... ... ... ... ... ... ... ... ... ... ... ... ... ... ... ... ... ... ... ... ... ...  [ 990]
TN01.28   ..T ... ..T ... ... ... ... ... ... ... ... ... ... ... ... ... ... ... ... ... ... ... ... ... ... ... ... ... ... ... ... ... ...  [ 990]

TN82.59   GAT CAT GTT TTT TGT GAC ACA GCG GCA GGG ATC AAT GTT GCT GAG CAA TCA AGA GAA TGC AAC ATC AAC ATA TCT ACT ACC AAC TAC CCA TGC AAA GTC  [1089]
TN83.126  ... ... ... ... ... ... ... ... ... ... ... ... ... ... ... ... ... ... ... ... ... ... ... ... ... ... ... ... ... ... ..T ... ...  [1089]
TN83.1211 ... ... ... ... ... ... ... ... ... ... ... ... ... ... ... ... ... ... ... ... ... ... ... ... ... ... ... ... ... ... ..T ... ...  [1089]
TN84.611  ... ... ... ... ... ... ... ... ... ... ... ... ... ... ... ... ... ... ... ... ... ... ... ... ... ... ... ... ... ... ..T ... ...  [1089]
TN85.123  ... ... ... ... ... ... ... ..A ... ... ... ... ... ... ... ... ... ... ... ... ... ... ... ... ... ..C ... ... ... ... ... ... ...  [1089]
TN85.417  ... ... ... ... ... ... ... ..A ... ... ... ... ... ... ... ... ... ... ... ... ... ... ... ... ... ... ... ... ... ... ... ... ...  [1089]
TN89.356  ... ... ... ... ... ... ... ..A ... ... ... ... ... ... ... ... ... ... ... ... ... ... ... ... ... ... ... ... ... ... ... ... ...  [1089]
TN89.1144 ... ... ... ... ... ... ... ..A ... ... ... ... ... ... ... ... ... ... ... ... ... ... ... ... ... ..C ... ... ... ... ... ... ...  [1089]
TN89.1259 ... ... ... ... ... ... ... ..A ... ... ... ... ... ... ... ... ... ... ... ... ... ... ... ... ... ..C ... ... ... ... ... ... ...  [1089]
TN89.515  ... ... ... ... ... ... ... ..A ... ... ... ... ... ... ... ... ... ... ... ... ... ... ... ... ... ..C ... ... ... ... ... ... ...  [1089]
TN90.140  ... ... ... ... ... ... ... ..A ... ... ... ... ... ... ... ... ... ... ... ... ... ... ... ... ... ..C ... ... ... ... ... ... ...  [1089]
TN90.422  ... ... ... ... ... ... ... ..A ... ... ... ... ... ... ... ... ... ... ... ... ... ... ... ... ... ..C ... ... ... ... ... ... ...  [1089]
TN90.1031 ... ... ... ... ... ... ... ..A ... ... ... ... ... ... ... ... ... ... ... ... ... ... ... ... ... ..C ... ... ... ... ... ... ...  [1089]
TN91.316  ... ... ... ... ... ..T ... ..A ... ... ... ... ... ... ... ... ... ... ... ... ... ... ... ... ... ..C ... ... ... ... ... ... ...  [1089]
TN91.320  ... ... ... ... ..C ... ... ..A ... ... ... ... ... ... ... ... ... ... ... ... ... ... ... ... ... ..C ... ... ... ... ... ... ...  [1089]
TN91.334  ... ... ... ... ... ... ... ..A ... ... ... ... ... ... ... ... ... ... ... ... ... ... ... ... ... ..C ... ... ... ... ... ... ...  [1089]
TN91.349  ... ... ... ... ..C ... ... ..A ... ... ... ... ... ... ... ... ... ... ... ... ... ... ... ... ... ..C ... ... ... ... ... ... ...  [1089]
TN91.451  ... ... ... ... ... ... ... ..A ... ... ... ... ... ... ... ... ... ... ... ... ... ... ... ... ... ..C ... ..G ... ... ... ... ...  [1089]
TN91.521  ... ... ... ... ... ... ... ..A ... ... ... ... ... ... ... ... ... ... ... ... ... ... ... ... ... ..C ... ... ... ... ... ... ...  [1089]
TN92.1031 ... ... ... ... ... ... ... ..A ... ... ... ... ... ... ... ... ... ... ... ... ... ... ... ... ... ..C ... ... ... ... ... ... ...  [1089]
TN92.1032 ... ... ... ... ... ... ... ..A ... ... ... ... ... ... ... ... ... ... ... ... ... ... ... ... ... ..C ... ... ... ... ... ... ...  [1089]
TN92.1120 ... ... ... ... ... ... ... ..T ... ... ... ... ... ... ... ... ... ... ... ... ... ... ... ... ... ..C ... ... ... ... ... ... ...  [1089]
TN93.32   ... ... ... ... ... ... ... ..A ... ... ... ... ... ... ... ... ... ... ... ... ... ... ... ... ... ..C ... ... ... ... ... ... ...  [1089]
TN93.616  ... ... ... ... ... ... ... ..A ... ... ... ... ... ... ... ... ... ... ... ... ... ... ... ... ... ..C ... ... ... ... ... ... ...  [1089]
TN94.413  ... ... ... ... ... ... ... ..A ... ... ... ... ... ... ... ... ... ... ... ... ... ... ... ... ... ..C ... ... ... ... ... ... ...  [1089]
NL94.1    ... ... ... ... ... ... ... ..A ... ... ... ... ... ... ... ... ... ... ... ... ... ... ... ... ... ..C ... ... ... ... ... ... ...  [1089]
TN95.252  ... ... ... ... ... ... ... ..A ... ... ... ... ... ..C ... ... ... ... ... ... ... ... ... ... ..C ..C ... ... ... ... ... ... ...  [1089]
TN96.115  ... ... ... ... ... ... ... ..A ... ... ... ... ... ... ... ... ... ... ... ... ... ..T ... ... ... ..C ... ... ... ... ... ... ...  [1089]
TN96.35   ... ... ... ... ... ... ... ..A ... ... ... ... ... ... ... ... ... ... ... ... ... ..T ... ... ... ..C ... ... ... ... ... ... ...  [1089]
TN96.213  ... ... ... ... ... ... ... ..A ... ... ... ... ... ... ... ... ... ..G ... ... ... ..T ... ... ... ..C ... ... ... ... ... ... ...  [1089]
TN96.44   ... ... ... ... ... ... ... ..A ... ... ... ... ... ... ... ... ... ... ... ... ... ..T ... ... ... ..C ... ... ... ... ... ... ...  [1089]
TN97.235  ... ... ... ... ... ... ... ... ... ... ... ... ... ... ... ... ... ... ... ... ... ... ... ... ..C ... ... ... ... ... ... ... ...  [1089]
TN98.512  ... ... ... ... ... ... ... ..A ... ... ... ... ... ... ... ... ... ... ... ... ... ... ... ... ... ..C ... ... ... ... ... ... ...  [1089]
TN99.419  ... ... ... ... ... ... ... ..A ... ... ... ... ... ... ... ... ... ... ... ... ... ... ... ... ... ..A ... ... ... ... ... ... ...  [1089]
```

```
CAN98.73   ... ... ... ... ... ... ... ..T ... ... ... ... ... ... ... ... ... ... ... ... ... ... ... ... ..C ..A ... ... ... ... ... ... ...  [1089]
CAN98.79   ... ... ... ... ... ... ... ..T ... ... ... ... ... ... ... ... ... ... ... ... ... ... ... ... ... ..A ... ... ... ... ... ... ...  [1089]
CAN00.13   ... ... ... ... ... ... ... ..A ... ... ... ... ... ... ..A ... ... ... ... ... ... ... ... ... ... ..A ... ... ... ... ... ... ...  [1089]
TN01.28    ... ... ... ... ... ... ... ..A ... ... ... ... ... ... ... ... ... ... ... ... ... ... ... ... ... ..C ... ... ... ... ... ... ...  [1089]

TN82.59    AGC ACA GGA AGA CAC CCT ATC AGC ATG GTT GCA CTA TCA CCT CTC GGT GCT TTG GTG GCT TGC TAC AAA GGG GTT AGC TGC TCG ATT GGC AGC AAT CGA  [1188]
TN83.126   ... ... ... ... ... ... ... ... ... ... ... ... ... ... ... ... ... ... ... ... ..T ... ... ... ... ... ... ... ... ... ..T ... ...  [1188]
TN83.1211  ... ... ... ... ... ... ... ... ... ... ... ... ... ... ... ... ... ... ... ... ..T ... ... ... ... ... ... ... ... ... ..T ... ...  [1188]
TN84.611   ... ... ... ... ... ... ... ... ... ... ... ... ... ... ... ... ... ... ... ... ..T ... ... ... ... ... ... ... ... ... ..T ... ...  [1188]
TN85.123   ... ... ... ... ... ... ... ... ... ... ... ... ... ... ... ... ... ... ..A ... ... ... ... ... ... ... ... ... ... ... ..T ... ..G  [1188]
TN85.417   ... ... ... ... ... ... ... ... ... ... ... ... ... ... ... ... ... ... ..A ... ... ... ... ... ... ... ... ... ... ... ... ... ..G  [1188]
TN89.356   ... ... ... ... ... ... ... ... ... ... ... ... ... ... ... ... ... ... ..A ... ... ... ... ... ... ... ... ... ... ... ..T ... ..G  [1188]
TN89.1144  ... ... ... ... ... ... ... ... ... ... ... ... ... ... ... ... ..C ... ..A ... ... ... ... ... ... ... ... ... ... ..T ..T ... ..G  [1188]
TN89.1259  ... ... ... ... ... ... ... ... ... ... ... ... ... ... ... ... ..C ... ..A ... ... ... ... ... ... ... ... ... ... ..T ..T ... ..G  [1188]
TN89.515   ... ... ... ... ... ... ... ... ... ... ... ... ... ... ... ... ..C ... ..A ... ... ... ... ... ... ... ... ... ... ..T ..T ... ..G  [1188]
TN90.140   ... ... ... ... ... ... ... ... ... ... ... ... ... ... ... ... ..C ... ..A ... ... ... ... ... ... ... ... ... ... ..T ..T ... ..G  [1188]
TN90.422   ... ... ... ... ... ... ... ... ... ... ... ... ... ... ... ... ..C ... ..A ... ... ... ... ... ... ... ... ... ... ..T ..T ... ..G  [1188]
TN90.1031  ... ... ... ... ... ... ... ... ... ... ... ... ... ... ... ... ... ... ..A ... ... ... ... ... ... ... ... ... ... ... ..T ... ..G  [1188]
TN91.316   ... ... ... ... ... ... ... ... ... ... ... ... ... ... ... ... ... ... ..A ... ... ... ... ... ... ... ... ... ... ... ..T ... ..G  [1188]
TN91.320   ... ... ... ... ... ... ... ... ... ... ... ... ... ... ... ... ... ... ..A ... ... ... ... ... ..C ... ... ... ... ... ..T ... ..G  [1188]
TN91.334   ... ... ... ... ... ... ... ... ... ... ... ... ... ... ... ... ... ... ..A ... ... ... ... ... ... ... ... ... ... ..T ..T ... ..G  [1188]
TN91.349   ... ... ... ... ... ... ... ... ... ... ... ... ... ... ... ... ... ... ..A ... ... ... ... ... ..C ... ... ... ... ... ..T ... ..G  [1188]
TN91.451   ... ... ... ... ... ... ... ... ... ... ... ... ... ... ... ... ... ... ..A ... ... ... ... ... ..C ... ... ... ... ... ..T ... ..G  [1188]
TN91.521   ... ... ... ... ... ... ... ... ... ... ... ... ... ... ... ... ... ... ..A ... ... ... ... ... ..C ... ... ... ... ... ..T ... ..G  [1188]
TN92.1031  ... ... ... ... ... ... ... ... ... ... ... ... ... ... ... ... ... ... ..A ... ... ... ... ... ... ... ... ... ... ... ..T ... ..G  [1188]
TN92.1032  ... ... ... ... ... ... ... ... ... ... ... ... ... ... ... ... ... ... ..A ... ... ... ... ... ... ... ... ... ... ... ..T ... ..G  [1188]
TN92.1120  ... ... ... ... ... ... ... ... ... ... ... ... ... ... ... ... ... ... ..A ... ... ... ... ... ... ... ... ... ... ... ..T ... ..G  [1188]
TN93.32    ... ... ... ... ... ... ... ... ... ... ... ... ... ... ... ... ... ... ..A ... ... ... ... ... ... ... ... ... ... ... ..T ... ..G  [1188]
TN93.616   ... ... ... ... ... ... ... ... ... ... ... ... ... ... ... ... ... ... ..A ... ... ... ... ... ... ... ... ... ... ... ..T ... ..G  [1188]
TN94.413   ... ... ... ... ... ... ... ... ... ... ... ... ... ... ... ... ... ... ..A ... ... ... ... ... ... ... ... ... ... ... ..T ... ..G  [1188]
NL94.1     ... ... ... ... ... ... ... ... ... ... ... ... ... ... ... ... ... ... ..A ... ... ... ..G ... ... ... ... ... ... ... ..T ... ..G  [1188]
TN95.252   ... ... ... ... ... ... ... ... ... ... ... ... ... ... ... ... ... ... ..A ... ... ... ... ... ... ... ... ... ... ... ..T ... ..G  [1188]
TN96.115   ... ... ... ... ... ... ..T ... ... ... ... ... ... ... ... ... ... ... ..A ... ... ... ... ... ... ... ... ... ... ... ..T ... ..G  [1188]
TN96.35    ... ... ... ... ... ... ..T ... ... ... ... ... ... ... ... ... ... ... ..A ... ... ... ... ... ... ... ... ... ... ... ..T ... ..G  [1188]
TN96.213   ... ... ... ... ... ... ..T ... ... ... ... ... ... ... ... ... ... ... ..A ... ... ... ... ... ... ... ... ... ... ... ..T ... ..G  [1188]
TN96.44    ... ... ... ... ... ... ..T ... ... ... ... ... ... ... ... ... ... ... ..A ... ... ... ... ... ... ... ... ... ... ... ..T ... ..G  [1188]
TN97.235   ... ... ... ... ... ... ... ... ... ... ... ... ... ... ... ... ... ... ... ... ... ... ... ... ... ... ... ... ... ... ... ... ...  [1188]
TN98.512   ... ... ... ... ... ..C ... ... ... ... ... ... ... ... ... ... ... ... ..A ... ... ... ... ... ... ... ... ... ... ... ..T ... ..G  [1188]
TN99.419   ... ... ... ... ... ... ... ... ... ... ... ... ... ... ... ... ... ... ..A ... ... ... ... ... ... ... ..T ... ... ... ..T ... ..G  [1188]
CAN98.73   ... ... ... ... ... ... ... ... ... ... ... ... ... ... ... ... ... ... ..A ... ... ... ... ... ... ... ..T ..A ... ... ..T ... ..G  [1188]
CAN98.79   ... ... ... ... ... ... ... ... ... ... ... ... ... ... ... ... ... ... ..A ... ... ... ... ... ... ... ..T ..A ... ... ..T ... ..G  [1188]
CAN00.13   ... ... ... ... ... ... ... ... ... ... ... ... ... ... ... ... ... ... ..A ... ... ... ... ... ... ... ..T ... ... ... ..T ... ..G  [1188]
TN01.28    ... ... ... ... ... ..C ... ... ... ... ... ... ... ... ... ... ... ... ..A ... ... ... ... ... ..C ... ... ... ... ... ..T ... ..G  [1188]

TN82.59    GTT GGA ATA ATC AAA CAA CTA CCC AAA GGC TGC TCA TAC ATA ACT AAC CAG GAC GCA GAC ACT GTA ACA ATT GAC AAT ACC GTG TAT CAA CTA AGC AAA  [1287]
TN83.126   ... ... ... ... ... ... ... ..T ... ... ... ... ... ... ... ... ... ... ... ... ... ... ... ... ... ... ... ... ... ... ... ... ...  [1287]
TN83.1211  ... ... ... ... ... ... ... ..T ... ... ... ... ... ... ... ... ... ... ... ... ... ... ... ... ... ... ... ... ... ... ... ... ...  [1287]
TN84.611   ... ... ... ... ... ... ... ... ... ... ... ... ... ... ... ... ... ... ... ... ... ... ... ... ... ... ... ... ... ... ... ... ...  [1287]
TN85.123   ... ... ... ... ... ... ... ..T ... ..A ... ... ... ... ... ... ... ... ... ... ... ... ... ... ... ..C ... ... ... ... ... ... ...  [1287]
TN85.417   ... ... ... ... ... ... ... ..T ... ... ... ... ... ... ... ... ... ... ... ... ... ... ... ... ... ..C ... ... ... ... ... ... ...  [1287]
TN89.356   ... ... ... ... ... ... ... ..T ... ... ... ... ... ... ... ... ... ... ... ... ... ... ... ... ... ..C ..T ... ... ... ... ... ...  [1287]
TN89.1144  ... ... ... ... ... ... ... ..T ... ... ... ... ... ... ... ... ... ... ... ..T ... ... ... ... ... ..C ..T ... ... ... ... ... ...  [1287]
TN89.1259  ... ... ... ... ... ... ... ..T ... ... ... ... ... ... ... ... ... ... ... ..T ... ... ... ... ... ..C ..T ... ... ... ... ... ...  [1287]
```

```
TN89.515   ... ... ... ... ... ... ... ..T ... ... ... ... ... ... ... ... ... ... ... ..T ... ... ... ... ... ..C ..T ... ... ... ... ... ...  [1287]
TN90.140   ... ... ... ... ... ... ... ..T ... ... ... ... ... ... ... ... ... ... ... ..T ... ... ... ... ... ..C ..T ... ... ... ... ... ...  [1287]
TN90.422   ... ... ... ... ... ... ... ..T ... ... ... ... ... ... ... ... ... ... ... ..T ... ... ... ... ... ..C ..T ... ... ... ... ... ...  [1287]
TN90.1031  ... ... ... ... ... ... ... ..T ... ... ... ... ... ... ... ... ... ... ... ... ... ... ... ... ... ..C ..T ... ... ... ... ... ...  [1287]
TN91.316   ... ... ... ... ... ... ... ..T ... ... ... ... ... ... ... ... ... ... ... ... ... ... ... ... ... ..C ..T ... ... ... ... ... ...  [1287]
TN91.320   ... ... ... ... ... ... ... ..T ... ... ... ... ... ... ... ... ... ... ... ... ... ... ... ... ... ..C ..T ... ..C ... ... ... ...  [1287]
TN91.334   ... ... ... ... ... ... ... ..T ... ... ... ... ... ... ... ... ... ... ... ... ... ... ... ... ... ..C ..T ... ... ... ... ... ...  [1287]
TN91.349   ... ... ... ... ... ... ... ..T ... ... ... ... ... ... ... ... ... ... ... ... ... ... ... ... ... ..C ..T ... ..C ... ... ... ...  [1287]
TN91.451   ... ... ... ... ... ... ... ..T ... ... ... ... ... ... ... ... ... ... ... ... ... ... ... ... ... ..C ..T ... ..C ... ... ..T ...  [1287]
TN91.521   ... ... ... ... ... ... ... ..T ... ... ... ... ... ... ... ... ... ... ... ... ... ... ... ... ... ..C ..T ... ..C ... ... ... ...  [1287]
TN92.1031  ... ... ... ... ... ... ... ..T ... ... ... ... ... ... ... ... ... ... ... ... ... ... ... ... ... ..C ..T ... ... ... ... ... ...  [1287]
TN92.1032  ... ... ... ... ... ... ... ..T ... ... ... ... ... ... ... ... ... ... ... ... ... ... ... ... ... ..C ..T ... ... ... ... ... ...  [1287]
TN92.1120  ... ... ... ... ... ... ... ..T ... ... ... ... ... ... ... ... ... ... ... ..T ... ... ... ... ... ..C ..T ... ... ... ... ... ...  [1287]
TN93.32    ... ... ... ... ... ... ... ..T ... ... ... ... ... ... ... ... ... ... ... ... ... ... ... ... ... ..C ..T ... ... ... ... ... ...  [1287]
TN93.616   ... ... ... ... ... ... ... ..T ... ... ... ... ... ... ... ... ... ... ... ... ... ... ... ... ... ..C ..T ... ... ... ... ... ...  [1287]
TN94.413   ... ... ... ... ... ... ... ..T ... ... ... ... ... ... ... ... ... ... ... ... ... ... ... ... ... ..C ..T ... ... ... ... ... ...  [1287]
NL94.1     ... ... ... ... ... ... ... ..T ... ... ... ... ... ... ... ... ... ... ... ... ... ... ... ... ... ..C ..T ... ... ... ... ... ...  [1287]
TN95.252   ... ... ... ... ... ... ... ..T ... ... ... ... ... ... ... ... ... ... ... ... ... ... ... ... ... ..C ..T ..A ... ... ... ... ...  [1287]
TN96.115   ... ... ... ... ... ... ... ..T ... ... ... ... ... ... ... ... ... ... ... ... ... ... ... ... ... ..C ..T ..A ... ... ... ... ...  [1287]
TN96.35    ... ... ... ... ... ... ... ..T ... ... ... ... ... ... ... ... ... ... ... ... ... ... ... ... ... ..C ..T ..A ... ... ... ... ...  [1287]
TN96.213   ... ... ... ... ... ... ... ..T ... ... ... ... ... ... ... ... ... ... ... ... ... ... ... ... ... ..C ..T ..A ... ... ... ... ...  [1287]
TN96.44    ... ... ... ... ... ... ... ..T ... ... ... ... ... ... ... ... ... ... ... ... ... ... ... ... ... ..C ..T ..A ... ... ... ... ...  [1287]
TN97.235   ... ... ... ... ... ... ... ..T ... ... ... ... ... ... ... ... ... ... ... ... ... ... ... ... ... ... ... ... ... ... ... ... ...  [1287]
TN98.512   ... ... ... ... ... ... ... ..T ... ... ... ... ... ... ... ... ... ... ... ... ... ... ... ... ... ..C ..T ..A ... ... ... ... ...  [1287]
TN99.419   ... ... ... ... ... ... ... ..T ... ... ... ... ... ... ... ... ... ... ... ... ... ... ... ... ... ..C ..T ... ... ... ... ... ...  [1287]
CAN98.73   ... ... ... ... ... ... ... ..T ... ... ... ... ... ... ... ... ... ... ... ... ... ... ... ... ... ..C ..T ... ... ... ... ... ...  [1287]
CAN98.79   ... ... ... ... ... ... ... ..T ... ... ... ... ... ... ... ... ... ... ... ... ... ... ... ... ... ..C ..T ... ... ... ... ... ...  [1287]
CAN00.13   ... ... ... ... ... ... ... ..T ... ... ... ... ... ... ... ... ... ... ... ... ... ... ... ... ... ..C ..T ... ... ... ... ... ...  [1287]
TN01.28    ... ... ... ... ... ... ... ..T ... ... ... ... ... ... ... ... ... ... ... ... ... ..G ... ... ... ..C ..T ... ... ... ... ... ...  [1287]

TN82.59    GTT GAG GGT GAA CAG CAT GTA ATA AAA GGA AGA CCA GTT TCA AGC AGT TTT GAT CCA ATT AGG TTT CCT GAG GAT CAG TTC AAT GTT GCG CTT GAT CAA  [1386]
TN83.126   ... ... ... ... ... ... ... ... ... ... ... ... ... ... ... ... ... ... ... ..C ... ... ... ... ... ... ... ... ... ... ... ... ...  [1386]
TN83.1211  ... ... ... ... ... ... ... ... ... ... ... ... ... ... ... ... ... ... ... ..C ... ... ... ... ... ... ... ... ... ... ... ... ...  [1386]
TN84.611   ... ... ... ... ... ... ... ... ... ... ... ... ... ... ... ... ... ... ... ..C ... ... ... ... ... ... ... ... ... ... ... ... ...  [1386]
TN85.123   ... ... ... ... ... ... ... ... ... ..G ... ... ... ... ... ... ... ... ... ..C ... ... ... ... ... ... ... ... ... ... ... ... ...  [1386]
TN85.417   ... ... ... ... ... ... ... ... ... ..G ... ... ... ... ... ... ... ... ... ..C ... ... ... ... ... ... ... ... ... ... ... ... ...  [1386]
TN89.356   ... ... ... ... ... ... ... ... ... ..G ... ... ... ... ... ... ... ... ... ..C ... ... ... ... ... ... ... ... ... ... ... ... ...  [1386]
TN89.1144  ... ... ... ... ... ... ... ... ... ..G ... ... ... ... ... ... ... ... ... ..C ... ... ... ... ... ... ... ... ... ... ... ... ...  [1386]
TN89.1259  ... ... ... ... ... ... ... ... ... ..G ... ... ... ... ... ... ... ... ... ..C ... ... ... ... ... ... ... ... ... ... ... ... ...  [1386]
TN89.515   ... ... ... ... ... ... ... ... ... ..G ... ... ... ... ... ... ... ... ... ..C ... ... ... ... ... ... ... ... ... ... ... ... ...  [1386]
TN90.140   ... ... ... ... ... ... ... ... ... ..G ... ... ... ... ... ... ... ... ... ..C ... ... ... ... ... ... ... ... ... ... ... ... ...  [1386]
TN90.422   ... ... ... ... ... ... ... ... ... ..G ... ... ... ... ... ... ... ... ... ..C ... ... ... ... ... ... ... ... ... ... ... ... ...  [1386]
TN90.1031  ... ... ... ... ... ... ... ... ... ..G ... ... ... ... ... ... ..C ... ... ..C ... ... ... ... ... ... ... ... ... ... ... ... ...  [1386]
TN91.316   ... ... ... ... ... ... ... ... ... ..G ... ... ... ... ... ... ... ... ... ..C ... ... ... ... ... ... ... ... ... ... ... ... ...  [1386]
TN91.320   ... ... ... ... ... ... ... ... ... ..G ... ... ... ... ... ... ... ... ... ..C ... ... ... ... ... ... ... ... ... ... ... ... ...  [1386]
TN91.334   ... ... ... ... ... ... ... ... ... ..G ... ... ... ... ... ... ... ... ... ..C ... ... ... ... ... ... ... ... ... ... ... ... ...  [1386]
TN91.349   ... ... ... ... ... ... ... ... ... ..G ... ... ... ... ... ... ... ... ... ..C ... ... ... ... ... ... ... ... ... ... ... ... ...  [1386]
TN91.451   ... ... ... ... ... ... ... ... ... ..G ... ... ... ... ... ... ... ... ... ..C ... ... ... ... ... ... ... ... ... ... ... ... ...  [1386]
TN91.521   ... ... ... ... ... ... ... ... ... ..G ... ... ... ... ... ... ... ... ... ..C ... ... ... ... ... ... ... ... ... ... ... ... ...  [1386]
TN92.1031  ... ... ... ... ... ... ... ... ... ..G ... ... ... ... ... ... ... ... ... ..C ... ... ... ... ... ... ... ... ... ... ... ... ...  [1386]
TN92.1032  ... ... ... ... ... ... ... ... ... ..G ... ... ... ... ... ... ... ... ... ..C ... ... ... ... ... ... ... ... ... ... ... ... ...  [1386]
TN92.1120  ... ... ... ... ... ... ... ... ... ..G ... ... ... ... ... ... ... ... ... ..C ... ... ... ... ... ... ... ... ... ... ... ... ...  [1386]
TN93.32    ... ... ... ... ... ... ... ... ... ..G ... ... ... ... ... ... ... ... ... ..C ... ... ... ... ... ... ... ... ... ... ... ... ...  [1386]
```

```
TN93.616    ... ... ... ... ... ... ... ... ... ..G ... ... ... ... ... ... ... ... ... ..C ... ... ... ... ... ... ... ... ... ... ... ... ...  [1386]
TN94.413    ... ... ... ... ... ... ... ... ... ..G ... ... ... ... ... ... ... ... ... ..C ... ... ... ... ... ... ... ... ... ... ... ... ...  [1386]
NL94.1      ... ... ... ... ... ... ... ... ... ..G ... ... ... ... ... ... ... ... ... ..C ... ... ... ... ... ... ... ... ... ... ... ... ...  [1386]
TN95.252    ... ... ... ... ... ... ... ... ... ..G ... ... ... ... ... ... ... ... ... ..C ... ... ... ... ... ... ... ... ... ... ... ... ...  [1386]
TN96.115    ... ... ... ... ... ... ... ... ... ..G ... ... ... ... ... ... ... ... ... ..C ... ... ... ... ... ... ... ... ... ... ... ... ...  [1386]
TN96.35     ... ... ... ... ... ... ... ... ... ..G ... ... ... ... ... ... ... ... ... ..C ... ... ... ... ... ... ... ... ... ... ... ... ...  [1386]
TN96.213    ... ... ... ... ... ... ... ... ... ..G ... ... ... ... ... ... ... ... ... ..C ... ... ... ... ... ... ... ... ... ... ... ... ...  [1386]
TN96.44     ... ... ... ... ... ... ... ... ... ..G ... ... ... ... ... ... ... ... ... ..C ... ... ... ... ... ... ... ... ... ... ... ... ...  [1386]
TN97.235    ... ... ... ... ... ... ... ... ... ... ... ... ... ... ... ... ... ... ... ..C ... ... ... ... ... ... ... ... ... ... ... ... ...  [1386]
TN98.512    ... ... ... ... ... ... ... ... ... ..G ... ... ... ... ... ... ... ... ... ..C ..A ... ... ... ... ... ... ... ... ... ... ..C ...  [1386]
TN99.419    ... ... ... ... ... ... ... ... ... ..G ... ... ... ... ... ... ..C ... ... ..C .A. ... ... ... ... ... ... ... ... ... ... ... ...  [1386]
CAN98.73    ... ... ... ... ... ... ... ... ... ..G ... ... ... ... ... ... ..C ... ... ..C .A. ... ... ... ... ... ... ... ... ... ... ... ...  [1386]
CAN98.79    ... ... ... ... ... ... ... ... ... ..G ... ... ... ... ... ... ..C ... ... ..C .A. ... ... ... ... ... ... ... ... ... ... ... ...  [1386]
CAN00.13    ... ... ... ... ... ... ... ... ... ..G ... ... ... ... ... ... ..C ... ... ..C .A. ... ... ... ... ... ... ... ... ..A ... ... ...  [1386]
TN01.28     ... ... ... ... ... ... ... ... ... ..G ... ... ... ... ... ... ... ... ... ..C ..A ... ... ... ... ... ... ... ... ... ... ..C ...  [1386]

TN82.59     GTC TTT GAA AGC ATT GAA AAC AGT CAA GCA CTA GTG GAC CAG TCA AAC AAA ATT CTG AAC AGT GCA GAA AAA GGA AAC ACT GGT TTC ATT ATT GTG ATA  [1485]
TN83.126    ... ... ... ... ... ... ... ... ... ... ... ... ... ... ... ... ... ... ... ... ... ... ... ... ... ... ... ... ... ... ... ... ...  [1485]
TN83.1211   ... ... ... ... ... ... ... ... ... ... ... ... ... ... ... ... ... ... ... ... ... ... ... ... ... ... ... ... ... ... ... ... ...  [1485]
TN84.611    ... ... ... ... ... ... ... ... ... ... ... ... ... ... ... ... ... ... ... ... ... ... ... ... ... ... ... ... ... ... ... ... ...  [1485]
TN85.123    ... ... ... ... ... ... ... ... ... ... ... ... ... ... ... ... ... ... ... ... ... ... ... ... ... ... ... ... ... ... ... ..A ...  [1485]
TN85.417    ... ... ... ... ... ... ... ... ... ... ... ... ... ... ... ... ... ... ... ... ... ... ... ... ... ... ... ... ... ... ... ..A ...  [1485]
TN89.356    ... ... ... ... ... ... ... ... ... ... ... ... ... ... ... ... ... ... ... ... ... ... ... ... ... ... ... ... ... ... ... ..A ...  [1485]
TN89.1144   ... ... ... ... ... ... ... ... ... ... ... ... ... ... ... ... ... ... ... ... ... ... ... ... ... ... ... ... ... ... ... ..A ...  [1485]
TN89.1259   ... ... ... ... ... ... ... ... ... ... ... ... ... ... ... ... ... ... ... ... ... ... ... ... ... ... ... ... ... ... ... ..A ...  [1485]
TN89.515    ... ... ... ... ... ... ... ... ... ... ... ... ... ... ... ... ... ... ... ... ... ... ... ... ... ... ... ... ... ... ... ..A ...  [1485]
TN90.140    ... ... ... ... ... ... ... ... ... ... ... ... ... ... ... ... ... ... ... ... ... ... ... ... ... ... ... ... ... ... ... ..A ...  [1485]
TN90.422    ... ... ... ... ... ... ... ... ... ... ... ... ... ... ... ... ... ... ... ... ... ... ... ... ... ... ... ... ... ... ... ..A ...  [1485]
TN90.1031   ... ... ... ... ... ... ... ... ... ... ... ... ... ... ... ... ... ... ... ... ... ... ... ... ... ... ... ..C ... ... ... ..A ...  [1485]
TN91.316    ... ... ... ... ... ... ... ... ... ... ... ... ... ... ... ... ... ... ... ... ... ... ... ... ... ... ... ... ... ... ... ..A ...  [1485]
TN91.320    ... ... ... ... ... ... ... ... ... ... T.. ... ... ... ... ... ... ... ... ... ... ... ... ... ... ... ... ... ... ... ... ..A ...  [1485]
TN91.334    ... ... ... ... ... ... ... ... ... ... ... ... ... ... ... ... ... ... ... ... ... ... ... ... ... ... ... ... ... ... ... ..A ...  [1485]
TN91.349    ... ... ... ... ... ... ... ... ... ... T.. ... ... ... ... ... ... ... ... ... ... ... ... ... ... ... ... ... ... ... ... ..A ...  [1485]
TN91.451    ... ... ... ... ... ... ... ... ... ... T.. ... ... ... ... ... ... ... ... ... ... ... ... ... ... ... ... ... ... ... ... ..A ...  [1485]
TN91.521    ... ... ... ... ... ... ... ... ... ... T.. ... ... ... ... ... ... ... ... ... ... ... ... ... ... ... ... ... ... ... ... ..A ...  [1485]
TN92.1031   ... ... ... ... ... ... ... ... ... ... ... ... ... ... ... ... ... ... ... ... ... ... ... ... ... ... ... ... ... ... ... ..A ...  [1485]
TN92.1032   ... ... ... ... ... ... ... ... ... ... ... ... ... ... ... ... ... ... ... ... ... ... ... ... ... ... ... ... ... ... ... ..A ...  [1485]
TN92.1120   ... ... ... ... ... ... ... ... ... ... ... ... ... ... ... ... ... ... ... ... ... ... ... ... ... ... ... ... ... ... ... ..A ...  [1485]
TN93.32     ... ... ... ... ... ... ... ... ... ... ... ... ... ... ... ... ... ... ... ... ... ... ... ... ... ... ... ... ... ... ... ..A ...  [1485]
TN93.616    ... ... ... ... ... ... ... ... ... ... ... ... ... ... ... ... ... ... ... ... ... ... ... ... ... ... ... ... ... ... ... ..A ...  [1485]
TN94.413    ... ... ... ... ... ... ... ... ... ... ... ... ... ... ... ... ... ... ... ... ... ... ... ... ... ... ... ... ... ... ... ..A ...  [1485]
NL94.1      ... ... ... ... ... ... ... ... ... ... ... ... ... ... ... ... ... ... ... ... ... ... ... ... ... ... ... ... ... ... ... ..A ...  [1485]
TN95.252    ... ... ... ... ... ... ... ... ... ... ... ... ... ... ... ... ... ... ... ... ... ... ... ... ... ..T ... ... ... ... ... ..A ...  [1485]
TN96.115    ... ... ... ... ... ... ... ... ... ... ... ... ... ... ... ... ... ... ... ... ... ... ... ... ... ... ... ... ... ... ... ..A ...  [1485]
TN96.35     ... ... ... ... ... ... ... ... ... ... ... ... ... ... ... ... ... ... ... ... ... ... ... ... ... ... ... ... ... ... ... ..A ...  [1485]
TN96.213    ... ... ... ... ... ... ... ... ..G ... ... ... ... ... ... ... ... ... ... ... ... ... ... ... ... ... ... ... ... ... ... ..A ...  [1485]
TN96.44     ... ... ... ... ... ... ... ... ... ... ... ... ... ... ... ... ... ... ... ... ... ... ... ... ... ... ... ... ... ... ... ..A ...  [1485]
TN97.235    ... ... ... ... ... ... ... ... ... ... ... ... ... ... ... ... ... ... ... .G. ... ... ... ... ... ... ... ..C ... ... ... ... ...  [1485]
TN98.512    ... ... ... ... ... ... ... ... ... ... ... ... ... ... ... ... ... ... ... ... ... ... ... ... ... ... ... ... ... ... ... ..A ...  [1485]
TN99.419    ... ... ... ... ... ... ... ... ... ... ... ... ... ... ... ... ... ... ... ... ... ... ... ... ... ... ... ..C ... ... ... ..A ...  [1485]
CAN98.73    ... ... ... ... ... ... ... ... ... ... ... ... ... ... ... ... ... ... ... ... ... ... ... ... ... ... ..C ..C ... ... ... ..A ...  [1485]
CAN98.79    ... ... ... ... ... ... ... ... ... ... ... ... ... ... ... ... ... ... ... ... ... ... ... ... ... ... ... ..C ... ... ... ..A ...  [1485]
CAN00.13    ... ... ... ... ... ... ... ... ... ... ... ... ... ... ... ... ... ... ... ... ... ... ... ... ... ... ... ..C ... ... ... ..A ...  [1485]
```

```
TN01.28    ..T ... ... ... ... ... ... ... ... ... ... ... ... ... ... ... ... ... ... ... ... ... ... ... ... ... ... ... ... ... ... ..A ...  [1485]

TN82.59    ATT TTA ATT GCT GTT CTT GGT TTA ACC ATG ATT TCA GTG AGC ATC ATC ATC ATA ATC AAA AAA ACA AGG AAG CCC ACA GGA GCA CCT CCA GAG CTG AAT  [1584]
TN83.126   ... ..G ... ... ... ... ... ... ... ... ..C ... ... ... ... ... ... ... ... ... ..G ... ... ... ... ... ... ... ... ... ... ... ...  [1584]
TN83.1211  ... ..G ... ... ... ... ... ... ... ... ..C ... ... ... ... ... ... ... ... ... ..G ... ... ... ... ... ... ... ... ... ... ... ...  [1584]
TN84.611   ... ..G ... ... ... ... ... ... ... ... ..C ... ... ... ... ... ... ... ... ... ..G ... ... ... ... ... ... ... ... ... ... ... ...  [1584]
TN85.123   ... ..G ... ... ... ... ..G ... ... ... ... ... ... ... ... ... ... ... ... ... ... ... ... ... ... ... ..G ... ... ... ... ... ...  [1584]
TN85.417   ... ..G ... ... ... ... ..G ... ... ... ... ... ... ... ... ... ... ... ... ... ... ... ... ... ... ... ... ... ... ... ... ... ...  [1584]
TN89.356   ... ..G ... ... ... ... ..G ... ... ... ... ... ... ... ... ... ... ... ... ... ... ... ... ... ... ... ..G ... ... ... ... T.. ...  [1584]
TN89.1144  ... ..G ... ... ... ... ..G ... ... ... ... ... ... ... ... ... ... ... ... ... ... ... ... ... ... ... ..G ... ... ... ... ... ...  [1584]
TN89.1259  ... ..G ... ... ... ... ..G ... ... ... ... ... ... ... ... ... ... ... ... ... ... ... ... ... ... ... ..G ... ... ... ... ... ...  [1584]
TN89.515   ... ..G ... ... ... ... ..G ... ... ... ... ... ... ... ... ... ... ... ... ... ... ... ... ... ... ... ..G ... ... ... ... ... ...  [1584]
TN90.140   ... ..G ... ... ... ... ..G ... ... ... ... ... ... ... ... ... ... ... ... ... ... ... ... ... ... ... ..G ... ... ... ... ... ...  [1584]
TN90.422   ... ..G ... ... ... ... ..G ... ... ... ... ... ... ... ... ... ... ... ... ... ... ... ... ... ... ... ..G ... ... ... ... ... ...  [1584]
TN90.1031  ... ..G ... ... ... ... ..G ... ... ... ... ... ... ... ... ... ... ... ... ... ... ... ... ... ... ... ..G ... ... ... ... ... ...  [1584]
TN91.316   ... ..G ... ... ... ... ..G ... ... ... ... ... ... ... ... ... ... ... ... ... ... ... ... ... ... ... ..G ... ... ... ... ... ...  [1584]
TN91.320   ... ..G ... ..C ... ... ..G ... ... ... ... ... ... ... ... ... ... ... ... ... ... ... ... ... ... ... ... ... ... ... ... ... ...  [1584]
TN91.334   ... ..G ... ... ... ... ..G ... ... ... ... ... ... ... ... ... ... ... ... ... ... ... ... ... A.. ... ..G ... ... ... ... ... ...  [1584]
TN91.349   ... ..G ... ..C ... ... ..G ... ... ... ... ... ... ... ... ... ... ... ... ... ... ... ... ... ... ... ... ... ... ... ... ... ...  [1584]
TN91.451   ... ..G ... ..C ... ... ..G ... ... ... ... ... ... ... ... ... ... ... ... ... ... ... ... ... ... ... ... ... ... ... ... ... ...  [1584]
TN91.521   ... ..G ... ..C ... ... ..G ... ... ... ... ... ... ... ... ... ... ... ... ... ... ... ... ... ... ... ... ... ... ... ... ... ...  [1584]
TN92.1031  ... ..G ... ... ... ... ..G ... ... ... ... ... ... ... ... ... ... ... ... ... ... ... ... ... ... ... ..G ... ... ... ... ... ...  [1584]
TN92.1032  ... ..G ... ... ... ... ..G ... ... ... ... ... ... ... ... ... ... ... ... ... ... ... ... ... ... ... ..G ... ... ... ... ... ...  [1584]
TN92.1120  ... ..G ... ... ... ... ..G ... ... ... ... ... ... ... ... ... ... ... ... ... ... ... ... ... ... ... ..G ... ... ... ... ... ...  [1584]
TN93.32    ... ..G ... ... ... ... ..G ... ... ... ... ... ... ... ... ... ... ... ... ... ... ... ... ... ... ... ..G ... ... ... ... ... ...  [1584]
TN93.616   ... ..G ... ... ... ... ..G ... ... ... ... ... ... ... ... ... ... ... ... ... ... ... ... ... ... ... ..G ... ... ... ... ... ...  [1584]
TN94.413   ... ..G ... ... ... ... ..G ... ... ... ... ... ... ... ... ... ... ... ... ... ... ... ... ... ... ... ..G ... ... ... ... ... ...  [1584]
NL94.1     ... ..G ... ... ... ... ..G ... ... ... ... ... ... ... ... ... ... ... ... ... ... ... ... ... ... ... ..G ... ... ... ... ... ...  [1584]
TN95.252   ... ..G ... ... ... ... ..G ... ... ... ... ... ... ... ... ... ... ... ... ... ... ... ... ... ... ... ..G ... ... ... ... ... ...  [1584]
TN96.115   ... ..G ... ... ... ... ..G ... G.. ... ... ... ... ... ... ... ... ... ... ... ... ... ... ... ... ... ..G ... ... ... ... ... ...  [1584]
TN96.35    ... ..G ... ... ... ... ..G ... G.. ... ... ... ... ... ... ... ... ... ... ... ... ... ... ... ... ... ..G ... ... ... ... ... ...  [1584]
TN96.213   ... ..G ... ... ... ... ..G ... G.. ... ... ... ... ... ... ... ... ... ... ... ... ... ... ... ... ... ..G ... ... ... ... ... ...  [1584]
TN96.44    ... ..G ... ... ... ... ..G ... G.. ... ... ... ... ... ... ... ... ... ... ... ... ... ... ... ... ... ..G ... ... ... ... ... ...  [1584]
TN97.235   ... ..G G.. ... ... ... ... ... ... ... ... ... ... ... ... ... ... ... ... ... ..G ... .A. ... ... ... ... ... ... ... ... ... ...  [1584]
TN98.512   ... ..G ... ... ... ... ..G ... ... ... ... ... ... ... ... ... ... ... ... ... ... ... ... ... ... ... ..G ... ... ... ... ... ...  [1584]
TN99.419   ... ..G ... ... ... ... ..G ... ... ... ... ... ... ... ... ... ... ... ... N.. ... ... ... ..A ... ... ..G ... ... ... ... ... ...  [1584]
CAN98.73   ... ..G ... ... ... ... ..G ... ... ... ... ... ... ... ... ... ... ... ... ... ... ... ... ..A ... ... ..G ... ... ... ... ... ...  [1584]
CAN98.79   ... ..G ... ... ... ... ..G ... ... ... ... ... ... ... ... ... ... ... ... ... ... ... ... ..A ... ... ..G ... ... ... ... ... ...  [1584]
CAN00.13   ... ..G ... ... ... ... ..G ... ... ... ... ... ... ... ... ... ... ... ... ... ... ... ... ..A ... ... ..G ... ... ... ... ... ...  [1584]
TN01.28    ... ..G ... ... ... ... ..G ... ... ... ... ... ... ... ... ... ... ... ... ... ... ... ... ... ... ... ..G ... ... ... ... ... ...  [1584]

TN82.59    GGT GTT ACC AAC GGC GGT TTT ATA CCG CAT AGT TAG - [1621]
TN83.126   ... ... ... ... ... ... ... ... ... ... ... ... - [1621]
TN83.1211  ... ... ... ... ... ... ... ... ... ... ... ... - [1621]
TN84.611   ... ... ... ... ... ... ... ... ... ... ... ... - [1621]
TN85.123   ... ... ... ... ... ... ... ... ... ... ... ... - [1621]
TN85.417   ... ... ... ... ... ... ... ... ... ... ... ... - [1621]
TN89.356   ... ... ... ... ... ... ... ... ... ... ... ... - [1621]
TN89.1144  ... ... ... ... ... ... ... ... ... ... ... ... - [1621]
TN89.1259  ... ... ... ... ... ... ... ... ... ... ... ... - [1621]
TN89.515   ... ... ... ... ... ... ... ... ... ... ... ... - [1621]
TN90.140   ... ... ... ... ... ... ... ... ... ... ... ... - [1621]
TN90.422   ... ... ... ... ... ... ... ... ... ... ... ... - [1621]
```

```
TN90.1031 ... ... ... ... ... ... ... ... ... ... ... ... - [1621]
TN91.316  ... ... ... ... ... ... ... ... ... ... ... ... - [1621]
TN91.320  ... ... ... ... ... ... ... ... ... ... ... ... - [1621]
TN91.334  ... ... ... ... ... ... ... ... ... ... ... ... - [1621]
TN91.349  ... ... ... ... ... ... ... ... ... ... ... ... - [1621]
TN91.451  ... ... ... ... ... ... ... ... ... ... ... ... - [1621]
TN91.521  ... ... ... ... ... ... ... ... ... ... ... ... - [1621]
TN92.1031 ... ... ... ... ... ... ... ... ... ... ... ... - [1621]
TN92.1032 ... ... ... ... ... ... ... ... ... ... ... ... - [1621]
TN92.1120 ... ... ... ... ... ... ... ... ... ... ... ... - [1621]
TN93.32   ... ... ... ... ... ... ... ... ... ... ... ... - [1621]
TN93.616  ... ... ... ... ... ... ... ... ... ... ... ... - [1621]
TN94.413  ... ... ... ... ... ... ... ... ..T ... ... ... - [1621]
NL94.1    ... ... ... ... ... ... ... ... ... ... ... ... - [1621]
TN95.252  ... ... ... ... ... ... ... ... ... ... ... ... - [1621]
TN96.115  ... ... ... ... ... ... ... ... ... ... ... ... - [1621]
TN96.35   ... ... ... ... ... ... ... ... ... ... ... ... - [1621]
TN96.213  ... ... ... ... ... ... ... ... ... ... ... ... - [1621]
TN96.44   ... ... ... ... ... ... ... ... ... ... ... ... - [1621]
TN97.235  ... ... ... ... ... ... ... ... ... ... ... ... - [1621]
TN98.512  ... ... ... ... ... ... ... ... ... ... ... ... - [1621]
TN99.419  ... ... ... ... ... ... ... ... ... ... ... ... - [1621]
CAN98.73  ... ... ... ... ... ... ... ... ... ... ... ... - [1621]
CAN98.79  ... ... ... ... ... ... ... ... ... ... ... ... - [1621]
CAN00.13  ... ... ... ... ... ... ... ... ... ... ... ... - [1621]
TN01.28   ... ... ... ... ... ... ... ... ... ... ... ... - [1621]
```

Supplemental figure 4. Nucleotide sequence alignment of full-length F genes from subgroup B2 HMPV isolates. Sequences are arranged in chronological order from top to bottom. The letters indicate origin: "TN" = Tennessee; "CAN" = Canada; "NL" = Netherlands; and "JPS" = Japan. The first two digits of each isolate indicate the year; the next digit(s) for TN sequences indicate month of original virus isolation. Sequences from Canada, the Netherlands and Japan obtained from GenBank as described in the text. TN sequences have been deposited in GenBank.
